# Tuning Photophysical Properties by *p*-Functional Groups in Zn(II) and Cd(II) Complexes with Piperonylic Acid

**DOI:** 10.3390/molecules27041365

**Published:** 2022-02-17

**Authors:** Francisco Sánchez-Férez, Joaquim Mª Rius-Bartra, José A. Ayllón, Teresa Calvet, Mercè Font-Bardia, Josefina Pons

**Affiliations:** 1 Departament de Química, Universitat Autònoma de Barcelona, 08193 Barcelona, Spain; francisco.sanchez.ferez@uab.cat (F.S.-F.); joaquim.rius.bartra@gmail.com (J.M.R.-B.); joseantonio.ayllon@uab.cat (J.A.A.); 2 Departament de Mineralogia, Petrologia i Geologia Aplicada, Universitat de Barcelona, Martí i Franquès s/n, 08028 Barcelona, Spain; mtcalvet@ub.edu; 3 Unitat de Difracció de Raig-X, Centres Científics i Tecnològics de la Universitat de Barcelona (CCiTUB), Universitat de Barcelona, Solé i Sabarís, 1-3, 08028 Barcelona, Spain; mercef@ccit.ub.edu

**Keywords:** Zn(II) and Cd(II), 4-acetylpyridine, isonicotinamide, piperonylic acid, photoluminescence

## Abstract

Aggregation between discrete molecules is an essential factor to prevent aggregation-caused quenching (ACQ). Indeed, functional groups capable of generating strong hydrogen bonds are likely to assemble and cause ACQ and photoinduced electron transfer processes. Thus, it is possible to compare absorption and emission properties by incorporating two ligands with a different *bias* toward intra- and intermolecular interactions that can induce a specific structural arrangement. In parallel, the π electron-donor or electron-withdrawing character of the functional groups could modify the Highest Ocuppied Molecular Orbital (HOMO)–Lowest Unocuppied Molecular Orbital (LUMO) energy gap. Reactions of M(OAc)_2_·2H_2_O (M = Zn(II) and Cd(II); OAc = acetate) with 1,3-benzodioxole-5-carboxylic acid (Piperonylic acid, HPip) and 4-acetylpyridine (4-Acpy) or isonicotinamide (Isn) resulted in the formation of four complexes. The elucidation of their crystal structure showed the formation of one paddle-wheel [Zn(μ-Pip)_2_(4-Acpy)]_2_ (**1**); a mixture of one dimer and two monomers [Zn(µ-Pip)(Pip)(Isn)_2_]_2_·2[Zn(Pip)_2_(HPip)(Isn)]·2MeOH (**2**); and two dimers [Cd(μ-Pip)(Pip)(4-Acpy)_2_]_2_ (**3**) and [Cd(μ-Pip)(Pip)(Isn)_2_]_2_·MeOH (**4**). They exhibit bridged (**1**, µ_2_-η^1^:η^1^), bridged, chelated and monodentated (**2**, µ_2_-η^1^:η^1^, µ_1_-η^1^:η^1^ and µ_1_-η^1^), or simultaneously bridged and chelated (**3** and **4**, µ_2_-η^2^:η^1^) coordination modes. Zn(II) centers accommodate coordination numbers 5 and 6, whereas Cd(II) presents coordination number 7. We have related their photophysical properties and fluorescence quantum yields with their geometric variations and interactions supported by TD-DFT calculations.

## 1. Introduction

The correlation between composition, structure and properties has always been the foundation of materials design. Understanding this interplay allows to comprehend the structure–properties relationship and make significant progress in fields *inter alia* luminescence [1], sensing [2] or photoelectrical conductivity [3,4], allowing to design prominent materials. In the field of optical materials, the conception of fluorescent light emitting materials based on discrete molecular complexes provides a crucial advantage compared to their polymeric analogues as they have better solvent processability [5].

Pyridine based fluorophores have been developed as a fitting family of ligands with which to design fluorescent complexes. They excel at being sensitive to electronic perturbation and are capable of coordinating both soft and hard metal ions [6]. However, they commonly present fluorescence quenching associated with intramolecular charge transfer (ICT) and intra-ligand charge transfer (ILCT) transitions, photoinduced electron transfer (PET) processes, or aggregation-caused quenching (ACQ) by exciplex formation that promote non-radiative decays. In this scenario, a well-established strategy is to incorporate transition metal ions that can stabilize the excited state as well as minimize PET processes by fixing non-bonding electrons on the heteroatoms of the ligand and lowering the energy of the lone pair orbitals. Diamagnetic metal ions as Zn(II) and Cd(II) are able to coordinate with the fluorophore and present outstanding performance by minimizing relaxation via non-radiative decays [7,8]. Their fully populated d orbitals do not partake in electronic transitions and, therefore, ligand centered π⋯π* transitions are favored, which are desirable in the seeking of high emissive complexes [9]. In particular, being coordinated with *N*- and *O*-donor ligands, they tend to construct highly blue emissive materials [10].

In terms of their metal preferences, Zn(II) presents coordination numbers from 3 to 6, whereas Cd(II) can accommodate up to 8. Their geometries range from tetrahedral to octahedral or, in the case of Cd(II), the less common square-antiprism [11]. In complexes containing carboxylic acids and pyridine derivative ligands, coordination numbers 4–6 are preferred by Zn(II), while Cd(II) is likely to present 6 and 7. The Zn(II) center tends to form dimeric motifs as [Zn_2_(CO_2_)_2_] with two carboxylates coordinated in a bridging mode or square pyramidal [Zn_2_(CO_2_)_4_] paddle-wheels with two apical *N*-donors. Conversely, Cd(II) is likely to construct double bridged [Cd_2_(CO_2_)_2_] *cores* with four *N*-donor ligands, being the heptacoordinated environment is completed by chelate carboxylates and by designing a [Cd_2_(CO_2_)_4_]−2+2 motif. This *core* is commonly hindered for Zn(II) complexes [12]. Therefore, they provide a versatile tool to evaluate how the structure of the resulting complexes affects their photophysical properties.

In recent studies, our group has examined the structure–properties relationship of several Zn(II) and Cd(II) metal complexes with benzoic acid or piperonylic acid (HPip) and 3-phenylpyridine, 4-phenypyridine and 4-acetylpyridine (4-Acpy) [13,14]. From these studies, three dimeric [Zn_2_(CO_2_)_4_] paddle-wheel and three dimeric [Cd_2_(CO_2_)_4_]−2+2 double bridged complexes were synthesized and their crystal structures were elucidated. The UV-Vis absorption measurements identified 4-Acpy as a promising ligand to avoid ACQ by minimizing intermolecular interactions. Fluorescence experiments confirmed the minimization of PET processes through complexation by enhanced fluorescence of the complexes with respect to the free ligands. Likewise, the work performed using Hg(II), HPip and a range of pyridine derivative ligands (3-phenylpyridine, 4-phenylpyridine, 2,2′-bipyridine, 1,10-phenanthroline, 2,2′:6′,2′-terpyridine and di-(2-picolyl)amine) resulted in the formation of monomeric, dimeric and polymeric or oligomeric metal complexes [15]. Their UV-Vis measurements evinced that the aggregation effects can cause a significant shift on the absorption spectra, and the time-dependent density functional theory (TD-DFT) calculations displayed that the highest occupied molecular orbitals (HOMOs) were distributed along the Pip while the lowest occupied molecular orbitals (LUMOs) were over the pyridine ligands [15].

As a continuation of this work, in this paper, we have studied the reaction between M(OAc)_2_ (M = Zn(II) and Cd(II); OAc = acetate) with HPip and two *N*-donor ligands (dPy = 4-acetylpyridine (4-Acpy) and isonicotinamide (Isn)), considering that 4-Acpy has a good electron withdrawing group and Isn has a better electron donor group. From these reactions we have isolated one paddle-wheel complex [Zn(μ-Pip)_2_(4-Acpy)]_2_ (**1**); a mixture of one dimer and two monomers [Zn(µ-Pip)(Pip)(Isn)_2_]_2_·2[Zn(Pip)_2_(HPip)(Isn)]·2MeOH (**2**); and two dimeric complexes [Cd(μ-Pip)(Pip)(4-Acpy)_2_]_2_ (**3**) and [Cd(μ-Pip)(Pip)(Isn)_2_]_2_·MeOH (**4**). 

Besides, their molecular and supramolecular structures have been analyzed and their photophysical properties have been studied. These results have been supported by TD-DFT calculations. 

## 2. Results and Discussion

### 2.1. Synthesis and General Characterization

Complexes **1**–**4** were prepared via a combination of M(OAc)_2_·2H_2_O (M = Zn(II) and Cd(II); OAc = acetate) salts and 1,3-benzodioxole-5-carboxylic acid (Piperonylic, HPip) with dPy = 4-acetylpyridine (4-Acpy) or isonicotinamide (Isn) in MeOH as solvent at room temperature (RT). The M:Pip:dPy molar ratio used in the synthesis was 1:2:4 for complexes **1** and **3**; 1:2:1 for complex **2**; and 1:2:2 for complex **4**. The corresponding crystals suitable for X-ray crystallographic analysis were grown via slow evaporation of their mother liquors (**1**, **2** and **3**) or via recrystallization in MeOH (**4**).

All of the pure isolated metal complexes were characterized using elemental analysis, FTIR-ATR, ^1^H, and ^13^C{^1^H} NMR spectroscopies, and single crystal X-ray diffraction method. In addition, their UV-Vis and fluorescence spectra were recorded and their molar absorptivity (ε) and quantum yield ($\phi_{s}$) calculated. The elemental analysis of all of the complexes agreed with the proposed formulas.

**FTIR-ATR spectroscopy.** The FTIR-ATR spectra display the characteristic carboxylate bands of Pip ligand (S.I.: Figure S1–S4). The carboxylate bands for υ_as_(COO) appear at 1556 (**1**), 1534 and 1502 (**2**), 1539 and 1506 (**3**), or at 1539 and 1502 cm^−1^ (**4**), whereas bands for υ_s_(COO) arise at 1385 (**1**), 1385 (**2**), 1384 (**3**) and at 1385 cm^−1^ (**4**).The calculated Δ values (ν_as_(COO)-ν_s_(COO)) [16] were found to be 171 (**1**), 149 and 117 (**2**), 155 and 122 (**3**), 154 and 117 (**4**) cm^−1^. These values suggest a bidentate bridged coordination mode of the Pip ligands (μ_2_-η^1^:η^1^) in **1**, while in compounds **2**–**4** either bidentate bridged (μ_2_-η^1^:η^1^) or bridged and chelated (μ_1_-η^2^) coordination modes are inferred. All of the spectra present bands attributable to the ν(C=O) belong either to the 4-Acpy at 1703 cm^−1^ (**1**) and 1693 cm^−1^ (**3**) or to the Isn ligand at 1701 cm^−1^ (**2**) and 1704 cm^−1^ (**4**). The ν(C=O) of 4-Acpy in complexes **1** and **3** is almost unvaried compared to the free 4-Acpy (1693 cm^−1^) due to small changes in the intermolecular interactions. In contrast, the ν(C=O) of Isn in complexes **2** and **4** is blue shifted compared to the free Isn (1655 cm^−1^), which is associated with a decrease in hydrogen bonding [17]. Besides, the ν(C=O) of the protonated HPip ligand in **2** appears at 1678 cm^−1^, slightly blue shifted compared to the free HPip (1667 cm^−1^), suggesting that the hydrogen bonding is maintained [17]. Additional bands in all of the complexes attributable to the aromatic ring vibrations have also been identified [18,19].

**NMR spectroscopy.** The ^1^H and ^13^C{^1^H} NMR spectra were recorded in DMSO-*d*_6_. All of the spectra show the signals belonging to Pip and either to 4-Acpy or Isn. In the ^1^H NMR spectra of all the compounds (S.I: Figures S5–S8), the signals attributable to the Pip ligand have been assigned to the aromatic protons between 7.55 and 6.80 ppm, and to the aliphatic protons of the dioxole unit between 6.07 and 5.95 ppm (HPip: 7.54–7.00 and 6.12 ppm). The signals from the aromatic and CH_3_ protons of the 4-Acpy ligand appear between 8.82 and 7.72 or between 2.63 and 2.39 ppm (4-Acpy: 8.68, 7.69 and 2.36 ppm). The aromatic signals from Isn appear between 8.71 and 7.72 ppm. The protons from the amide group appear between 8.23 and 7.66 ppm (Isn: 8.68, 8.21, 7.73, 7.69). In addition, the ^1^H NMR spectra of **1**–**4** confirms the 2:1 (**1** and **3**), 5:4 (**2**) or 1:1 (**4**) molar ratio of the Pip with regards to the 4-Acpy or Isn ligands.

In the ^13^C{^1^H} NMR spectra, the signals corresponding to the carboxylate group of the Pip ligands have been found at 171.4 (**1**), 171.2 (**2**), 172.3 (**3**), and 172.1 (**4**) ppm (S.I: Figures S9–S12); the aromatic carbons between 150.8 and 107.5 ppm; and the aliphatic carbon of the dioxole unit between 101.8 and 101.4 ppm. The signals attributable to the C=O_4-Acpy_ at 198.1 (**1**) and 198.5 ppm (**3**), and those from the C=O_Isn,_ are at 166.3 (**2**) and 166.6 ppm (**4**). Besides, those corresponding to the -CH_3_ group are found at 27.0 (**1**) and 27.3 ppm (**3**) [18]. The correct assignment of the *C* aromatic atoms required the use of DEPT-135 experiments for complexes **1** and **2**.

### 2.2. Crystal and Extended Structure of Zn(II) Complexes

**Figure 1:**
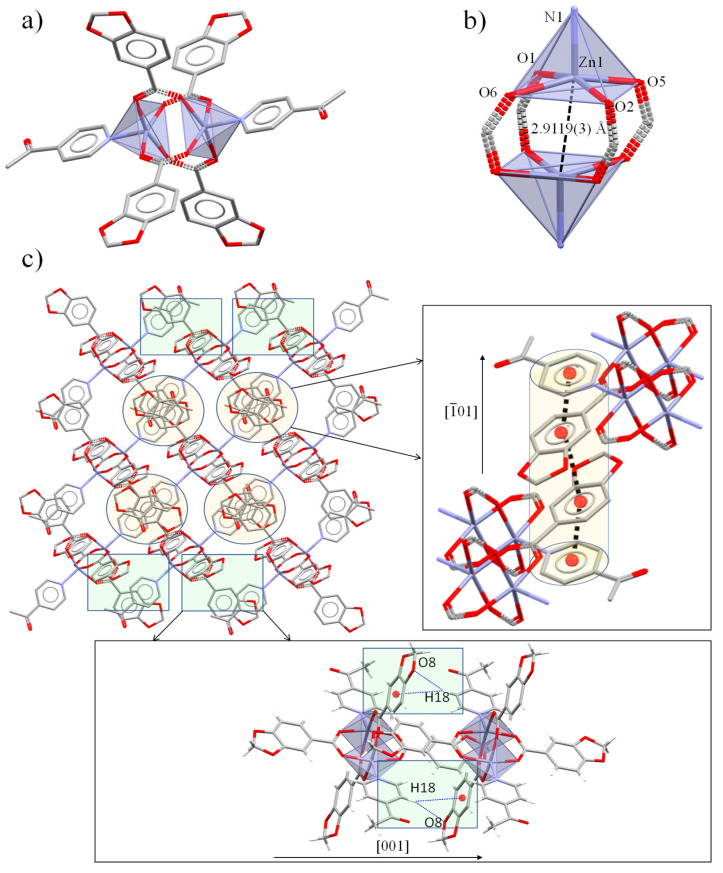
(**a**) Molecular paddle-wheel like structure of complex **1** and (**b**) Zn(II) metal core. (**c**) Supramolecular assembly forming 2D sheets promoted by π⋯π supported by C-H⋯π and C-H··O interactions. In detail, π⋯π (light orange) and C-H⋯π/C-H⋯O (light green) associations between Pip and 4-Acpy ligands.

[Zn(µ-Pip)_2_(4-Acpy])_2_]_2_ (**1**). It crystallizes in the triclinic P-1 space group and has a dimeric paddle-wheel structure (Figure 1a). Each Zn(II) center has a [ZnO_4_N] *core* belonging to the four Pip ligands and a 4-Acpy linker (Figure 1b) and exhibits a square pyramidal geometry (S.I.: Table S1) [20] with a value of τ = 0.004 [21], being pulled 0.340 Å out of the equatorial plane towards the axial position. The structure is held together by the four Pip ligands exhibiting a bidentate bridged (μ_2_-η^1^:η^1^) coordination mode. The bond lengths and angles are within the range of analogous paddle-wheel complexes [13,14] (Table 1).

These paddle-wheel units are assembled in groups of four by π⋯π interactions between Pip (Cg(2)) and 4-Acpy (Cg(3)) ligands in a 4-Acpy-Pip-Pip-4-Acpy sequence, stacking them through the [$\bar{1}$01] direction. Finally, the dimers are associated in chains along the [1] direction (Figure 1c) by weak C-H⋯π [22] and C-H⋯O interactions between the *m*-H aromatic H atom from the 4-Acpy ligand and both the aromatic ring (C18-H18⋯Cg(1), 3.395 Å) and O dioxole atom of Pip (C18-H18⋯O8, 2.598(2) Å) (Table 1, Figure 1c). All of these sets of interactions form 2D sheets of dimers along the *ac* plane.

**Figure 2:**
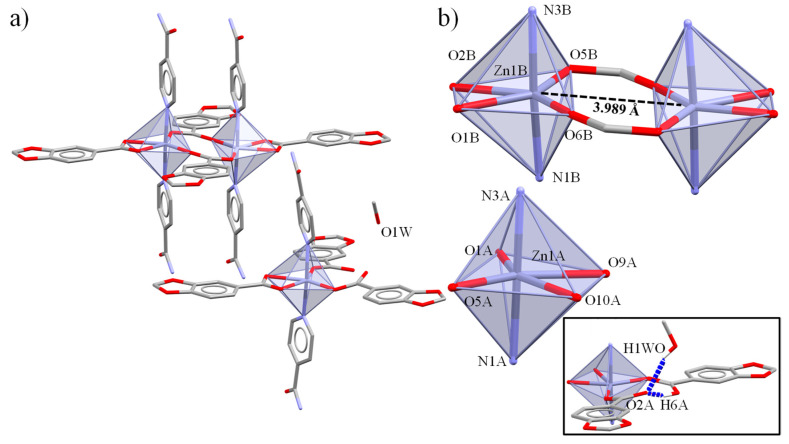
(**a**) Molecular structure of the dimeric and monomeric units in complex **2**. (**b**) Representation of the dimeric and monomeric Zn (II) cores. Inset of the intra- and intermolecular interactions between HPip and MeOH with µ_1_-η^1^-Pip ligand.

[Zn(µ-Pip)(Pip)(Isn)_2_]_2_·2[Zn(Pip)_2_(HPip)(Isn)]·2MeOH (**2**). It crystallizes in the triclinic P-1 space group and comprises one dimeric and two monomeric structural units with two occluded MeOH molecules in the unit cell (Figure 2a). Such a structure is unusual and seems to be driven by strong interactions between the amide moieties, which also promoted the same behavior with Cu(II) [23]. Both the monomer and dimer have hexacoordinated Zn(II) atoms bearing [ZnO_4_N_2_] *cores* with a distorted octahedral geometry (S.I.: Table S1) presenting an *ata* (average twist angle) of 58.41° for Zn(1A) and 58.81° for Zn(1B) [24,25] (Table 2, Figure 2b). The dimeric unit is composed of four Pip and four Isn ligands. In contrast, the monomer contains two Pip, one HPip and two Isn ligands. The Zn(II) centers in the dimer are joined by two bidentate bridged (µ_2_-η^1^:η^1^) Pip ligands while the remaining two Pip units have a bidentate chelate coordination mode (µ_1_-η^2^). Selected bond lengths and angles are displayed in Table 2. In addition, the Pip ligands in the monomer present µ_1_-η^2^ and µ_1_-η^1^ while HPip has a µ_1_-η^1^ coordination mode. This µ_1_-η^1^ coordination of the ligands is supported by an intramolecular O-H⋯O between the acidic proton of HPip and the uncoordinated carboxylate oxygen atom from the neighboring ligand. 

Simultaneously, the carboxylate O atom interacts with the MeOH molecule through an intermolecular O-H⋯O hydrogen bond. Besides, the aromatic rings from Isn stacks in an intramolecular π⋯π interaction (Table 2).

**Figure 3:**
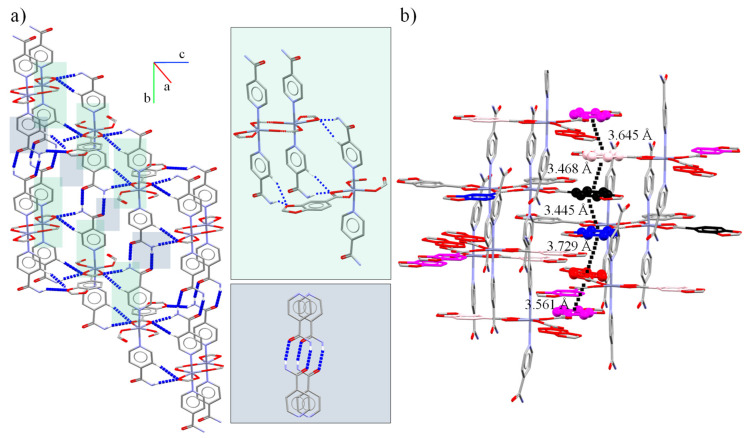
Supramolecular interactions in **2** (**a**) promoted by Isn, Pip and HPip ligands. In detail, reciprocal N-H⋯O and N-H_anti_/C-H⋯O interactions. (**b**) π⋯π interactions between Pip aromatic rings highlighted in violet (Cg(4)), pink (Cg(3)), black (Cg(7)), blue (Cg(6)), and red (Cg(5)).

The monomers and dimers form a 3D supramolecular net via amide⋯amide, N-H/C-H⋯O and π⋯π interactions (Table 3). Amide⋯amide piles monomers and dimers between themselves while Isn, through its N-H_anti_ and *m*-H, form a dimer via association with an O atom from a chelate Pip ligand of a monomer and vice versa along [22] (Figure 3a). In addition, π⋯π interactions between Pip ligands stack monomers and dimers in an ordered sequence from Cg(3) to Cg(7) along the *b* axis (Figure 3b).

### 2.3. Crystal and Extended Structure of Cd(II) Compounds

**Figure 4:**
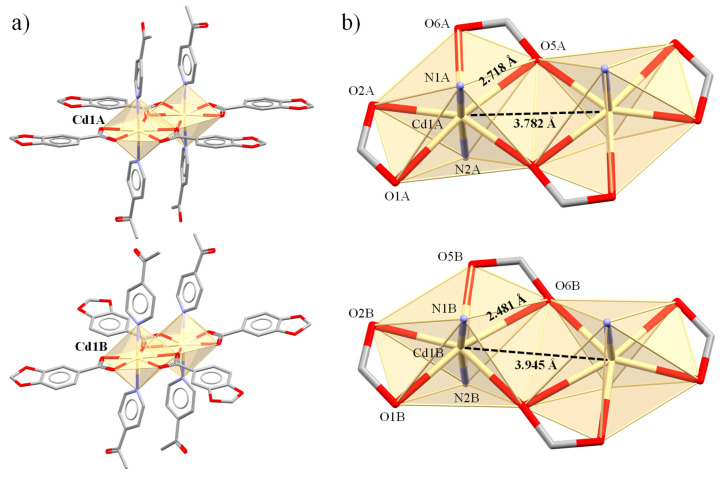
Molecular structure of the crystallographically independent dimeric units in complex **3**. Units A and B (**a**) with their corresponding Cd(II) cores (**b**).

**Figure 5:**
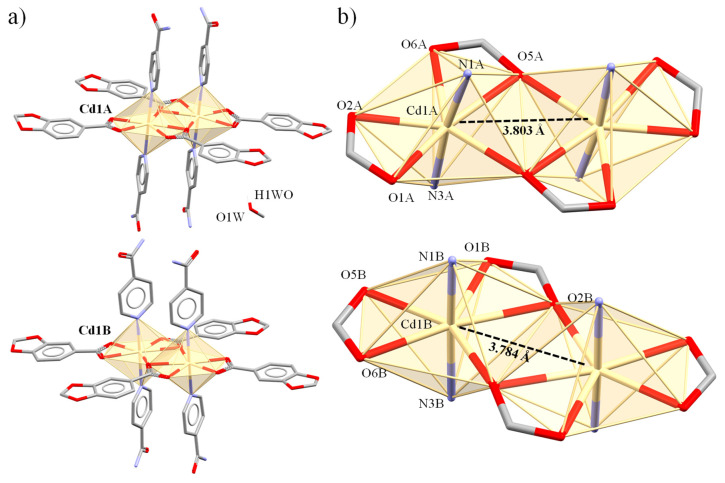
Molecular structure of the crystallographically independent dimeric units in complex **4**. Units A and B (**a**) with their corresponding Cd(II) cores (**b**).

[Cd(µ-Pip)(Pip)(4-Acpy)_2_]_2_ (**3**) and [Cd(µ-Pip)(Pip)(Isn)_2_]_2_·MeOH (**4**). They crystallize in the triclinic P-1 and monoclinic P2/c space groups, respectively, and contain two crystallographically independent molecules in the unit cell (molecules *A* and *B*). Both complexes have a dimeric structure with four Pip ligands and four 4-Acpy (**3**) (Figure 4a,b) or four Isn (**4**) (Figure 5a,b), in which the heptacoordinated Cd atoms present a [CdO_5_N_2_] *core* with a distorted pentagonal-bipyramidal geometry (S.I.: Table S1). The bond lengths and angles are within the range of analogous dimeric Cd(II) complexes [13,14] (Table 4 and Table 5).

These dimeric arrays are held together by the two Pip ligands, displaying both bidentate bridge and chelate coordination modes (μ_2_-η^2^:η^1^), and the remaining two Pip linkers complete the coordination sphere by a bidentate chelate (μ_1_-η^2^) coordination mode. Both dimers (**3** and **4**) present intramolecular π⋯π interactions between the aromatic rings of 4-Acpy and Isn, respectively (Table 6 and Table 7).

**Figure 6:**
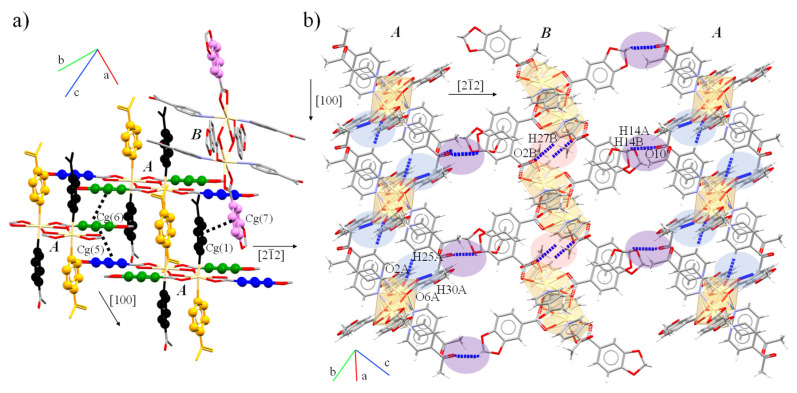
Intermolecular (**a**) π⋯π and (**b**) C-H⋯O interactions between **A** and **B** dimers present in **3**. Color codes: Cg(1) black; Cg(2) orange, Cg(5) dark blue; Cg(6) dark green; Cg(7) violet.

The intermolecular assembly of **3** is guided by π⋯π interactions supported by three pairs of weak C-H⋯O associations between the Pip and 4-Acpy ligands. The *A* dimers are stacked via planar interactions in trios, complemented by a reciprocal C-H⋯O between *m*-H and H_methyl_ from 4-Acpy and two carboxylate *O* atoms forming chains along the [100] direction. In contrast, *B* dimers only assemble via the equivalent C-H⋯O between Pip and 4-Acpy in the same axis. Finally, *A*⋯*B* dimers interact in pairs through π⋯π interactions between 4-Acpy and Pip ligands supported by C-H⋯O between the methylene *H* atoms from Pip and the *O* atoms from 4-Acpy along [2$\bar{1}$2]. Both C-H⋯O and π⋯π interactions form 2D layers in (102) (Figure 6a,b).

**Figure 7:**
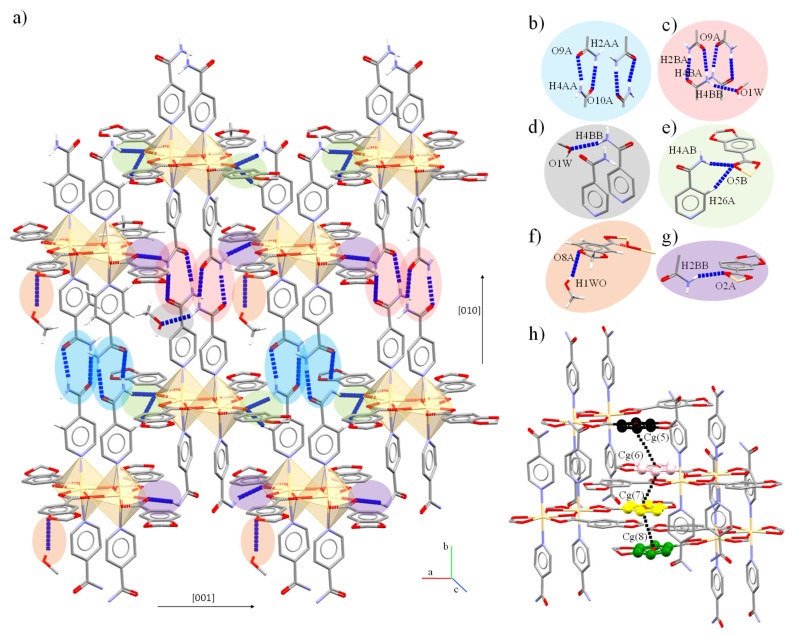
Intermolecular (**a**) N-H⋯O interactions of **4**. In detail, (**b**) amide⋯amide interactions between A dimers or (**c**) between B dimers; (**d**) MeOH⋯amide, (**e**) amide(A)⋯Pip(B) or (**f**) MeOH⋯Pip association; and (**g**) amide(B)⋯Pip(A) interactions. (**h**) π⋯π interactions between Pip rings.

The dimeric units in **4** are held together by the amide⋯amide pattern engaged in a head-to-head disposition which orders the dimers in chains (*A*⋯*A* and *B*⋯*B*) parallel to the [10] direction. Besides, the N-H_anti_ also partake in a double N-H⋯O and *m*-C-H⋯O associating the chains between *A*⋯*B* dimers (Figure 7a–g). Planar π⋯π interactions support the assembly of dimers along [10] direction (Figure 7h).

### 2.4. Structure and Geometric Evaluation 

Detailed analysis of the geometric distortions present in Zn(II) and Cd(II) complexes have been performed using SHAPE [20,26] through the *S* parameter (S.I.:Table S1). A recent structural search in the CCDC [27] has revealed that Zn(II) and Cd(II) mainly present coordination numbers ranging from 4 to 7, being scarce 3 and 8, the latter only affordable by Cd(II). The predominant structural motifs reported for Zn(II) are the dimeric µ-bridged [Zn_2_(CO_2_)_2_] units and the dimeric paddle-wheel [Zn_2_(CO_2_)_4_], bearing tetrahedral and octahedral geometries in [Zn_2_(CO_2_)_2_], or square pyramidal environments in [Zn_2_(CO_2_)_4_]. In turn, the most common motif in Cd(II) is the double bridged [Cd_2_(CO_2_)_2_], completed by two chelate ligands to form the [Cd_2_(CO_2_)_2_]−2+2 *core* and bearing coordination number 7 [12]. From these data it can be expected that complex **1** exhibits an almost ideal square pyramidal geometry (SPY-5, *S* = 0.240) [20] supported by the paddle-wheel structure that minimizes steric repulsion between the ligands. In the case of **2**, both the monomer and dimer present uncommon structural motifs as the monomeric [Zn(CO_2_)_3_] and the dimeric [Zn_2_(CO_2_)_4_]. The two display the same ligand disposition and distorted octahedral geometry, which seems to be stabilized by strong intermolecular N-H⋯O (dimer) and intramolecular O-H⋯O (monomer) interactions. In the monomeric unit, the µ_1_-η^2^ coordination mode combined with the intramolecular interaction between the HPip and Pip ligands force geometric constraints that accommodate the metal octahedral geometry (OC-6, *S* = 2.769 (**Zn1A**)). By the same token, this deviation (OC-6, *S* = 3.801 (**Zn1B**)) [20] is emphasized in the dimer by both µ_2_-η^1^:η^1^ and µ_1_-η^1^- coordination modes of the Pip ligands. Cd(II) dimers (**3** and **4**) present the common [Cd_2_(CO_2_)_2_]−2+2 *core* usually found bearing coordination number 7. Both dimers have similar *S* values (2.215 and 2.448 (**3**) or 1.995 and 2.015 (**4**)) [20] since the equatorial plane arranged by µ_2_-η^2^:η^1^ Pip linkers is almost equal. The strong double head-to-head amide⋯amide interaction between the dimers in **4** fix the Isn ligands and amend any distortion that cannot be minimized in complex **3**, in which the interactions of 4-Acpy are weaker. Overall, the smaller radius of hexacoordinated Zn(II) (0.880 Å), with respect to heptacoordinated Cd(II) (1.17 Å) [28], accentuate the geometric constraints that occur during the formation of the dimeric arrays, which is reflected in the higher *S* value (3.801) of complex **2** compared to complexes **3** and **4** (*S* range between 1.995 and 2.448) [20]. 

### 2.5. Photophysical Properties 

The absorption and emission properties of the complexes and ligands, as well as the references (L-tyrosine and phenanthrene), were recorded in a MeOH solution. The absorption was measured in the UV region of the spectra from 200 to 345 nm, while the emission was recorded between 270 and 450 nm at 298 K. 

**Figure 8:**
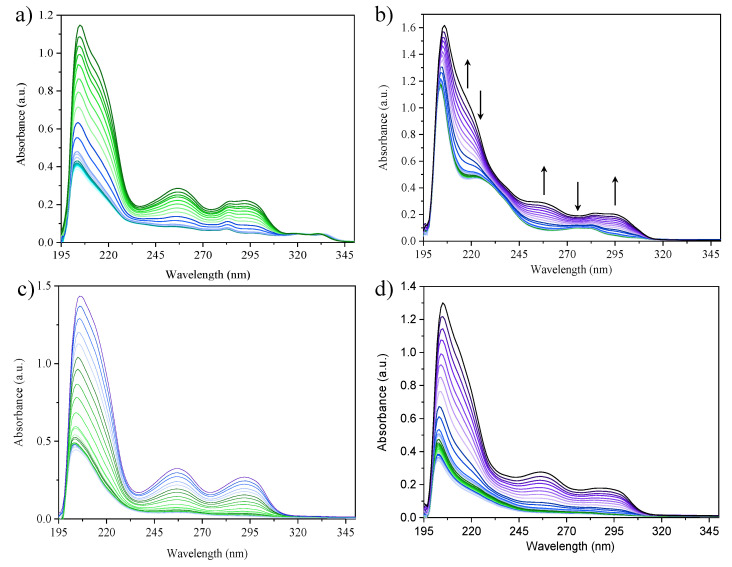
Additive UV-Vis spectra of complexes (**a**) **1**; (**b**) **2**; (**c**) **3** and (**d**) **4** performed in a concentration range from 1 × 10^−9^ M to 1 × 10^−4^ M in MeOH as the solvent at 298 K. The arrows indicate the spectral changes upon increasing concentration.

**UV-Vis spectroscopy**. To ensure the non-aggregation of the samples at the selected concentrations for the fluorescence experiments, we performed additive UV-Vis measurements within a concentration range from 1 × 10^−9^ to 1 × 10^−5^ M (Figure 8). 

The samples do not seem to present aggregation in this range of concentration. The absorption spectrum of **2** displays a significant change from 4.49 × 10^−8^ M on, which has been ascribed to a change on the absorber rather than a change via aggregates formation. This is discussed below and supported by TD-DFT calculations. The absorption and emission maxima of complexes **1**–**4** (λ_max-Abs_ and λ_max-Em_, respectively), have been identified, and their molar absorptivity (ε) and relative quantum yield (ϕ_s_) calculated (Table 8).

**Figure 9:**
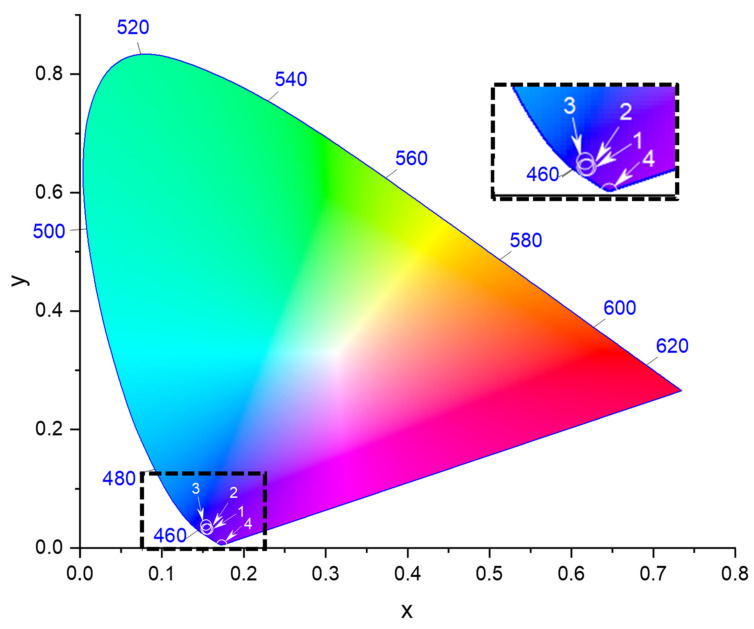
Representation of the emission color of complexes **1**–**4** within the CIE 1931 chromaticity diagram.

**Photoluminescence**. Heretofore, fluorescence measurements were performed using concentrations of 1.70 × 10^−9^ M (**1**); 1.08 × 10^−8^ M and 1.07 × 10^−7^ M (**2**); 1.04 × 10^−7^ M (**3**); and 1.01 × 10^−7^ M (**4**), extracted from the UV-Vis results after ensuring their non-aggregation to minimize ACQ [7]; these samples were excited at the wavelength of their emission maxima. All of the relevant details have been summarized in Table 8. The spectra of **1** and **2** have single emission bands at 354 nm (**1**) and 344 or 318 nm (**2**) at being irradiated at 263 nm and 225 or 251 nm, respectively. The emission spectra of **3** and **4** present unfolded emission bands centered at 355 and 347 nm, at being irradiated at 315 and 226 nm, respectively. As displayed in the CIE 1931 chromaticity diagram [29], the resultant emission color of **1**–**4** (318–355 nm) is violet (Figure 9).

**Figure 10:**
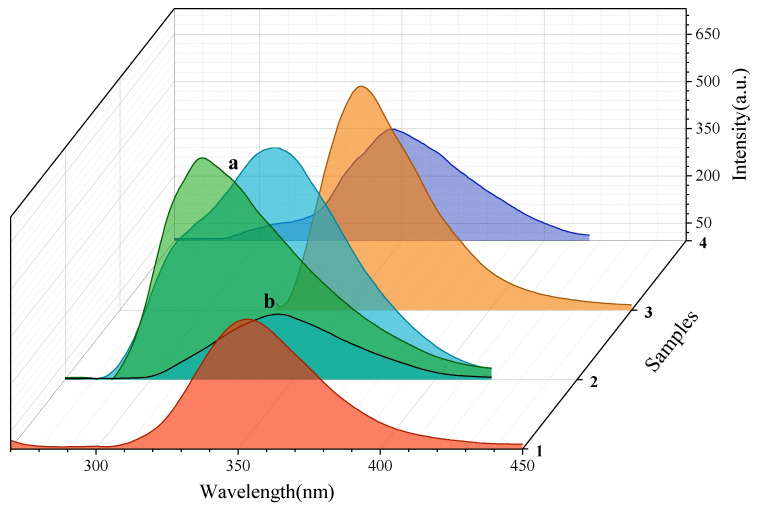
Emission spectra in MeOH at 298 K of the samples at concentrations of 1.70 × 10^−9^ M (**1**); (**a**) 1.07 × 10^−7^ M and (**b**) 1.08 × 10^−8^ M (**2**); 1.04 × 10^−7^ M (**3**); and 1.01 × 10^−7^ M (**4**). Color codes: red (**1**); light green and turquoise (**2**); orange (**3**); blue (**4**).

**Figure 11:**
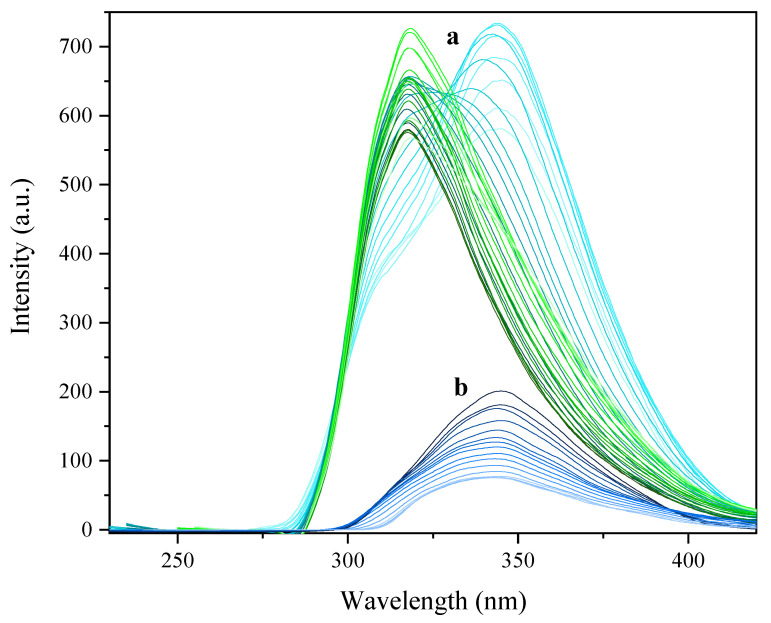
Emission spectra of complex **2**. (**a**) Sample of concentration 1.07 × 10^−7^ M irradiated from 210 to 270 nm. (**b**) Sample of concentration 1.08 × 10^−8^ M irradiated from 210 to 270 nm.

Emission intensities increase in the order **1** < **2** < **3** < **4** considering the different concentrations used in the fluorescence experiments (Figure 10). The relative quantum yields ($\phi_{s}$) of the samples were calculated by way of comparison with two reference standards (L-tyrosine and phenanthrene) [30] using Equation (1):

(1) $$\phi_{s}=\phi_{\mathrm{ref}}\left(\frac{{\mathrm{OD}}_{\mathrm{ref}}}{{\mathrm{OD}}_{s}}\right)\left(\frac{I_{s}}{I_{\mathrm{ref}}}\right){\left(\frac{n_{s}}{n_{\mathrm{ref}}}\right)}^{2}$$

where ϕ is the quantum yield, OD is the optical density (or absorbance) at the excited wavelength, I is the area under the curve of the emission spectra, and *n* is the refractive index of the solvent. In this study, L-tyrosine (ϕ_ref_ = 0.14) [31] and phenanthrene (ϕ_ref_ = 0.125) [32] has been used as the standard and their OD_ref_ and I_ref_ values have been obtained from L-tyr solutions of 1.01 × 10^−7^ M in Milli-Q water (*n*_ref_ = 1.3325) [33] and phenanthrene solutions of 1.00 × 10^−7^ in ethanol (*n*_ref_ = 1.3608) [32] as solvents at 298K. The values of $\phi_{s}$ are 0.74 (**1**), 0.47 (**3**) and 0.09 (**4**). Complex **2** present different quantum yields depending on the concentration and emission maxima. Under λ_ex_ = 225 nm at 1.08 × 10^−8^ M the $\phi_{s}$ value is 0.001 while at 1 × 10^−7^ M display $\phi_{s}=$0.02 under λ_ex_ = 225 nm and 0.03 under λ_ex_ = 251 nm (Figure 11). In addition, under exposure to black light, only complex **1** exhibits an intense yellow emission. Single crystals of **1** were irradiated under UV excitation of a pulse laser beam at λ = 326 nm and presented an emission maxima at 559 nm with a moderate Stokes shift of 12,786 cm^−1^. The solid state photoluminescence spectrum of **1** is shown in S.I: Figure S13.

### 2.6. Electronic Calculations

**DFT calculations**. The geometric optimization of complexes **1**–**4** has been performed in a MeOH solution using the Polarizable Continuum Model (PCM). The resulting complexes containing 4-Acpy exhibited an improvement of the geometric evaluator *S* from 0.240 to 0.181 (**1**) and from 2.215 and 2.448 to 2.048 and 1.889 (**3**). In contrast, complexes **2** and **4** presented significant differences between the X-ray diffraction data and the geometry in MeOH solution. The monomeric array in compound **2** is already stabilized by strong O-H⋯O intramolecular interactions between HPip and Pip ligands and suffers minor geometric changes with *S* values varying from 2.769 to 2.311. However, the dimer in **2**, which is stacked in chains by strong amide⋯amide intermolecular interactions, presents significant differences between when they are in a solid state compared to being in solution. The *S* values change from 3.801, which corresponds to an octahedral geometry, to 8.571, acquiring a trigonal prismatic geometry (S.I.: Table S2). This geometrical change can be attributed to the strong intramolecular amide⋯amide interactions between the stacked Isn ligands that force the geometry of the Zn(II) metal nodes. A similar difference between the solid state and the MeOH solution is also present in complex **4**; however, in this case, the bigger size of Cd(II) allows for a structural reorganization without such a marked difference in the *S* values, which shift from 2.015 and 1.995 to 3.367 and 2.288. Since MeOH has a solvent polarity parameter [34] ET(30) of 55.4 kcal·mol^−1,^ this could be insufficient to predominantly establish the intermolecular interactions with Isn and prevent the intramolecular amide⋯amide association. Therefore, the dimers in complexes **2** and **4** are amenable to experience significant geometric changes and promote relaxation through non-radiative decays.

**Figure 12:**
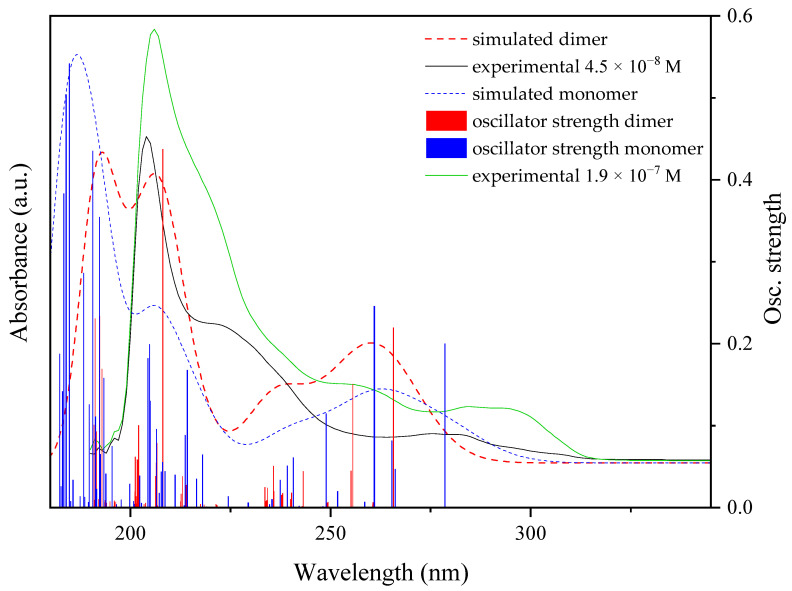
Experimental (black and green lines), calculated (dashed black and green lines) UV-Vis spectra and oscillator strengths of [Zn(µ-Pip)(Pip)(Isn)_2_]_2_·2[Zn(Pip)_2_(HPip)(Isn)]·2MeOH (**2**).

**TD-DFT calculations**. All of the calculated UV-Vis spectra of complexes **1**–**4** agree reasonably well with the experimental profiles (Figure 12, S.I.: Figures S14–S16). The shift in the theoretical absorption spectra with respect to the experimental profiles is within the range of typical TD-DFT calculations (~0.3 eV) and are caused by computing the absorptions as vertical transitions [35]. Only transitions with a higher oscillator strength (*f*) value have been selected for the molecular orbitals representation and natural transition orbitals (NTOs) analysis. The HOMO and LUMO outline as well as the energy gaps can be found in the S.I.: Figure S17. Subsequently, the main contributors of the electronic transitions have been analyzed for each absorption band to identify the regions involved in it. The molecular orbitals of each set of transitions have been represented, as well as the corresponding NTOs.

The TD-DFT results of the monomeric and dimeric forms present in complex **2** resulted in the ascription of the spectrum obtained at concentrations below 4.49 × 10^−8^ M to the monomeric form, while the shape of the spectra of the dimer is closer to the one resulting from higher concentrations. Therefore, from the electronic calculations, it could be stated that the monomer–dimer ratio is displaced towards the monomeric form at lower concentrations while a mixture of both or even primacy of the dimer is observed.

The HOMO and LUMO orbitals of **1**–**4** have π symmetry, being the HOMO along the Pip ligand while the LUMO is localized over the 4-Acpy and Isn linkers. This MO seclusion was previously observed [15] suggesting that by keeping the Pip linker constant, the incorporation of 4-Acpy ligand, bearing a more electron withdrawing functional group, would lower the energy of the LUMO and, thus, reduce the energy gap. The HOMO orbitals of these complexes are quite similar in energy, ranging from −8.312 eV to −8.018 eV since they are all located over the Pip ligands. In opposite, the LUMO orbitals present a significant difference in energy, which can be sorted into three groups. The monomer in **2** has the higher energy LUMO of −0.038 eV, while the dimers of Isn have values of −0.407 eV (**2**) and −0.535 eV (**4**), respectively. Finally, **1** and **3** dimers have the lowest energy LUMO with values of −0.905 eV (**1**) and −0.976 eV (**3**). The HOMO–LUMO gaps are between 8.274 eV and 7.244 eV, with complexes **1** and **3** presenting the smallest values.

The combined MOs (S.I.: Figures S18–S22) and NTOs (S.I.: Figures S23–S27) analysis of **1**–**4** reveals that less energetic electronic transitions of the spectra are based on local excitations (LE) and are mainly centered on the Pip ligands. Then, between 240 and 270 nm complexes **1** and **3** present LE over 4-Acpy, while **2** and **4** tend to promote charge transfer transitions between Pip and Isn and also have small contribution of MLCT to Isn. At higher energy, all but complex **1** present MLCT transitions from Pip to 4-Acpy or Isn. Since complex **1** does not present MLCT nor LMCT transitions, there appears to be a structural effect of the paddle-wheel that, in this case, minimizes electronic transitions on the Zn(II) metal center and hinders the charge transfers between Pip and 4-Acpy. The spatial arrangement of the ligands forced by the paddle-wheel leads to a greater separation between them, thus, avoiding intramolecular charge transfer transitions. The electronic transition states (TS) at the selected λ_ex_ of complexes containing Isn (**2** and **4**) present a combination of LE over Isn with a strong contribution of ILCT transitions and a small contribution of MLCT to Isn in **4** (TS20 of the monomer, TS41 of the dimer in **2** or TS23 and 24 of **4**); whereas, 4-Acpy complexes mainly present LE over 4-Acpy (TS8 and 19 of **1**) or ILCT between them (TS5 and 6 of **3**). Therefore, the combination of both intramolecular interactions and dimeric structure could directly affect the photophysical properties by promoting charge transfers instead of LE; emphasized in Isn complexes. The Supporting Information displays the complete data about geometry optimization (S.I.: Tables S3–S7 and Figures S28–S32).

## 3. Conclusions

A series of Zn(II) and Cd(II) complexes with HPip, 4-Acpy and Isn have been synthesized and fully characterized. Their crystal structure consists of one Zn(II) dimeric paddle-wheel (**1**); a mixture of one dimer and two monomers in the unit cell (**2**); and two dimers of Cd(II) (**3** and **4**). Their different nuclearity is based on different combinations of coordination modes of the Pip ligand: monodentate (µ_1_-η^1^, **2**), bidentate chelate (µ_1_-η^2^, **2**–**4**), bridged (µ_2_-η^1^:η^1^, **1** and **2**) or both (µ_2_-η^2^:η^1^, **3** and **4**) strongly influenced by intra- and intermolecular interactions. In them, Zn(II) metal node displays coordination numbers of 5 (**1**) and 6 (**2**) while Cd(II) exhibits coordination number 7 (**3** and **4**). DFT geometric optimizations revealed dPy dependent geometrical changes, emphasized by the formation of intramolecular amide⋯amide interactions. TD-DFT results show HOMO–LUMO gap dependence on the dPy, being the shortest corresponding to the 4-Acpy complexes. NTOs analysis revealed LE character of absorptions in **1** and **3**; whereas, in **2** and **4**, ILCTs have a significant contribution. This has also been reflected in the ϕ_S_ results in complex **1** being up to ten times higher than in **2**. These results reflect how (i) hampering charge transfer transition by avoiding intramolecular π⋯π interactions; (ii) minimizing PET processes through coordination to Zn(II) and Cd(II); and (iii) changes in the *p*-substituents of dPy, can modulate the HOMO–LUMO gap and maximize the resulting _S_ values.

## 4. Experimental

### 4.1. Materials and General Details

**Scheme 1:**
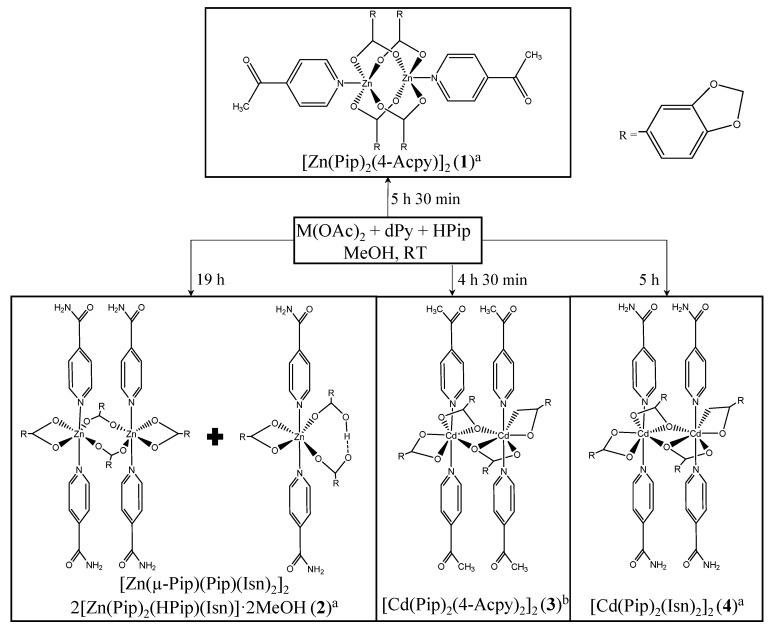
Reactions for synthesis of metal complexes **1**, **2** and **4** were performed by adding a methanolic solution of HPip over a mixture of M(OAc)_2_ and 4-Acpy or Isn^a^. The synthesis of complex **3** was achieved by adding a mixture of 4-Acpy and HPip to a methanolic solution of Cd(OAc)_2_·2H_2_O^b^.

Zinc(II) acetate dihydrate (Zn(OAc)_2_·2H_2_O), cadmium(II) acetate dihydrate (Cd(OAc)_2_·2H_2_O), 1,3-benzodioxole-5-carboxylic acid (Piperonylic, HPip), 4-acetylpyridine (4-Acpy) and isonicotinamide (Isn) ligands; and methanol (MeOH), ethanol (EtOH) and diethyl ether (Et_2_O) as solvents, were purchased from Sigma-Aldrich (Sigma Aldrich, St. Louis, MO, USA). L-tyrosine and phenanthrene were also purchased from Sigma-Aldrich, and used as the reference standard for fluorescence measurements. Deuterated dimethyl sulfoxide (dmso-*d_6_*) was purchased from Eurisotop (Euriso-Top GmbH, Saarbrücken, Germany). All of them were used without further purification. All reactions and manipulation were carried out in air at RT (Scheme 1). The elemental analyses (C, H, N) were carried out on a Euro Vector 3100 instrument (Eurovector, Pavia, Italy). The FTIR-ATR spectra were recorded on a Perkin Elmer spectrometer (Perkin Elmer Inc, Shelton, CT, USA), equipped with a universal attenuated total reflectance (ATR) accessory with a diamond window in the range of 4000–500 cm^−1^. ^1^H, ^13^C{^1^H} and DEPT-135 NMR spectra were recorded on an NMR-FT Bruker360 MHz spectrometer (Bruker BioSpin MRI GmbH, Ettlingen, Germany) spectrometer in DMSO-*d*_6_ solution at RT. All chemical shifts (δ) are given in ppm. The electronic spectra in MeOH solution were run on an Agilent HP 8453 UV-Vis spectrophotometer (Agilent, Santa Clara, CA, USA) with a quartz cell having a path length of 1 cm in the range of 190-345 nm. The molar absorptivity values were calculated and displayed as log(ε). The fluorescence measurements were carried out at 25 °C with a PerkinElmer LS 55 50 Hz fluorescence spectrometer (Perkin Elmer Inc., Shelton, CT, USA) using a 1 cm quartz cell, in MeOH solution. The samples were excited at their absorption maxima and the emission was recorded between 195 and 450 nm. The solid state photoluminescence measurement was recorded between 500 and 630 nm using a Varian Cary Eclipse Fluorescence spectrophotometer (Agilent, Santa Clara, CA, USA) and is given in nm. The UV-Vis and fluorescence spectra in solution, and the spectrum of **1** in solid state, were measured under air exposure. Both CIE 1931 chromaticity diagrams as well as the corrected dilution effects on the UV-Vis and fluorescence data were performed by means of Origin Pro 2019b software (OriginLab Corporation, Northampton, MA, USA).

### 4.2. Synthesis of Complexes **1**–**4**

[Zn(µ-Pip)_2_(4-Acpy)]_2_ (**1**). To a MeOH solution (15 mL) of Zn(OAc)_2_·2H_2_O (250 mg, 1.14 mmol), the 4-Acpy (0.500 mL, 4.13 mmol) was added drop wise, and the mixture was stirred for 5 min. Then, a MeOH solution (35 mL) of HPip (379 mg, 2.28 mmol) was added drop wise. The resulting solution was stirred for 5 h 30 min until a yellowish powder precipitated. The suspension was cooled down for 15 min and filtered off. The solid was washed twice with cold MeOH (5 mL) and dried under vacuum. Suitable colorless crystals were obtained via slow evaporation of the mother liquors for 6 days.

Yield: 365 mg (62%) (respect to Zn(OAc)_2_·2H_2_O). Elem. Anal. Calc. for C_46_H_34_Zn_2_N_2_O_18_ (1033.54 g·mol^−1^): C 53.46; H 3.32; N 2.71. Found C 53.28; H 3.05; N 2.64. FTIR-ATR (wavenumber, cm^−1^): 3107(w)–3001 [ν_ar_(C-H)], 2912(w) [ν_al_(C-H)], 1703(m) [ν(C=O)], 1638(m) [ν(C=C), ν(C=N)], 1597(m) [ν(C=C), ν(C=N)], 1556(m) [ν_as_(COO)], 1502(m), 1437(s), 1385(s) [ν_s_(COO)], 1355(m) [δ(C=C), δ(C=N], 1255(s), 1237(m), 1213(m), 1167(m) [ν(C-O-C)], 1107(m), 1078(w), 1025(s) [δ_ip_(C-H)], 930(w), 919(m), 883(m), 807(m), 771(s) [δ_oop_(C-H)], 730(m), 677(m), 588(m). ^1^H NMR (400 MHz; DMSO-*d*_6_; 298 K): δ = 8.71 [2H, d, ^3^J = 4.1 Hz, *o*-*H*]_4-Acpy_, 7.72 [2H, d, ^3^J = 4.1 Hz, *m*-*H*]_4-Acpy_, 7.43 [2H, dd, ^3^J = 8.0 Hz, ^4^J = 1.6 Hz, O_2_C-C*H*-CH], 7.27 [2H, d, ^4^J = 1.6 Hz, O_2_C-C*H*-CO], 6.80 [2H, d, ^3^J = 7.9 Hz, O_2_C-CH-C*H*], 5.95 [4H, s, O-C*H*_2_-O], 2.39 [3H, s, -CH_3_]. ^13^C{^1^H} NMR (400 MHz; DMSO-*d*_6_; 298 K): δ = 198.1 [O*C*-CH_3_], 171.4 [O_2_*C*-C], 150.8 [N-*C*H], 149.8 [O_2_C-C-(CH)_2_-*C*], 147.1 [O_2_C-C-CH-*C*], 143.1 [N-(CH)_2_-*C*], 128.9 [O_2_C-*C*], 124.7 [N-CH-*C*H], 121.8 [O_2_C-C-*C*H-CH], 109.5 [O_2_C-C-*C*H-C], 107.7 [O_2_C-C-CH-*C*H], 101.6 [O-*C*H_2_-O], 27.0 [-CH_3_]. UV-Vis in MeOH; wavelength (ε, M^−1^·cm^−1^): 203(4.93); 258(4.40); 282(4.20); 292(4.30).

[Zn(µ-Pip)(Pip)(Isn)_2_]_2_·2[Zn(Pip)_2_(HPip)(Isn)_2_]·2MeOH (**2**). To a MeOH solution (2 mL) of Zn(OAc)_2_·2H_2_O (74.9 mg, 0.341 mmol), a MeOH solution (6 mL) of Isn ligand (83.1 mg, 0.680 mmol) was added drop wise and the mixture was stirred for 5 min. Subsequently, a MeOH solution (16 mL) of HPip (114 mg, 0.682 mmol) was added drop wise. The solution was stirred for 4 h 30 min. Then, the reaction was concentrated until it reached half of the original volume, before being cooled down in an ice bath until a white powder precipitated. The resulting solid was filtered off, washed twice with cold Et_2_O (5 mL) and dried under vacuum. Suitable colorless crystals were obtained via slow evaporation of the mother liquors for 3 days.

Yield: 208 mg (83%) (respect to Zn(OAc)_2_·2H_2_O). Elem. Anal. Calc. for C_130_H_108_Zn_4_N_16_O_50_ (2955.80 g·mol^−1^): C 52.82; H 3.68; N 7.58. Found C 52.73; H 3.52; N 7.50. FTIR-ATR (wavenumber, cm^−1^): 3445(w) [ν(O-H)]_MeOH_, 3318(w) [ν_as_(N-H)], 3172(w) [ν_s_(N-H)], 3111(w)–3071 (w) [ν_ar_(C-H)], 2904(w)–2789(w) [ν_al_(C-H)], 1708(m) [ν(C=O)_Isn_], 1678(m) [ν(COOH)], 1629(m) [ν(C=C), ν(C=N)], 1607(w) [ν(C=C), ν(C=N)], 1553(m), 1534(m) [ν_as_(COO)], 1502(m) [ν_as_(COO)], 1435(s), 1417(s), 1385(s) [ν_s_(COO)], 1353(s), 1300(m) [δ(C=C), δ(C=N], 1256(s), 1240(s), 1170(m) [ν(C-O-C)], 1147(w), 1113(m), 1075(w), 1063(w), 1035(s) [δ_ip_(C-H)], 944(m), 928(m), 876(w), 861(m), 831(m), 804(m), 773(s) [δ_oop_(C-H)], 720(m), 681 (m), 641(m), 580(m), 531(m). ^1^H NMR (360 MHz; DMSO-*d*_6_; 298 K): δ = 8.66 [3.2H, d, ^3^J = 3.9 Hz, *o*-*H*]_Isn_, 8.18 [1.6H, s, OC-N*H*], 7.72 [3.1H, d, ^3^J = 4.7 Hz, N-CH-C*H*], 7.66 [1.6H, s, OC-N*H*], 7.47 [2H, d, ^3^J = 8.1 Hz, O_2_C-C-C*H*-CH], 7.30 [2H, s, O_2_C-C-C*H*-CO], 6.86 [2H, d, ^3^J = 7.5 Hz, O_2_C-C-CH-C*H*], 6.00 [4H, s, O-C*H*_2_-O], 4.03 [0.4H, br., -O*H*], 3.10 [1.2H, s, -CH_3_]. ^13^C{^1^H} NMR (360 MHz; DMSO-*d*_6_; 298 K): δ = 171.2 [O_2_*C*-C], 166.3 [O*C*-NH_2_], 150.2 [O_2_C-C-(CH)_2_-*C*], 149.6 [N-*C*H], 146.9 [O_2_C-C-CH-*C*], 141.6 [N-(CH)_2_-*C*], 128.9 [O_2_C-*C*], 124.6 [N-CH-*C*H], 121.6 [O_2_C-C-*C*H-CH], 109.4 [O_2_C-C-*C*H-C], 107.5 [O_2_C-C-CH-*C*H], 101.4 [O-*C*H_2_-O]. UV-Vis in MeOH; wavelength (ε, M^−1^·cm^−1^): 204(4.59); 225(3.24); 251(4.32); 276(4.05); 285(4.13); 292(4.26).

[Cd(µ-Pip)(Pip)(4-Acpy)_2_]_2_ (**3**). To a MeOH solution (20 mL) of Cd(OAc)_2_·2H_2_O (100 mg, 0.375 mmol), a MeOH solution (30 mL) of HPip (125 mg, 0.750 mmol) and 4-Acpy (179 mg, 1.47 mmol) was added drop wise. The resulting solution was stirred for 19 h, concentrated to half of the original volume under vacuum, and kept in the fridge for 2 days until a white precipitate was formed. The powder was filtered, washed twice with cold MeOH (10 mL) and dried under vacuum. Suitable colorless crystals were obtained via slow evaporation of the mother liquors for 8 days.

Yield: 141 mg (55%) (respect to Cd(OAc)_2_·2H_2_O). Elem. Anal. Calc. for C_60_H_48_Cd_2_N_4_O_20_ (1369.86 g·mol^−1^): C 52.61; H 3.53; N 4.09. Found C 52.34; H 3.47; N 3.95. FTIR-ATR (wavenumber, cm^−1^): 3066(w) [ν_ar_(C-H)], 3034(w) [ν_ar_(C-H)], 2905(m)–2779(w) [ν_al_(C-H)], 1693(m) [ν(C=O)], 1632(w) [ν(C=C), ν(C=N)], 1603(w) [ν(C=C), ν(C=N)], 1539(m) [ν_as_(COO)], 1506(m) [ν_as_(COO)], 1436(m), 1415(m), 1384(s) [ν_s_(COO)], 1360(s) [δ(C=C), δ(C=N)], 1254(m), 1170(w), 1113(m) [ν(C-O-C)], 1035(m) [δ_ip_(C-H)], 1014(m), 923(m), 822(m) [δ_oop_(C-H)], 806(m) [δ_oop_(C-H)], 775(s) [δ_oop_(C-H)], 682(m), 588(m), 530(s). ^1^H NMR (400 MHz; DMSO-*d*_6_; 298 K): δ = 8.82 [4H, br, *o*-*H*]_4-Acpy_, 7.82 [4H, dd, ^3^J = 6.0, ^4^J = 2.8 Hz, *m*-*H*]_4-Acpy_, 7.55 [2H, dd, ^3^J = 8.1 Hz, ^4^J = 1.6 Hz, O_2_C-C*H*-CH], 7.40 [2H, d, ^4^J = 1.4 Hz, O_2_C-C*H*-CO], 6.92 [2H, d, ^3^J = 8.1 Hz, O_2_C-CH-C*H*], 6.07 [4H, s, O-C*H*_2_-O], 2.63 [6H, s, -CH_3_]. ^13^C{^1^H} NMR (360 MHz; DMSO-*d*_6_; 298 K): δ = 198.5 [O*C* -CH_3_], 172.3 [O_2_*C*-C], 151.2 [N-*C*H], 149.9 [O_2_C-C-(CH)_2_-*C*], 147.3 [O_2_C-C-CH-*C*], 143.1 [N-(CH)_2_-*C*], 129.5 [O_2_C-*C*], 125.1 [N-CH-*C*H], 121.9 [O_2_C-C-*C*H-CH], 110.0 [O_2_C-C-*C*H-C], 107.9 [O_2_C-C-CH-*C*H], 101.8 [O-*C*H_2_-O], 27.3 [-CH_3_]. UV-Vis in MeOH; wavelength (ε, M^−1^·cm^−1^): 203(4.95); 257(4.51); 291(4.45).

[Cd(µ-Pip)_2_(Isn)_2_]_2_·MeOH (**4**). To a MeOH solution (2 mL) of Cd(OAc)_2_·2H_2_O (150 mg, 0.651 mmol), a solution (6 mL) of Isn ligand (159 mg, 1.30 mmol) was added drop wise and the mixture was stirred for 5 min. Subsequently, a MeOH solution (16 mL) of HPip (226 mg, 1.36 mmol) was added drop wise. After 5 min of stirring, a white powder precipitated. The reaction was kept under stirring conditions for 5 h. Then, it was cooled down in an ice bath and the resulting solid was filtered off, washed twice with cold Et_2_O (5 mL) and dried under vacuum. Suitable colorless crystals were obtained via recrystallization in MeOH.

Yield: 325 mg (72%) (respect to Cd(OAc)_2_·2H_2_O). Elem. Anal. Calc. for C_57_H_48_Cd_2_N_8_O_21_ (1405.85 g·mol^−1^): C 48.70; H 3.44; N 7.97. Found C 48.75; H 3.41; N 7.88. FTIR-ATR (wavenumber, cm^−1^): 3442(w) [ν(N-H)], 3316(w) [ν(N-H)], 3171(m) [ν(N-H)], 3069(w) [ν_ar_(C-H)], 2972(w)–2900(w) [ν_al_(C-H)], 1704(m) [ν(C=O)_Isn_], 1626(m) [ν(C=C), ν(C=N)], 1611(w) [ν(C=C), ν(C=N)], 1553(sh.), 1539(s) [ν_as_(COO)], 1502(m) [ν_as_(COO)], 1434(s), 1415(m), 1385(s) [ν_s_(COO)], 1349(m) [δ(C=C), δ(C=N], 1256(s), 1237(m), 1168(m) [ν(C-O-C)], 1147(w), 1108(m), 1066(w), 1039(s) [δ_ip_(C-H)], 1015(m), 942(w), 921(m), 885(w), 855(m), 825(m), 806(m), 777(s) [δ_oop_(C-H)], 723(m), 684(m), 641(s), 581(m), 530(m). ^1^H NMR (360 MHz; DMSO-*d*_6_; 298 K): δ = 8.71 [4H, d, ^3^J = 4.1 Hz, *o*-*H*]_Isn_, 8.23 [2H, s, OC-N*H*]_Isn_, 7.76 [2H, dd, ^3^J = 4.4 Hz, ^4^J = 1.8 Hz, N-CH-C*H*], 7.72 [2H, s, OC-N*H*]_Isn_, 7.54 [2H, d, ^3^J = 8.0 Hz, O_2_C-C-C*H*-CH], 7.39 [2H, s, O_2_C-C-C*H*-CO], 6.91 [2H, d, ^3^J = 8.1 Hz, O_2_C-C-CH-C*H*], 6.06 [4H, s, O-C*H*_2_-O]. ^13^C{^1^H} NMR (360 MHz; DMSO-*d*_6_; 298 K): δ = 172.1 [O_2_*C*-C], 166.6 [O*C*NH_2_], 150.5 [O_2_C-C-(CH)_2_-*C*], 149.7 [N-*C*H], 147.1 [O_2_C-C-CH-*C*], 141.6 [N-(CH)_2_-*C*], 141.1 [O_2_C-*C*], 129.2 [N-CH-*C*H], 124.9 [O_2_C-*C*], 121.8 [O_2_C-C-*C*H-CH], 109.8 [O_2_C-C-*C*H-C], 107.7 [O_2_C-C-CH-*C*H], 101.6 [O-*C*H_2_-O]. UV-Vis in MeOH; wavelength (ε, M^−1^·cm^−1^): 203(4.94); 256(4.41); 285(4.24).

### 4.3. X-ray Crystallography

Colorless (**1**, **2** and **4**) and yellow (**3**) prism-like specimens were used for the X-ray crystallographic analysis. The X-ray intensity data were measured on a D8 Venture system (Bruker, Karlsruhe, Germany) equipped with a multilayer monochromator and a Mo microfocus (λ = 0.71073 Å). For **1**–**4**, the frames were integrated with the Bruker SAINT software package (Bruker, Karlsruhe, Germany) using a narrow-frame algorithm. For **1**, the integration of the data yielded 8,790 independent reflections (average redundancy 5.448, R_sig_ = 2.65%) and 7,734 (87.99%) were greater than 2σ(|*F*|^2^). The root-mean-square (RMS) deviation in the largest hole was 0.092 e^-^/Å^3^. For **2**, the integration of the data yielded 18,771 independent reflections (average redundancy 5.125, R_sig_ = 8.69%) and 10,867 (57.89%) were greater than 2σ(|*F*|^2^). The RMS deviation in the largest hole was 0.127 e^−^/Å^3^. For **3**, the integration of the data yielded 12,559 independent reflections (average redundancy 6.025, R_sig_ = 4.93%) and 9725 (77.43%) were greater than 2σ(|*F*|^2^). The RMS deviation in the largest hole was −1.028 e^−^/Å^3^. For **4**, the integration of the data yielded 18,818 independent reflections (average redundancy 1.000, R_sig_ = 4.87%) and 12,418 (65.99%) were greater than 2σ(|*F*|^2^). The RMS deviation in the largest hole was 0.123 e^−^/Å^3^.

The structures of **1**–**4** were solved and refined using the Bruker SHELXTL Software Package (version-2018/3) [36]. The final cell constants and volume are based upon the refinement of the XYZ-centroids of reflections above 20 σ(I). The data were corrected for absorption effects using the multi-scan method (SADABS); the crystal data and relevant details of the structure refinement for compounds **1**–**4** are reported in Table 9 and Table 10. Complete information about the crystal structure and molecular geometry is available in .cif format as Supporting Information: CCDC 2124893 (**1**), 2124895 (**2**), 2124894 (**3**) and 2124892 (**4**). The molecular graphics were generated with Mercury 4.2.0 software [37] using the POV-Ray image package [38]. The color codes for all molecular graphics are: blue (Zn), yellow (Cd), light blue (N), red (O), grey (C), and white (H).

**Methodology and computational details.** All of the calculations were performed using Gaussian09 software version D.01 [39]. The geometry optimization of the ground state and vertical absorptions from the electronically excited state for **1**–**4** were completed using density functional theory (DFT) and time-dependent DFT (TD-DFT), respectively, using ωB97X-D [40,41] functional (S.I.: Tables S3–S7 and Figures S28–S32). A correlation consistent basis set was used for the Zn, Cd, C, H, N and O atoms, the effective core potential CrenbL[42]. The MeOH solvation effects were incorporated using the polarizable continuum model-Linear Response (PCM-LR) [43,44]. Since either monomeric or dimeric arrays seem to be involved in absorption and emission depending on the concentration, the geometry of the monomer and the dimer in **2** were optimized separately. The frequencies were also computed for each optimized structure to ensure that the geometries corresponded to an energy minimum. The HOMO and LUMO energetic levels were firstly examined and then the energy gaps calculated. For **1**–**4**, the first 80 vertical absorptions from the ground state to the excited states were calculated and only the most probable transitions, those with higher oscillator strength (*f*) values, were selected for the electronic analysis. The shift in the theoretical absorption spectra, with respect to the experimental profile, is within the range of typical TD-DFT calculations (~0.3 eV) and are caused by computing the absorptions as vertical transitions [35].

The methodology for the generation of the MOs was the same as has been previously reported [15] (S.I.: Figures S18–S22). The analysis of the electronic transitions was supported by the NTOs (S.I.: Figures S23–S27) [45] to better identify and represent the main contributor molecular orbitals of each transition. The NTOs were generated using Multiwfn software [46] version 3.7 with an isovalue of 0.02.

## Figures and Tables

**Table 1:** Bond lengths (Å), bond angles (°) and intermolecular interactions in **1**.

Bond Lengths						
Zn(1)-O(5)		2.0372(12)		Zn(1)-O(1)#1		2.0489(12)
Zn(1)-O(2)		2.0388(11)		Zn(1)-N(1)		2.0338(14)
Bond angles						
N(1)-Zn(1)-O(5)		101.93(5)		O(2)-Zn(1)-O(6)#1		87.85(5)
N(1)-Zn(1)-O(2)		100.22(5)		N(1)-Zn(1)-O(1)#1		98.73(5)
O(5)-Zn(1)-O(2)		89.35(5)		O(5)-Zn(1)-O(1)#1		88.98(5)
N(1)-Zn(1)-O(6)#1		97.36(6)		O(2)-Zn(1)-O(1)#1		160.91(5)
O(5)-Zn(1)-O(6)#1		160.70(6)		O(6)#1-Zn(1)-O(1)#1		87.47(5)
Intermolecular interactions		H⋯A (Å)		D⋯A (Å)	D-H (Å)	>D-H⋯A (°)
C18-H18⋯Cg(1)		3.395		4.273(3)	0.950	154.63
C18-H18⋯O8		2.598(2)		3.467(3)	0.950	152.31
π⋯π interactions						
Cg(I)⋯Cg(J)	Cg⋯Cg ^a^	α ^b^	β_,_ γ ^c^	Cg(I)_Perp,Cg(J)_Perp ^d^		Slippage ^e^
Cg(2)⋯Cg(2)	3.5075(10)	0	16.5, 16.5	3.3638(7), 3.3639(7)		0.994
Cg(2)⋯Cg(3)	3.7861(10)	10.37(8)	31.1, 20.7	3.5410(7), 3.2430(7)		-

#1 −x,−y+1,−z+1. Cg⋯Cg distances are given in Å. ^a^ Cg⋯Cg = distance between ring centroids (Å); ^b^ α = dihedral angle between planes I and J (°); ^c^ Offset angles: β = angle Cg(I)-Cg(J) and normal to plane I (°) and γ = angle Cg(I)-Cg(J) and normal to plane J (°) (β = γ, when α = 0); ^d^ Perpendicular distance (Å) of Cg(I) on plane J and perpendicular distance (Å) of Cg(J) on plane I (equal when α = 0); ^e^ Slippage = Horizontal displacement or slippage between Cg(I) and Cg(J) (equal for both centroids when α = 0). Cg(1) = C10-C11-C12-C14-C15-C16: Cg(2) = C2-C3-C4-C6-C7-C8; Cg(3) = N1-C17-C18-C19-C20-C21.

**Table 2:** Bond lengths (Å), bond angles (°) and intramolecular interactions in **2**.

Monomer						
Bond lengths						
Zn(1A)-O(1A)		2.001(3)		Zn(1A)-N(3A)		2.154(3)
Zn(1A)-O(5A)		2.052(3)		Zn(1A)-O(9A)		2.184(3)
Zn(1A)-N(1A)		2.140(3)		Zn(1A)-O(10A)		2.257(3)
Bond angles						
O(1A)-Zn(1A)-O(5A)		117.34(11)		N(1A)-Zn(1A)-O(9A)		91.03(11)
O(1A)-Zn(1A)-N(1A)		91.70(12)		N(3A)-Zn(1A)-O(9A)		89.91(10)
O(5A)-Zn(1A)-N(1A)		88.62(11)		O(1A)-Zn(1A)-O(10A)		151.96(10)
O(1A)-Zn(1A)-N(3A)		92.36(11)		O(5A)-Zn(1A)-O(10A)		90.69(10)
O(5A)-Zn(1A)-N(3A)		88.51(11)		N(1A)-Zn(1A)-O(10A)		88.07(11)
N(1A)-Zn(1A)-N(3A)		175.79(12)		N(3A)-Zn(1A)-O(10A)		88.89(10)
O(1A)-Zn(1A)-O(9A)		92.89(10)		O(9A)-Zn(1A)-O(10A)		59.09(9)
O(5A)-Zn(1A)-O(9A)		149.77(11)				
TA value (°)				ATA value (°)		
O(10A)-Cg(3)-Cg(4)-N(1A)		46.72		58.41		
N(3A)-Cg(3)-Cg(4)-O(5A)		64.48				
O(9A)-Cg(3)-Cg(4)-O(1A)		64.04				
Dimer						
Bond lengths						
Zn(1B)-O(5B)		2.025(3)		Zn(1B)-N(1B)		2.153(3)
Zn(1B)-O(6B)#1		2.034(3)		Zn(1B)-O(2B)		2.271(3)
Zn(1B)-O(1B)		2.140(3)		Zn(1B)⋯Zn(1B)		3.9893(6)
Zn(1B)-N(3B)		2.151(3)				
Bond angles						
O(5B)-Zn(1B)-O(6B)#1		125.03(11)		O(1B)-Zn(1B)-N(1B)		93.02(11)
O(5B)-Zn(1B)-O(1B)		145.44(11)		N(3B)-Zn(1B)-N(1B)		176.78(12)
O(6B)#1-Zn(1B)-O(1B)		89.46(11)		O(5B)-Zn(1B)-O(2B)		86.09(10)
O(5B)-Zn(1B)-N(3B)		88.48(11)		O(6B)#1-Zn(1B)-O(2B)		148.82(11)
O(6B)#1-Zn(1B)-N(3B)		89.27(12)		O(1B)-Zn(1B)-O(2B)		59.55(10)
O(1B)-Zn(1B)-N(3B)		90.17(11)		N(3B)-Zn(1B)-O(2B)		93.82(11)
O(5B)-Zn(1B)-N(1B)		89.12(11)		N(1B)-Zn(1B)-O(2B)		88.17(11)
O(6B)#1-Zn(1B)-N(1B)		90.34(12)				
Intramolecular interactions		H⋯A (Å)		D⋯A (Å)	D-H (Å)	>D-H⋯A (°)
O(6A)-H(6A)⋯O(2A)		1.723		2.503(4)	0.840	153.7
π⋯ π interactions						
Cg(I)⋯Cg(J)	Cg⋯Cg ^a^	α ^b^	β_,_ γ ^c^	Cg(I)_Perp, Cg(J)_Perp ^d^		Slippage ^e^
Cg(1)⋯Cg(2)	3.884(2)	1.7(2)	15.1, 16.1	3.7307(17), 3.7493(17)		-
TA value (°)				ATA value (°)		
N(3B)-Cg(5)-Cg(6)-O(5B)		68.38		58.81		
O(1B)-Cg(5)-Cg(6)-O(6B)		62.50				
O(2B)-Cg(5)-Cg(6)-N(1B)		45.55				

#1 −x+1,−y+1,−z. Cg⋯Cg distances are given in Å. ^a^ Cg⋯Cg = distance between ring centroids (Å); ^b^ α = dihedral angle between planes I and J (°); ^c^ Offset angles: β = angle Cg(I)-Cg(J) and normal to plane I (°) and γ = angle Cg(I)-Cg(J) and normal to plane J (°) (β = γ, when α = 0); ^d^ Perpendicular distance (Å) of Cg(I) on plane J and perpendicular distance (Å) of Cg(J) on plane I (equal when α = 0); ^e^ Slippage = Horizontal displacement or slippage between Cg(I) and Cg(J) (equal for both centroids when α = 0). Cg(1) = N1B-C17B-C18B-C19B-C20B-C21B; Cg(2) = N3B-C23B-C24B-C25B-C26B-C27B. Cg(3) = N3A-O9A-O10A; Cg(4) = N1A-O1A-O5A; Cg(5) = N3B-O2B-O1B; Cg(6) = N1B-O6B-O5B.

**Table 3:** Intermolecular interactions in **2**.

Intermolecular Interactions.		H⋯A (Å)		D⋯A (Å)	D-H (Å)	>D-H⋯A (°)
N(2A)-H(2AA)⋯O(14A)		2.07		2.934(4)	0.88	168.4
N(2A)-H(2BA)⋯O(9A)		2.05		2.903(4)	0.88	164
N(4A)-H(4AA)⋯O(13A)		2.04		2.921(4)	0.88	176.1
N(4A)-H(4BA)⋯O(2B)		2.02		2.855(4)	0.88	157
N(2B)-H(2BA)⋯O(10B)		1.99		2.858(4)	0.88	166.8
N(2B)-H(2BB)⋯O(12A)		2.46		3.268(5)	0.88	153.6
N(4B)-H(4BA)⋯O(9B)		2.05		2.913(4)	0.88	165.9
N(4B)-H(4BB)⋯O(10A)		2.01		2.868(4)	0.88	164.7
O(1W)-H(1WO)⋯O(2A)		2.10		2.900(6)	0.84	160.1
π⋯π interactions						
Cg(I)⋯Cg(J)	Cg⋯Cg ^a^	α ^b^	β_,_ γ ^c^	Cg(I)_Perp, Cg(J)_Perp ^d^		Slippage ^e^
Cg(7)⋯Cg(6)	3.445(2)	1.24(18)	10.2, 9.0	3.4022(16), 3.3905(14)		-
Cg(7)⋯Cg(3)	3.468(2)	3.01(18)	13.6, 15.4	3.3432(16), 3.3710(14)		-
Cg(6)⋯Cg(5)	3.729(2)	4.64(17)	25.5, 22.5	3.4456(14), 3.3670(15)		-
Cg(5)⋯Cg(4)	3.561(2)	2.91(18)	16.2, 18.6	3.3740(15), 3.4202(16)		-
Cg(4)⋯Cg(3)	3.645(2)	2.60(18)	24.2, 21.7	3.3852(16), 3.3244(14)		-

Cg⋯Cg distances are given in Å. ^a^ Cg⋯Cg = distance between ring centroids (Å); ^b^ α= dihedral angle between Planes I and J (°); ^c^ Offset angles: β = angle Cg(I)-Cg(J) and normal to plane I (°) and γ = angle Cg(I)-Cg(J) and normal to plane J (°) (β = γ, when α = 0); ^d^ Perpendicular distance (Å) of Cg(I) on plane J and perpendicular distance (Å) of Cg(J) on plane I (equal when α = 0); ^e^ Slippage = Horizontal displacement or slippage between Cg(I) and Cg(J) (equal for both centroids when α = 0). Cg(3) = C18A-C19A-C20A-C21A-C23A-C24A; Cg(4) = C10A-C11A-C12A-C13A-C15A-C16A; Cg(5) = C2A-C3A-C4A-C5A-C7A-C8A; Cg(6) = C10B-C11B-C12B-C13B-C15B-C16B; Cg(7) = C2B-C3B-C4B-C5B-C7B-C8B.

**Table 4:** Bond lengths (Å), bond angles (°) and intramolecular π⋯π interactions in **3**.

Molecule *A*					
Bond lengths					
Cd(1A)-O(2A)		2.285(2)		Cd(1A)-N(2A)	2.322(3)
Cd(1A)-O(5A)		2.330(2)		Cd(1A)-O(6A)#1	2.395(2)
Cd(1A)-O(5A)#1		2.482(2)		Cd(1A)-O(1A)	2.485(2)
Cd(1A)-N(1A)		2.332(3)		Cd(1A)⋯Cd(1A)	3.7820(5)
Bond angles					
O(2A)-Cd(1A)-N(2A)		93.46(9)		N(2A)-Cd(1A)-O(5A)#1	92.29(8)
O(2A)-Cd(1A)-O(5A)		149.25(7)		O(5A)-Cd(1A)-O(5A)#1	76.42(8)
N(2A)-Cd(1A)-O(5A)		87.09(8)		N(1A)-Cd(1A)-O(5A)#1	90.58(8)
O(2A)-Cd(1A)-N(1A)		89.75(9)		O(6A)#1-Cd(1A)-O(5A)#1	53.41(7)
N(2A)-Cd(1A)-N(1A)		172.17(9)		O(2A)-Cd(1A)-O(1A)	54.75(7)
O(5A)-Cd(1A)-N(1A)		86.51(8)		N(2A)-Cd(1A)-O(1A)	87.86(8)
O(2A)-Cd(1A)-O(6A)#1		80.79(7)		O(5A)-Cd(1A)-O(1A)	94.61(7)
N(2A)-Cd(1A)-O(6A)#1		95.65(8)		N(1A)-Cd(1A)-O(1A)	88.17(8)
O(5A)-Cd(1A)-O(6A)#1		129.79(7)		O(6A)#1-Cd(1A)-O(1A)	135.54(7)
O(2A)-Cd(1A)-O(5A)#1		134.18(7)		O(5A)#1-Cd(1A)-O(1A)	171.00(7)
**Molecule *B***					
Bond lengths					
Cd(1B)-O(5B)		2.265(2)		Cd(1B)-N(1B)	2.318(3)
Cd(1B)-O(6B)#2		2.317(2)		Cd(1B)-N(2B)	2.335(3)
Cd(1B)-O(1B)		2.366(2)		Cd(1B)-O(2B)	2.367(2)
Cd(1B)-O(6B)		2.718(2)		Cd(1B)⋯Cd(1B)	3.9452(6)
Bond angles					
O(5B)-Cd(1B)-O(6B)#2		127.84(8)		N(1B)-Cd(1B)-O(1B)	93.92(8)
O(5B)-Cd(1B)-N(1B)		96.03(9)		N(2B)-Cd(1B)-O(1B)	86.04(8)
O(6B)#2-Cd(1B)-N(1B)		85.79(8)		O(5B)-Cd(1B)-O(2B)	85.45(8)
O(5B)-Cd(1B)-N(2B)		88.90(8)		O(6B)#2-Cd(1B)-O(2B)	145.20(8)
O(6B)#2-Cd(1B)-N(2B)		86.31(8)		N(1B)-Cd(1B)-O(2B)	102.18(9)
N(1B)-Cd(1B)-N(2B)		172.09(9)		N(2B)-Cd(1B)-O(2B)	84.33(9)
O(5B)-Cd(1B)-O(1B)		141.05(7)		O(1B)-Cd(1B)-O(2B)	55.62(7)
O(6B)#2-Cd(1B)-O(1B)		90.36(8)			
π⋯ π interactions					
Cg(I)⋯Cg(J)	Cg⋯Cg ^a^	α ^b^	β_,_ γ ^c^	Cg(I)_Perp, Cg(J)_Perp ^d^	Slippage ^e^
Cg(1)⋯Cg(2)	3.7430	16.0	25.0, 10.4	3.6813, 3.3912	-
Cg(3)⋯Cg(4)	3.8415	13.0	21.2, 18.6	3.6418, 3.5810	-

#1 −x,−y+2,−z, #2 −x+1,−y,−z+1. Cg⋯Cg distances are given in Å. ^a^ Cg⋯Cg = distance between ring centroids (Å); ^b^ α = dihedral angle between planes I and J (°); ^c^ Offset angles: β = angle Cg(I)-Cg(J) and normal to plane I (°) and γ = angle Cg(I)-Cg(J) and normal to plane J (°) (β = γ, when α = 0); ^d^ Perpendicular distance (Å) of Cg(I) on plane J and perpendicular distance (Å) of Cg(J) on plane I (equal when α = 0); ^e^ Slippage = Horizontal displacement or slippage between Cg(I) and Cg(J) (equal for both centroids when α = 0). Cg(1) = N1A-C17A-C18A-C19A-C20A-C21A; Cg(2) = N2A-C24A-C25A-C26A-C27A-C28A; Cg(3) = N2B-C24B-C25B-C26B-C27B-C28B; Cg(4) = N1B-C17B-C18B-C19B-C20B-C21B.

**Table 5:** Bond lengths (Å), bond angles (°) and intramolecular π⋯π interactions of **4**.

**Molecule *A***					
Bond lengths					
Cd(1A)-N(3A)		2.307(3)		Cd(1A)-O(6A)#1	2.360(2)
Cd(1A)-N(1A)		2.331(3)		Cd(1A)-O(1A)	2.427(3)
Cd(1A)-O(5A)		2.337(2)		Cd(1A)-O(5A)#1	2.524(2)
Cd(1A)-O(2A)		2.354(2)		Cd(1A)⋯Cd(1A)	3.8026(6)
Bond angles					
N(3A)-Cd(1A)-N(1A)		173.41(9)		N(1A)-Cd(1A)-O(1A)	85.03(9)
N(3A)-Cd(1A)-O(5A)		87.98(8)		O(5A)-Cd(1A)-O(1A)	92.34(8)
N(1A)-Cd(1A)-O(5A)		85.46(8)		O(2A)-Cd(1A)-O(1A)	54.32(8)
N(3A)-Cd(1A)-O(2A)		93.79(8)		O(6A)#1-Cd(1A)-O(1A)	136.98(8)
N(1A)-Cd(1A)-O(2A)		91.36(9)		N(3A)-Cd(1A)-O(5A)#1	90.99(8)
O(5A)-Cd(1A)-O(2A)		146.66(8)		N(1A)-Cd(1A)-O(5A)#1	88.15(8)
N(3A)-Cd(1A)-O(6A)#1		91.19(8)		O(5A)-Cd(1A)-O(5A)#1	77.08(8)
N(1A)-Cd(1A)-O(6A)#1		93.53(8)		O(2A)-Cd(1A)-O(5A)#1	136.07(7)
O(5A)-Cd(1A)-O(6A)#1		130.48(8)		O(6A)#1-Cd(1A)-O(5A)#1	53.42(7)
O(2A)-Cd(1A)-O(6A)#1		82.81(8)		O(1A)-Cd(1A)-O(5A)#1	167.86(8)
N(3A)-Cd(1A)-O(1A)		94.64(9)			
**Molecule *B***					
Bond lengths					
Cd(1B)-O(2B)#2		2.306(2)		Cd(1B)-O(5B)	2.362(2)
Cd(1B)-N(3B)		2.321(2)		Cd(1B)-O(6B)	2.411(2)
Cd(1B)-O(1B)		2.328(2)		Cd(1B)-O(2B)	2.572(2)
Cd(1B)-N(1B)		2.329(3)		Cd(1B)⋯Cd(1B)	3.7838(6)
Bond angles					
O(2B)#2-Cd(1B)-N(3B)		86.45(8)		N(3B)-Cd(1B)-O(6B)	86.89(8)
O(2B)#2-Cd(1B)-O(1B)		131.21(8)		O(1B)-Cd(1B)-O(6B)	137.68(8)
N(3B)-Cd(1B)-O(1B)		92.87(9)		N(1B)-Cd(1B)-O(6B)	91.31(8)
O(2B)#2-Cd(1B)-N(1B)		86.99(8)		O(5B)-Cd(1B)-O(6B)	54.62(7)
N(3B)-Cd(1B)-N(1B)		173.17(9)		O(2B)#2-Cd(1B)-O(2B)	78.31(8)
O(1B)-Cd(1B)-N(1B)		92.89(9)		N(3B)-Cd(1B)-O(2B)	91.92(8)
O(2B)#2-Cd(1B)-O(5B)		145.66(8)		O(1B)-Cd(1B)-O(2B)	52.93(8)
N(3B)-Cd(1B)-O(5B)		91.89(8)		N(1B)-Cd(1B)-O(2B)	88.63(8)
O(1B)-Cd(1B)-O(5B)		83.13(8)		O(5B)-Cd(1B)-O(2B)	136.03(7)
N(1B)-Cd(1B)-O(5B)		92.44(8)		O(6B)-Cd(1B)-O(2B)	169.35(7)
O(2B)#2-Cd(1B)-O(6B)		91.05(8)			
π⋯ π interactions					
Cg(I)⋯Cg(J)	Cg⋯Cg ^a^	α ^b^	β_,_ γ ^c^	Cg(I)_Perp, Cg(J)_Perp ^d^	Slippage ^e^
Cg(1)⋯Cg(1)	3.6090(19)	1	14.9	3.4870(14)	-
Cg(2)⋯Cg(2)	3.6435(18)	0	18.3	3.4597(13)	1.142
Cg(3)⋯Cg(3)	3.7740(19)	1	24.4	3.4372(14)	-
Cg(4)⋯Cg(4)	3.606(2)	3	2.5	3.6021(16), 3.6023(16)	-

#1 −x + 1,y,−z + 1/2, #2 −x,y,−z + 1/2. Cg⋯Cg distances are given in Å. ^a^ Cg⋯Cg = distance between ring centroids (Å); ^b^ α= dihedral angle between Planes I and J (°); ^c^ Offset angles: β = angle Cg(I)-Cg(J) and normal to plane I (°) and γ = angle Cg(I)-Cg(J) and normal to plane J (°) (β = γ, when α = 0); ^d^ Perpendicular distance (Å) of Cg(I) on plane J and perpendicular distance (Å) of Cg(J) on plane I (equal when α = 0); ^e^ Slippage = Horizontal displacement or slippage between Cg(I) and Cg(J) (equal for both centroids when α = 0). Cg(1) = N1A-C17A-C18A-C19A-C20A-C21A; Cg(2) = N3A-C23A-C24A-C25A-C26A-C27A; Cg(3) = N1B-C17B-C18B-C19B-C20B-C21B; Cg(4) = N3B-C23B-C24B-C25B-C26B-C27B.

**Table 6:** Intermolecular interactions in **3**.

Intermolecular Interactions		H⋯A (Å)		D⋯A (Å)	D-H (Å)	>D-H⋯A (°)
C6B-H6BB⋯O10A		2.45		3.24(1)	0.99	135.9
C14B-H14B⋯O10A		2.497		3.06(1)	0.99	115.7
C25A-H25A⋯O2A		2.241		3.190(5)	0.95	177.2
C27B-H27B⋯O2B		2.365		3.228(5)	0.95	150.8
C30A-H30A⋯O6A		2.371		3.317(5)	0.98	161.9
C30B-H30E⋯O5B		2.480		3.423(6)	0.98	161.3
π⋯ π interactions						
Cg(I)⋯Cg(J)	Cg⋯Cg^a^	α ^b^	β_,_ γ ^c^	Cg(I)_Perp, Cg(J)_Perp ^d^		Slippage ^e^
Cg(1)⋯Cg(7)	3.8236	9.0	25.6, 17.1	3.6555, 3.4478		-
Cg(5)⋯Cg(6)	3.7876	1.0	29.0, 28.4	3.3325, 3.3129		-
Cg(6)⋯Cg(6)	3.5058	0	18.3, 18.3	3.3287, 3.3287		1.100

Cg⋯Cg distances are given in Å. ^a^ Cg⋯Cg = distance between ring centroids (Å); ^b^ α = dihedral angle between Planes I and J (°); ^c^ Offset angles: β = angle Cg(I)-Cg(J) and normal to plane I (°) and γ = angle Cg(I)-Cg(J) and normal to plane J (°) (β = γ, when α = 0); ^d^ Perpendicular distance (Å) of Cg(I) on plane J and perpendicular distance (Å) of Cg(J) on plane I (equal when α = 0); ^e^ Slippage = Horizontal displacement or slippage between Cg(I) and Cg(J) (equal for both centroids when α = 0). Cg(1) = N1A-C17A-C18A-C19A-C20A-C21A; Cg(5) = C2A-C3A-C4A-C5A-C7A-C8A; Cg(6) = C10A-C11A-C12A-C14A-C15A-C16A; Cg(7) = C2B-C3B-C4B-C5B-C7B-C8B.

**Table 7:** Intermolecular interactions in **4**.

Intermolecular Interactions.		H⋯A (Å)		D⋯A (Å)	D-H (Å)	>D-H⋯A (°)
N2A-H2AA⋯O10A		1.997		2.873(3)	0.88	174.1
N2A-H2AB⋯O4A		2.161		2.909(4)	0.88	142.6
N4A-H4AA⋯O9A		2.046		2.899(3)	0.88	163.1
N4A-H4AB⋯O5B		2.015		2.849(3)	0.88	157.8
N4B-H4BA⋯O9B		2.050		2.928(4)	0.88	175.7
N4B-H4BB⋯O1W		2.172		2.980(8)	0.88	152.7
N2B-H2BA⋯O10B		2.011		2.874(4)	0.88	166.2
N2B-H2BB⋯O2A		2.022		2.832(3)	0.88	152.5
C26A-H26A⋯O5B		2.418		3.315(4)	0.95	157.3
π⋯π interactions						
Cg(I)⋯Cg(J)	Cg⋯Cg ^a^	α ^b^	β_,_ γ ^c^	Cg(I)_Perp, Cg(J)_Perp ^d^		Slippage ^e^
Cg(8)⋯Cg(7)	3.560(2)	6.19(18)	8.2, 13.4	3.4625(14), 3.5233(17)		-
Cg(5)⋯Cg(6)	3.824(2)	2.39(16)	26.2, 27.7	3.3857(14), 3.4303(13)		-
Cg(6)⋯Cg(7)	3.534(2)	1.95(17)	17.3, 15.6	3.4046(13), 3.3746(17)		-

Cg⋯Cg distances are given in Å. ^a^ Cg⋯Cg = distance between ring centroids (Å); ^b^ α = dihedral angle between Planes I and J (°); ^c^ Offset angles: β = angle Cg(I)-Cg(J) and normal to plane I (°) and γ = angle Cg(I)-Cg(J) and normal to plane J (°) (β = γ, when α = 0); ^d^ Perpendicular distance (Å) of Cg(I) on plane J and perpendicular distance (Å) of Cg(J) on plane I (equal when α = 0); ^e^ Slippage = Horizontal displacement or slippage between Cg(I) and Cg(J) (equal for both centroids when α = 0). Cg(5) = C10A-C11A-C12A-C14A-C15A-C16A; Cg(6) = C2B-C3B-C4B-C5B-C7B-C8B; Cg(7) = C10B-C11B-C12B-C14B-C15B-C16B; Cg(8) = C2A-C3A-C4A-C5A-C7A-C8A.

**Table 8:** UV-Vis and fluorescence data of complexes **1**–**4**.

Sample	λ_ex_	λ_max-em_	Δλ	ϕ_s_
**1**	263	354	9774	0.74
**2**	225 225 * 251 *	344 344 * 318 *	15,375 15,375 8,394	0.01 0.03 0.02
**3**	315	355	3,577	0.47
**4**	226	347	15,429	0.09

All of the wavelengths are given in nm. Δλ are given in cm^−1^. λ_ex_ = excitation wavelength; λ_max-em_ = maximum of emission; ϕ_S_ = quantum yield. * Bands arising from a change in the absorber (the dimer in **2**).

**Table 9:** Crystal structure refinement parameters for **1** and **2**.

	1	2
Empirical formula	C_46_H_34_ZnN_2_O_18_	C_130_H_108_Zn_4_N_16_O_50_
Formula weight	1033.49	2955.80
*T* (K)	100(2)	100(2)
Wavelength (Å)	0.71073	0.71073
System, space group	Triclinic, P-1	Triclinic, P-1
Unit cell dimensions		
*a* (Å)	8.2648(5)	10.8798(6)
*b* (Å)	11.5381(6)	16.9461(8)
*c* (Å)	11.7350(7)	17.3401(9)
*α* (°)	103.454(2)	84.455(2)
*β* (°)	90.849(2)	80.089(2)
*γ* (°)	105.143(2)	77.457(2)
V (Å^3^)	1047.14(11)	3068.3(3)
*Z*	1	1
*D*_calc_ (g cm^3^)	1.639	1.600
*µ* (mm^−1^)	1.231	0.878
*F* (000)	528	1520
Crystal size (mm^3^)	0.180 × 0.120 × 0.090	0.100 × 0.080 × 0.040
*hkl* ranges	−13 ≤ h ≤ 13 −18 ≤ k ≤ 18 −18 ≤ l ≤ 18	−15 ≤ h ≤ 15 −24 ≤ k ≤ 24 −24 ≤ l ≤ 24
*θ* range (°)	1.886 to 34.487	2.076 to 30.569
Reflections collected/ unique/[Rint]	47884/8790/ [R_int_] = 0.0343	96200/18771/ [R(int) = 0.01031]
Completeness to θ (%)	99.7	99.9
Absorption Correction	Semi-empirical from equivalents	Semi-empirical from equivalents
Max. and min. transmis.	0.7468 and 0.6880	0.7461 and 0.6818
Refinement method	Full matrix least-squares on |F|^2^	Full-matrix least-squares on |F|^2^
Data/restrains/parameters	8790/0/308	18771/1/902
Goodness of fit (GOF) on |*F|*^2^	1.077	1.074
Final *R* indices [*I*>2σ(*I*)]	R_1_ = 0.0384, wR_2_ = 0.1025	R_1_ = 0.0628, wR_2_ = 0.1442
*R* indices (all data)	R_1_ = 0.0469 wR_2_ = 0.1102	R_1_ = 0.1404, wR_2_ = 0.1947
Extinction coefficient	n/a	n/a
Largest. Diff. Peak and hole (e Å^−3^)	1.540 and −0.544	2.970 and −1.091

**Table 10:** Crystal structure refinement parameters for **3** and **4**.

	3	4
Empirical formula	C_60_H_48_Cd_2_N_4_O_20_	C_113_H_92_Cd_4_N_16_O_41_
Formula weight	1369.82	2779.62
*T* (K)	100(2)	100(2)
Wavelength (Å)	0.71073	0.71073
System, space group	Triclinic, P-1	Monoclinic, P2/c
Unit cell dimensions		
*a* (Å)	10.2552(6)	15.4163(10)
*b* (Å)	11.9963(7)	17.3028(12)
*c* (Å)	24.7728(15)	22.9004(17)
*α* (°)	82.798(2)	90
*β* (°)	89.674(2)	105.565(2)
*γ* (°)	65.1870(10)	90
V (Å^3^)	2740.8(3)	5884.55
*Z*	2	2
*D*_calc_ (g cm^3^)	1.660	1.569
*µ* (mm^−1^)	0.862	0.806
*F* (000)	1384	2804
Crystal size (mm^3^)	0.131 × 0.092 × 0.042	0.400 × 0.120 × 0.040
*hkl* ranges	−13 ≤ h ≤ 13 −15 ≤ k ≤ 15 −32 ≤ l ≤ 32	−22 ≤ h ≤ 21 0 ≤ k ≤ 25 0 ≤ l ≤ 33
*θ* range (°)	2.191 to 27.526	2.190 to 31.067
Reflections collected/ unique/[Rint]	75664/12559/ [R_int_] = 0.0750	18818/18818/ [R(int) = 0.0751]
Completeness to θ (%)	99.9	99.8
Absorption Correction	Semi-empirical from equivalents	Semi-empirical from equivalents
Max. and min. transmis.	0.7455 and 0.6770	0.7454 and 0.6631
Refinement method	Full matrix least-squares on |F|^2^	Full-matrix least-squares on |F|^2^
Data/restrains/parameters	12559/0/788	18818/10/776
Goodness of fit (GOF) on |*F|*^2^	1.082	1.052
Final *R* indices [*I* > 2σ(*I*)]	R_1_ = 0.0404, wR_2_ = 0.0814	R_1_ = 0.0428, wR_2_ = 0.1115
*R* indices (all data)	R_1_ = 0.0634, wR_2_ = 0.0888	R_1_ = 0.0833, wR_2_ = 0.1439
Extinction coefficient	n/a	n/a
Largest. Diff. Peak and hole (e Å^−3^)	1.087 and −1.028	2.986 and −1.223

## Supplementary Materials

The following are available online, calculated *S* values from the crystal structures of **1**–**4**, Table S1. Calculated *S* values from the optimized geometries of **1**–**4**, Table S2. FTIR-ATR, ^1^H NMR, ^13^C{^1^H} NMR and DEPT-135 spectra, Figures S1–S12. Solid state photoluminescence spectrum of **1**, Figure S13. Comparison between experimental and theoretical UV-Vis spectra of complexes **1**, **3** and **4**, Figures S14–S16. HOMO–LUMO energy gaps, Figure S17. MOs representation of **1**–**4**, Figures S18–S22. NTOs representation of **1**–**4**, Figures S23–S27. Details about DFT optimization of **1**–**4**, Tables S3–S7 and Figures S28–S32. Complete information about the crystal structure and molecular geometry is available in .cif format as Supporting Information. CCDC 2124892–2124895 contains the supplementary crystallographic data for this paper. These data can be retrieved free of charge via www.ccdc.cam.ac.uk/data_request/cif (accessed on 15 January 2022), or by emailing data_request@ccdc.cam.ac.uk, or by contacting The Cambridge Crystallographic Data Centre, 12 Union Road, Cambridge CB2 1EZ, UK; fax: +44-1223-336033.

## Sample Availability

Samples of the compounds **1**-**4** are available from the authors.
