# Supplementary material for: Tuning Photophysical Properties by p-Functional Groups in Zn(II) and Cd(II) Complexes with Piperonylic Acid
Source: Molecules. 2022 Feb 17;27(4):1365. doi: 10.3390/molecules27041365 (PMC8876013; doi:10.3390/molecules27041365)
Supplement: Supplementary file 1 [file molecules-27-01365-s001.zip › molecules-1583345-supplementary.pdf]

Tuning photophysical properties by *p*-functional groups in Zn(II) and Cd(II) complexes with piperonylic acid

Supporting Information

Francisco Sánchez-Férez[a], Joaquim Mª Rius-Bartra[a], José A. Ayllón[a], Teresa Calvet[b], Mercè Font-Bardia[c], Josefina Pons[a,]*

[a]Departament de Química, Universitat Autònoma de Barcelona, 08193-Bellaterra, Barcelona, Spain

[b]Departament de Mineralogia, Petrologia i Geologia Aplicada, Universitat de Barcelona, Martí i Franquès s/n, 08028 Barcelona, Spain.

[c]Unitat de Difracció de Raig-X, Centres Científics i Tecnològics de la Universitat de Barcelona (CCiTUB), Universitat de Barcelona, Solé i Sabarís, 1-3, 08028 Barcelona, Spain

Tables Caption

Table S1. Geometry distortions analysis using *S* parameter calculated with SHAPE[1,2] 4
Table S2. Geometry distortions analysis using *S* parameter calculated with SHAPE[1,2] of optimized geometries in MeOH solvation.. 4
Table S3. Cartesian coordinates from X-ray and optimized geometry of **1** in MeOH................ 21
Table S4. Cartesian coordinates from X-ray and optimized geometry of the monomer present in **2** in MeOH.. 23
Table S5. Cartesian coordinates from X-ray and optimized geometry of the dimer present in **2** in MeOH.. 26
Table S6. Cartesian coordinates from X-ray and optimized geometry of **3** in MeOH................ 29
Table S7. Cartesian coordinates from X-ray and optimized geometry of **4** in MeOH................ 33

Figures Caption

Figure S1. FTIR-ATR spectrum of $[Zn(\mu\text{-}Pip)_2(4\text{-}acpy)]_2$ (**1**)... 5
Figure S2. FTIR-ATR spectrum of $[Zn(\mu\text{-}Pip)(Pip)(isn)_2]_2\cdot 2[Zn(Pip)_2(HPip)(isn)]\cdot 2MeOH$ (**2**). 5
Figure S3. FTIR-ATR spectrum of $[Cd(\mu\text{-}Pip)(Pip)(4\text{-}acpy)_2]_2$ (**3**)................................... 6
Figure S4. FTIR-ATR spectrum of $[Cd(\mu\text{-}Pip)(Pip)(isn)_2]_2$ (**4**)... 6
Figure S5. ^{1}H NMR spectrum of $[Zn(\mu\text{-}Pip)_2(4\text{-}acpy)]_2$ (**1**) recorded in dmso-*d*$_6$ at 298K........... 7
Figure S6. ^{1}H NMR spectrum of $[Zn(\mu\text{-}Pip)(Pip)(isn)_2]_2\cdot 2[Zn(Pip)_2(HPip)(isn)]\cdot 2MeOH$ (**2**) recorded in dmso-d$_6$ at 298K.. 8
Figure S7. ^{1}H NMR spectrum of $[Cd(\mu\text{-}Pip)(Pip)(4\text{-}acpy)_2]_2$ (**3**) recorded in dmso-d$_6$ at 298K... 8
Figure S8. ^{1}H NMR spectrum of $[Cd(\mu\text{-}Pip)(Pip)(isn)_2]_2$ (**4**) recorded in dmso-d$_6$ at 298K......... 9
Figure S9. ^{13}C{^{1}H} (top) and DEPT-135 (bottom) NMR spectra of $[Zn(\mu\text{-}Pip)_2(4\text{-}acpy)]_2$ (**1**) recorded in dmso-d$_6$ at 298K.. 10
Figure S10. ^{13}C{^{1}H} (top) and DEPT-135 (bottom) NMR spectra of $[Zn(\mu\text{-}Pip)(Pip)(isn)_2]_2\cdot 2[Zn(Pip)_2(HPip)(isn)]\cdot 2MeOH$ (**2**) recorded in dmso-d$_6$ at 298K. 11
Figure S11. ^{13}C{^{1}H} NMR spectrum of $[Cd(\mu\text{-}Pip)(Pip)(4\text{-}acpy)_2]_2$ (**3**) recorded in dmso-d$_6$ at 298K.. 12
Figure S12. ^{13}C{^{1}H} NMR spectra of $[Cd(\mu\text{-}Pip)(Pip)(isn)_2]_2$ (**4**) recorded in dmso-d$_6$ at 298K. 12
Figure S13. Solid state photoluminescence spectrum of complex **1** under excitation at 326 nm. 13
Figure S14. Experimental (blue line), calculated (dashed black line) UV-Vis spectra and oscillator strengths of $[Zn(\mu\text{-}Pip)_2(4\text{-}acpy)]_2$ (**1**).. 13
Figure S15. Experimental (blue line), calculated (dashed black line) UV-Vis spectra and oscillator strengths of $[Cd(\mu\text{-}Pip)(Pip)(4\text{-}acpy)_2]_2$ (**3**)... 14
Figure S16. Experimental (blue line), calculated (dashed black line) UV-Vis spectra and oscillator strengths of $[Cd(\mu\text{-}Pip)(Pip)(isn)_2]_2$ (**4**).. 14
Figure S17. Energy diagram of HOMO-LUMO gaps of complexes **1-4**. From left to right, complexes **1**, monomer in **2**, dimer in **2**, **3** and **4**. 15
Figure S18. Molecular orbitals representation for the selected electronic transition states (TS) of complex **1**. Oscillator strength values (*f*): TS4, 0.4367; TS5, 0.4366; TS8, 0.1865; TS19, 0.3567; TS35, 0.4426; TS37, 1.4664; TS49, 1.2782; TS51, 1.1540; TS60, 2.2567. 16
Figure S19. Molecular orbitals representation for the selected electronic transition states (TS) of the monomer in complex **2**. Oscillator strength values (*f*): TS1, 0.2001; TS4, 0.2458; TS7,

0.1148; TS20, 0.1680; TS44, 0.3544; TS47, 0.4349; TS51, 0.2862; TS55, 0.5419; TS56, 0.5041; TS57, 0.3833. 17
Figure S20. Molecular orbitals representation for the selected electronic transition states (TS) of the dimer in complex **2**. Oscillator strength values (*f*): TS1, 0.4391; TS5, 0.3018; TS6, 0.0894; TS11, 0.0886; TS20, 0.1015; TS41, 0.8745; TS44, 0.1579; TS53, 0.2004; TS73, 0.3385; TS75, 0.4664; TS78, 0.4614. 17
Figure S21. Molecular orbitals representation for the selected electronic transition states (TS) of complex **3**. Oscillator strength values (*f*): TS5, 0.3176; TS6, 0.1106; TS9, 0.4499; TS16, 0.1119; TS19, 0.0951; TS23, 0.0647; TS32, 0.3452; TS33, 0.0680; TS56, 0.0301. 18
Figure S22. Molecular orbitals representation for the selected electronic transition states (TS) of complex **4**. Oscillator strength values (*f*): TS1, 0.4340; TS6, 0.4243; TS17, 0.0972; TS23, 0.0743; TS24, 0.1117; TS45, 0.2965; TS47, 0.3544; TS55, 0.3980; TS56, 0.1695; TS57, 0.1686; TS74, 0.5762; TS78, 0.2031. 18
Figure S23. NTOs representation of selected electronic transition states in complex **1**. 19
Figure S24. NTOs representation of selected electronic transition states in the monomer of complex **2**. 19
Figure S25. NTOs representation of selected electronic transition states in the dimer of complex **2**. 20
Figure S26. NTOs representation of selected electronic transition states in complex **3**. 20
Figure S27. NTOs representation of selected electronic transition states in complex **4**. 20
Figure S28. Optimized geometry of **1** in MeOH solution with labelling scheme. 23
Figure S29. Optimized geometry of the monomer present in **2** in MeOH solution with labelling scheme. 26
Figure S30. Optimized geometry of the dimer present in **2** in MeOH solution with labelling scheme. 29
Figure S31. Optimized geometry of **3** in MeOH solution with labelling scheme. 33
Figure S32. Optimized geometry of **4** in MeOH solution with labelling scheme. 36

Geometry analysis

Table S1. Geometry distortions analysis using *S* parameter calculated with SHAPE[1,2]

Compound		*Geometry*[a]	*S value*
1		TBPY-5	5.681
		SPY-5	**0.240**
2	**Zn1A**	TPR-6	10.960
		OC-6	**2.769**
	**Zn1B**	TPR-6	8.780
		OC-6	**3.801**
3	**Cd1A**	PBPY-7	**2.215**
		CTPR-7	6.892
		COC-7	8.128
	**Cd1B**	PBPY-7	**2.448**
		CTPR-7	6.337
		COC-7	8.431
4	**Cd1A**	PBPY-7	**2.015**
		CTPR-7	6.739
		COC-7	7.991
	**Cd1B**	PBPY-7	**1.995**
		CTPR-7	6.748
		COC-7	8.058

Closer values have been highlighted in bold. [a]TBPY-5 = Trigonal bypyramidal; SPY-5 = Square pyramidal; PBPY-7 = Pentagonal bipyramidal; CTPR-7 = Capped trigonal prismatic; COC-7 = Capped octahedral; TPR-6 = Trigonal prismatic; OC-6 = Octahedral; PPY-6 = Pentagonal pyramidal.

Table S2. Geometry distortions analysis using *S* parameter calculated with SHAPE[1,2] of optimized geometries in MeOH solvation.

Compound		*Geometry*[a]	*S value*
1		TBPY-5	5.625
		SPY-5	**0.181**
2	**Zn1A**	TPR-6	14.038
		OC-6	**2.311**
	**Zn1B**	TPR-6	***8.571****
		OC-6	*9.778**
3	**Cd1A**	PBPY-7	**2.048**
		CTPR-7	6.707
		COC-7	8.165
	**Cd1B**	PBPY-7	**1.889**
		CTPR-7	7.452
		COC-7	9.118
4	**Cd1A**	PBPY-7	**3.367**
		CTPR-7	5.519
		COC-7	6.776
	**Cd1B**	PBPY-7	**2.288**
		CTPR-7	5.611
		COC-7	7.769

Closer values have been highlighted in bold. [a]TBPY-5 = Trigonal bypyramidal; SPY-5 = Square pyramidal; PBPY-7 = Pentagonal bipyramidal; CTPR-7 = Capped trigonal prismatic; COC-7 = Capped octahedral; TPR-6 = Trigonal prismatic; OC-6 = Octahedral; PPY-6 = Pentagonal pyramidal. *Most significant geometric changes.

FTIR-ATR spectra

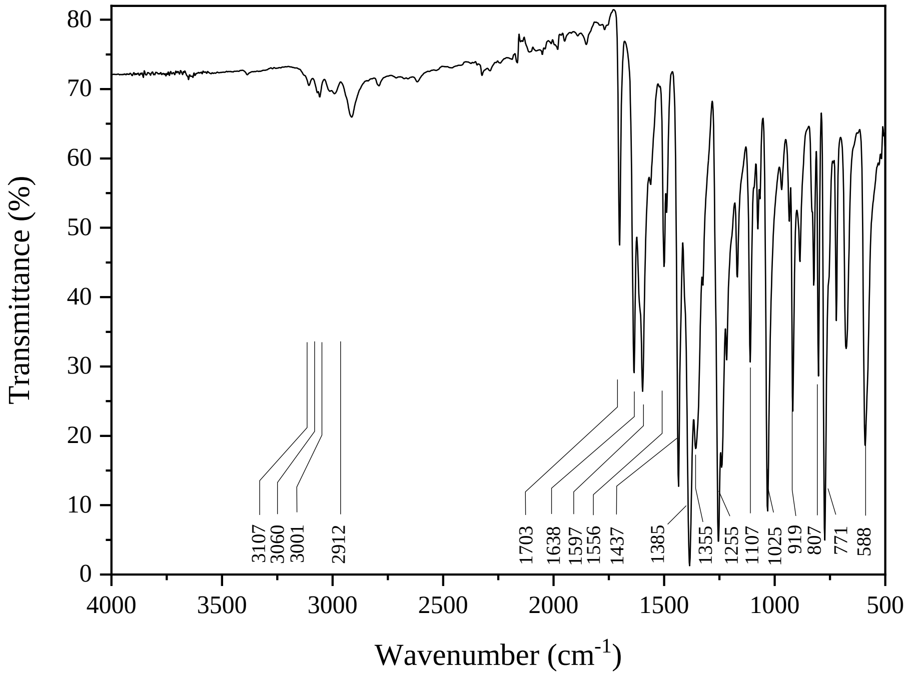

Figure S1. FTIR-ATR spectrum of [Zn(μ-Pip)$_2$(4-acpy)]$_2$ (**1**).

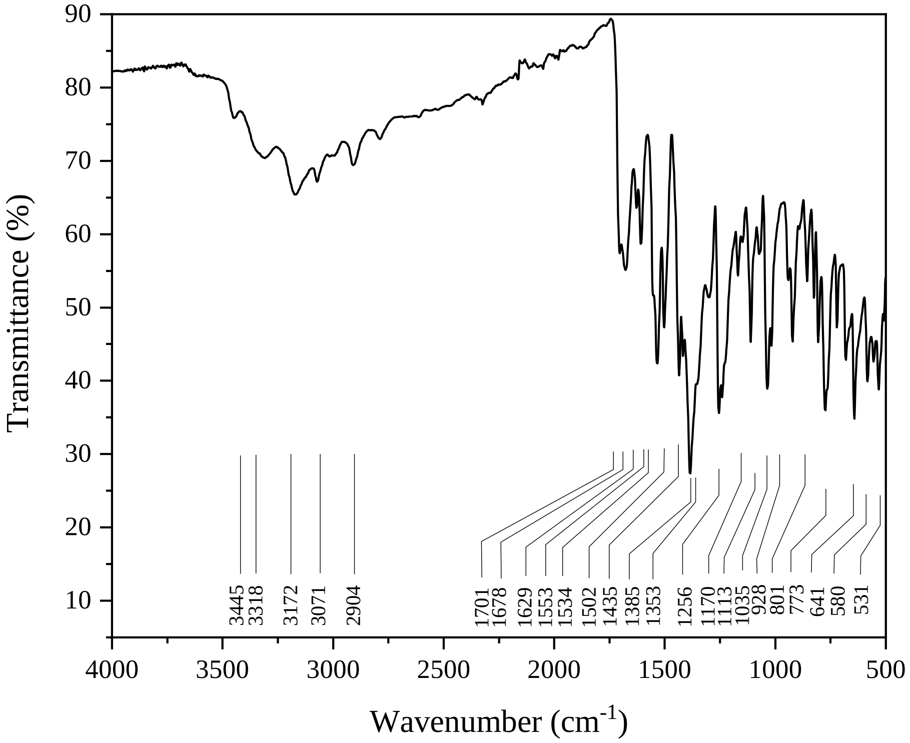

Figure S2. FTIR-ATR spectrum of [Zn(μ-Pip)(Pip)(isn)$_2$]$_2$·2[Zn(Pip)$_2$(HPip)(isn)] ·2MeOH (**2**).

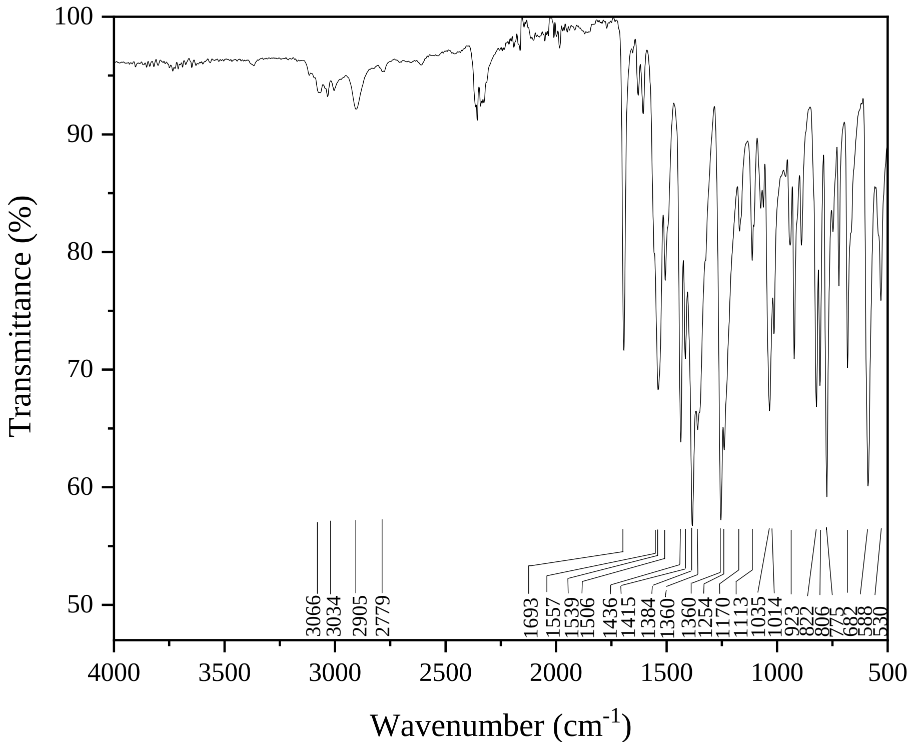

Figure S3. FTIR-ATR spectrum of $[Cd(\mu\text{-}Pip)(Pip)(4\text{-}acpy)_2]_2$ (**3**).

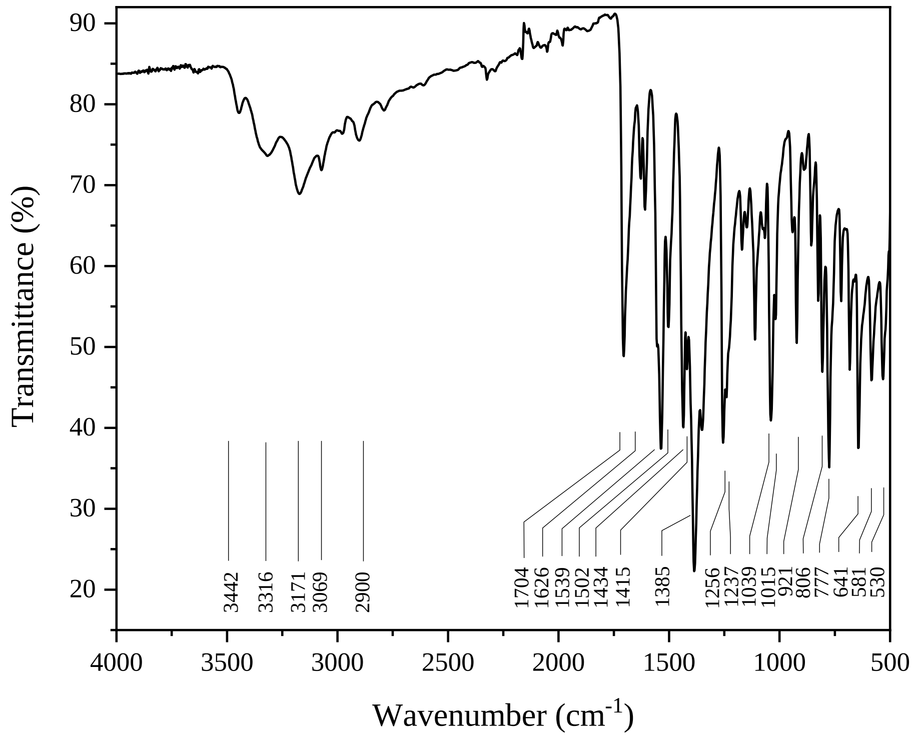

Figure S4. FTIR-ATR spectrum of $[Cd(\mu\text{-}Pip)(Pip)(isn)_2]_2$ (**4**).

^{1}H NMR spectroscopy

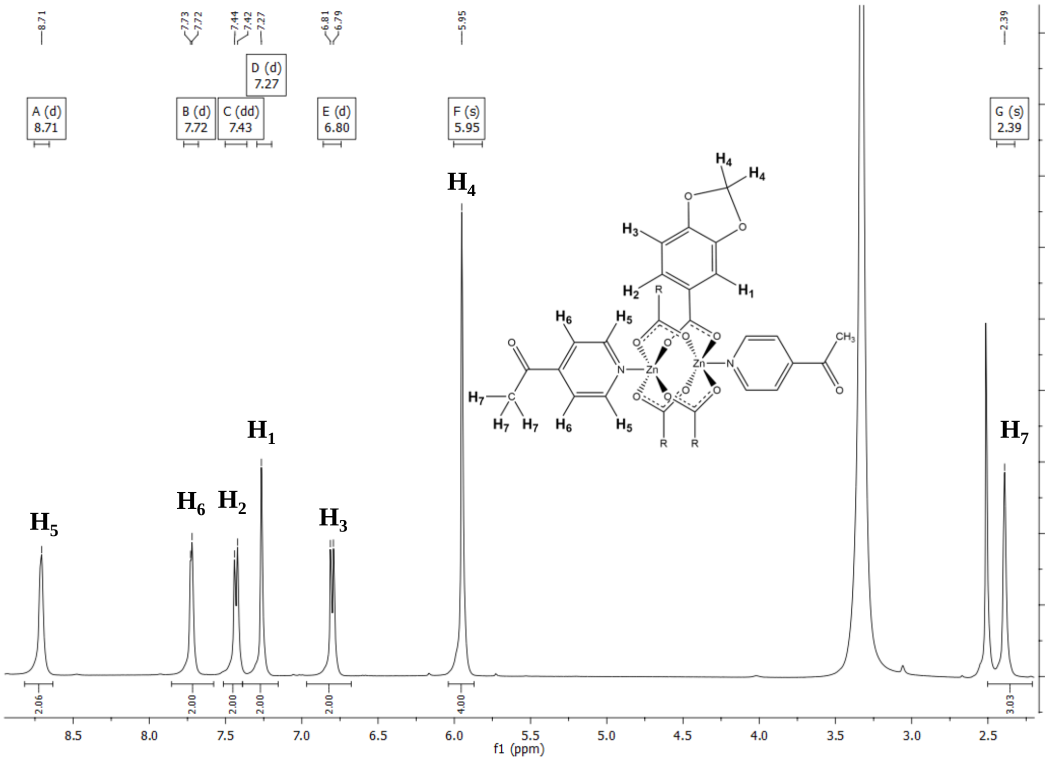

Figure S5. ^{1}H NMR spectrum of [Zn(μ-Pip)$_2$(4-acpy)]$_2$ (**1**) recorded in dmso-d_6 at 298K.

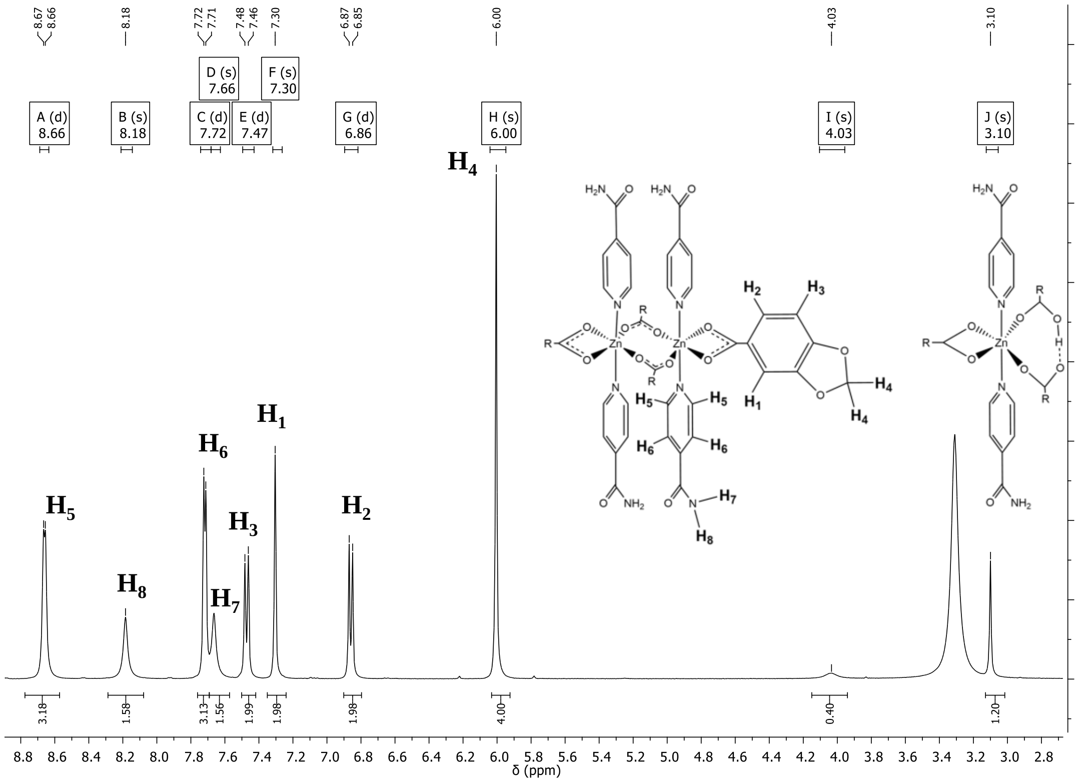

Figure S6. ^{1}H NMR spectrum of $[Zn(\mu\text{-}Pip)(Pip)(isn)_2]_2{\cdot}2[Zn(Pip)_2(HPip)(isn)]$ $\cdot 2MeOH$ (**2**) recorded in dmso-d_6 at 298K.

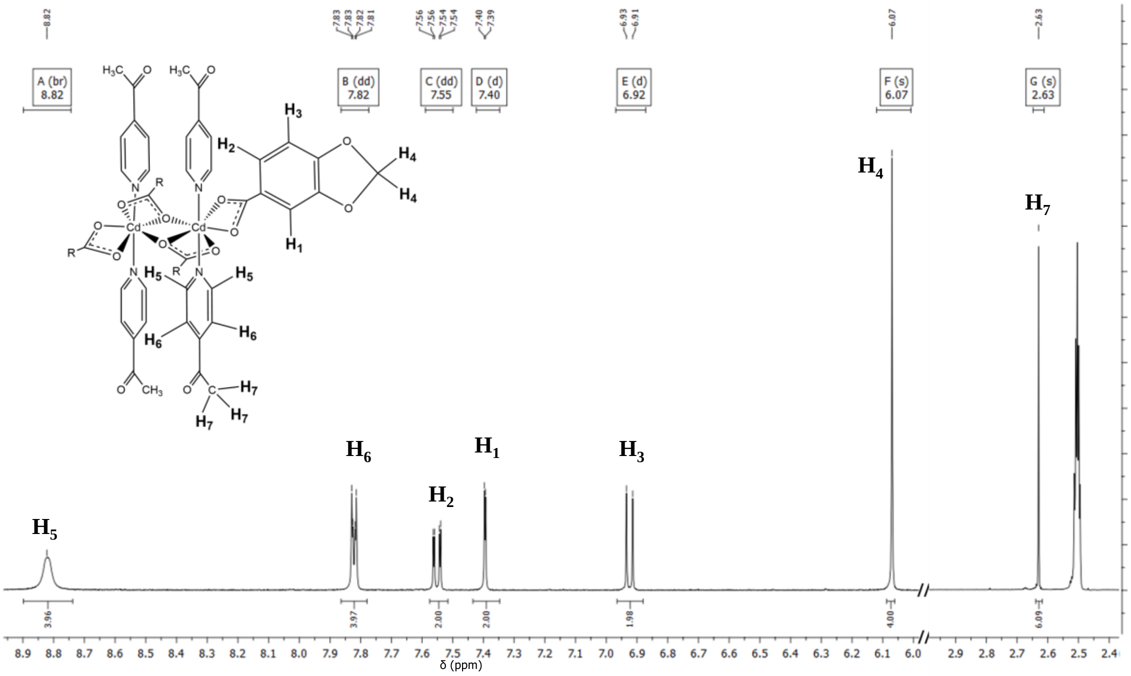

Figure S7. ^{1}H NMR spectrum of $[Cd(\mu\text{-}Pip)(Pip)(4\text{-}acpy)_2]_2$ (**3**) recorded in dmso-d_6 at 298K.

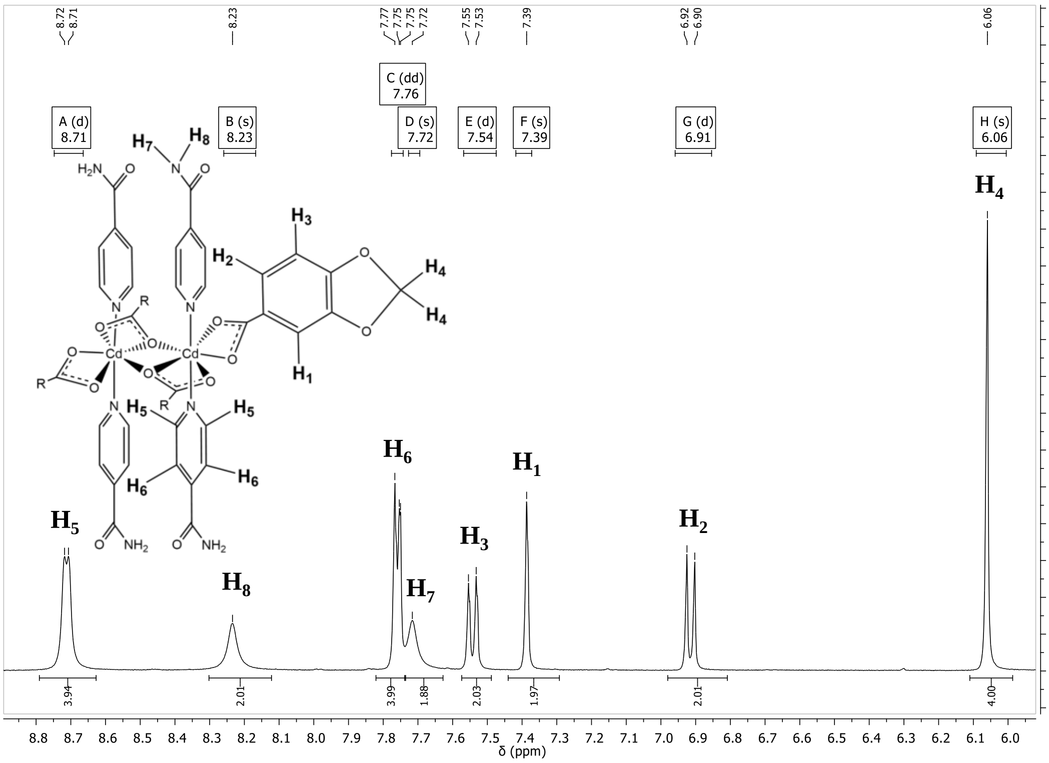

Figure S8. ^{1}H NMR spectrum of $[Cd(\mu\text{-Pip})(Pip)(isn)_2]_2$ (**4**) recorded in dmso-d_6 at 298K.

^{13}C {^{1}H} and DEPT-135 NMR spectra

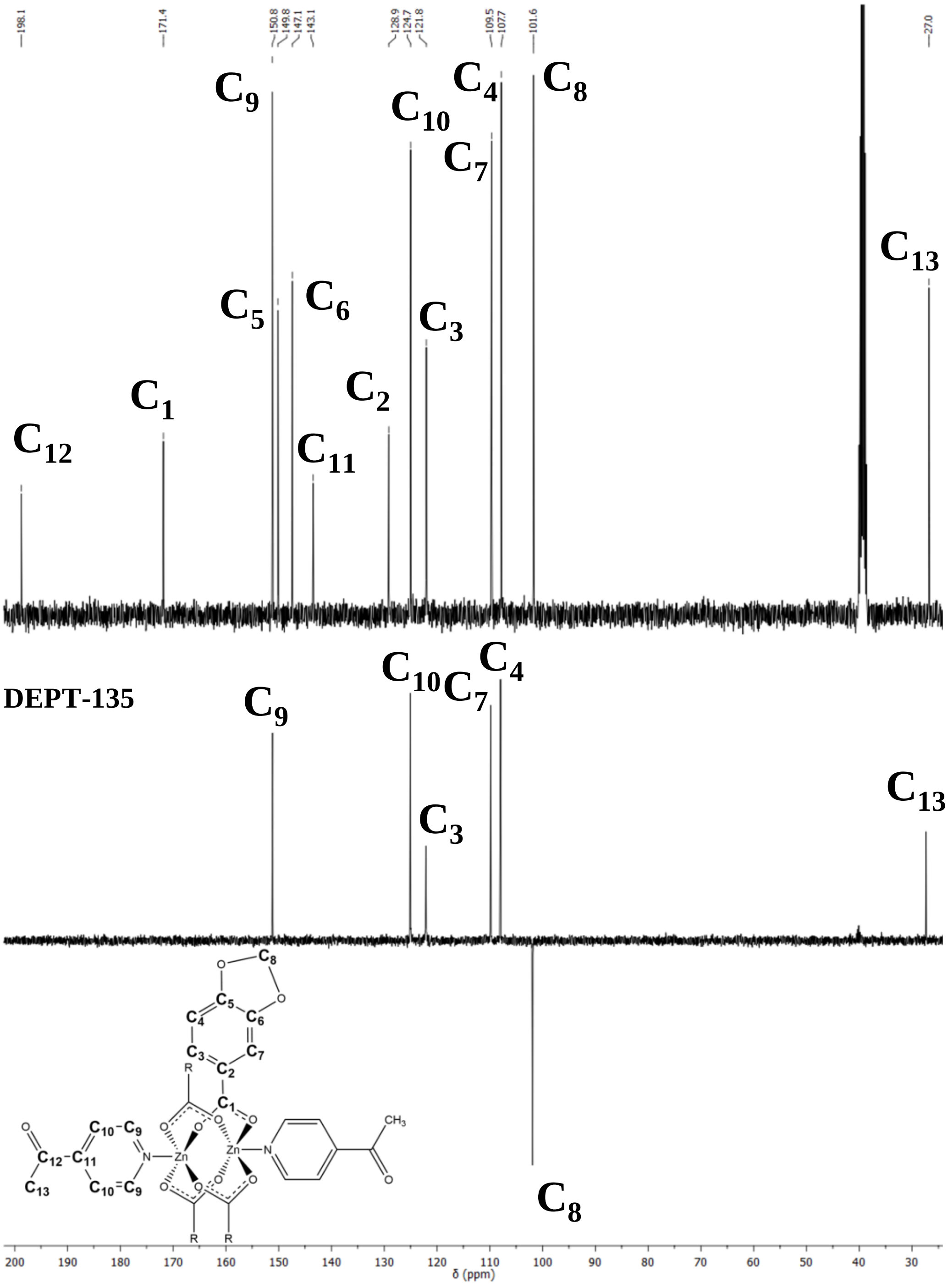

Figure S9. $^{13}C\{^{1}H\}$ (top) and DEPT-135 (bottom) NMR spectra of [Zn(μ-Pip)$_2$(4-acpy)]$_2$ (**1**) recorded in dmso-d_6 at 298K.

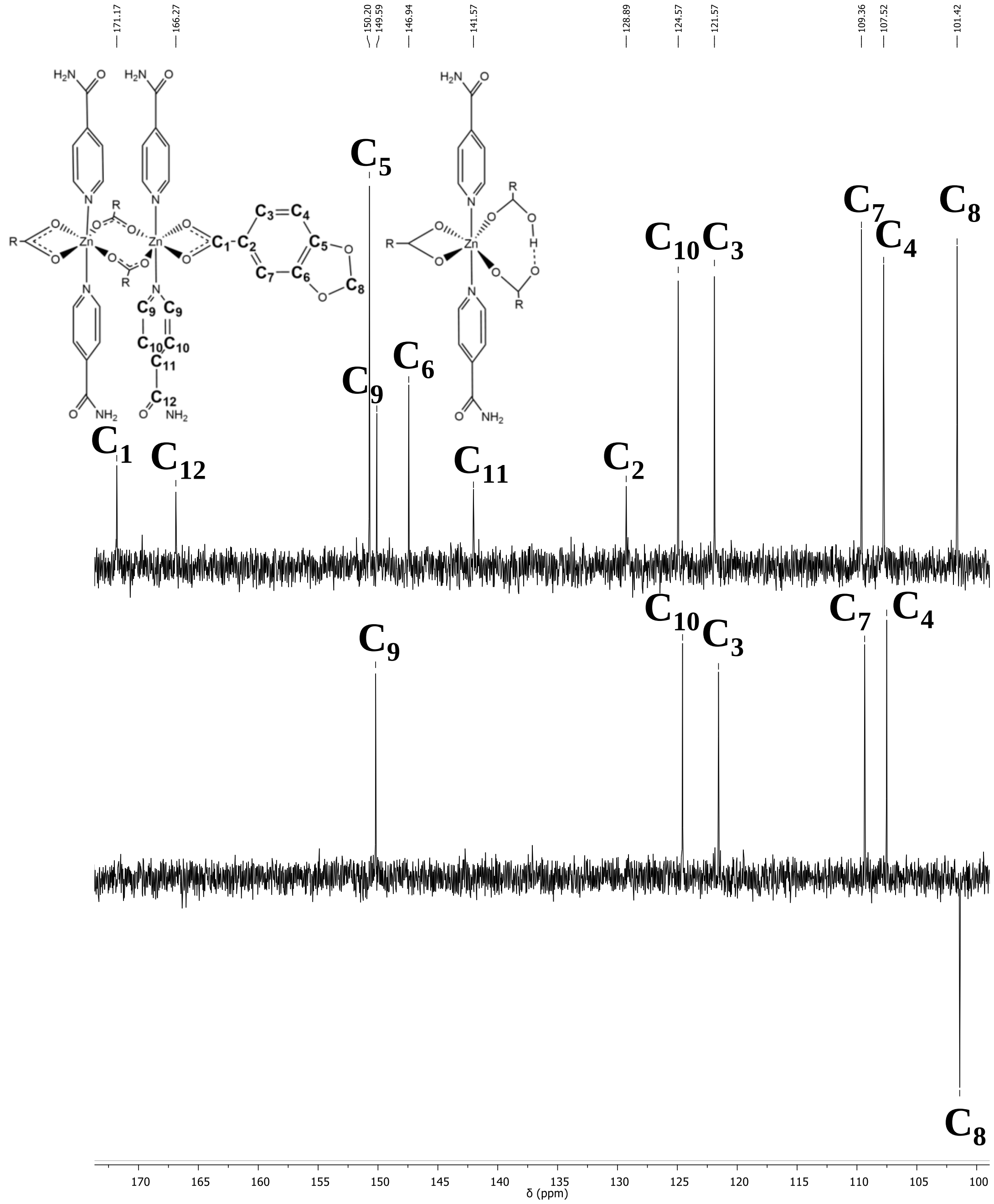

Figure S10. $^{13}C\{^{1}H\}$ (top) and DEPT-135 (bottom) NMR spectra of [Zn(μ-Pip)(Pip)(isn)$_2$]$_2$·2[Zn(Pip)$_2$(HPip)(isn)]·2MeOH (**2**) recorded in dmso-d_6 at 298K.

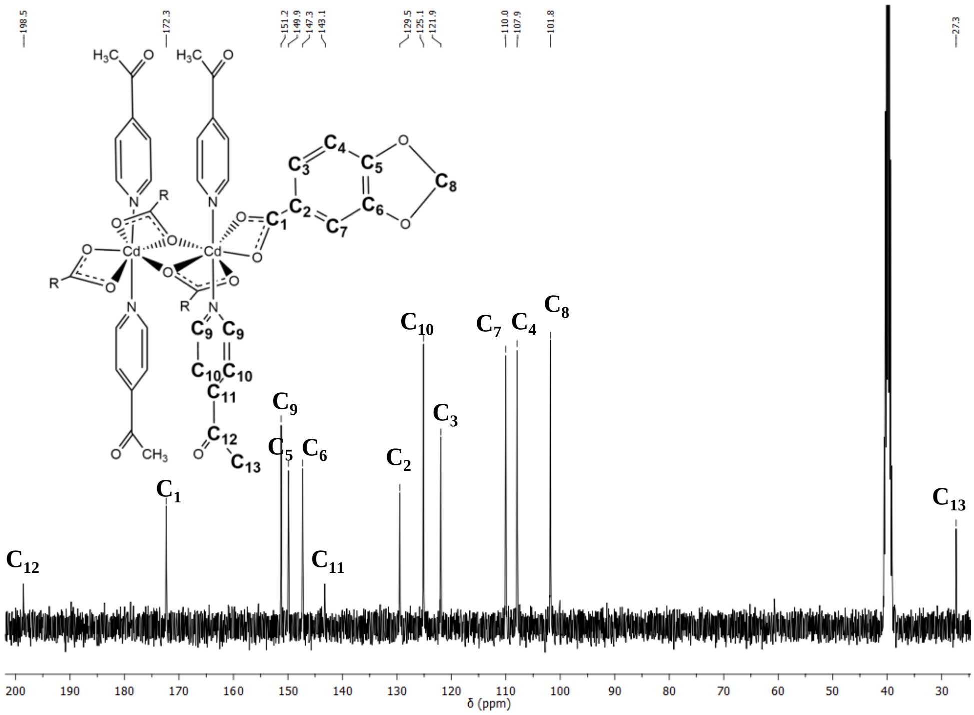

Figure S11. $^{13}C\{^{1}H\}$ NMR spectrum of [Cd(μ-Pip)(Pip)(4-acpy)$_2$]$_2$ (**3**) recorded in dmso-d_6 at 298K.

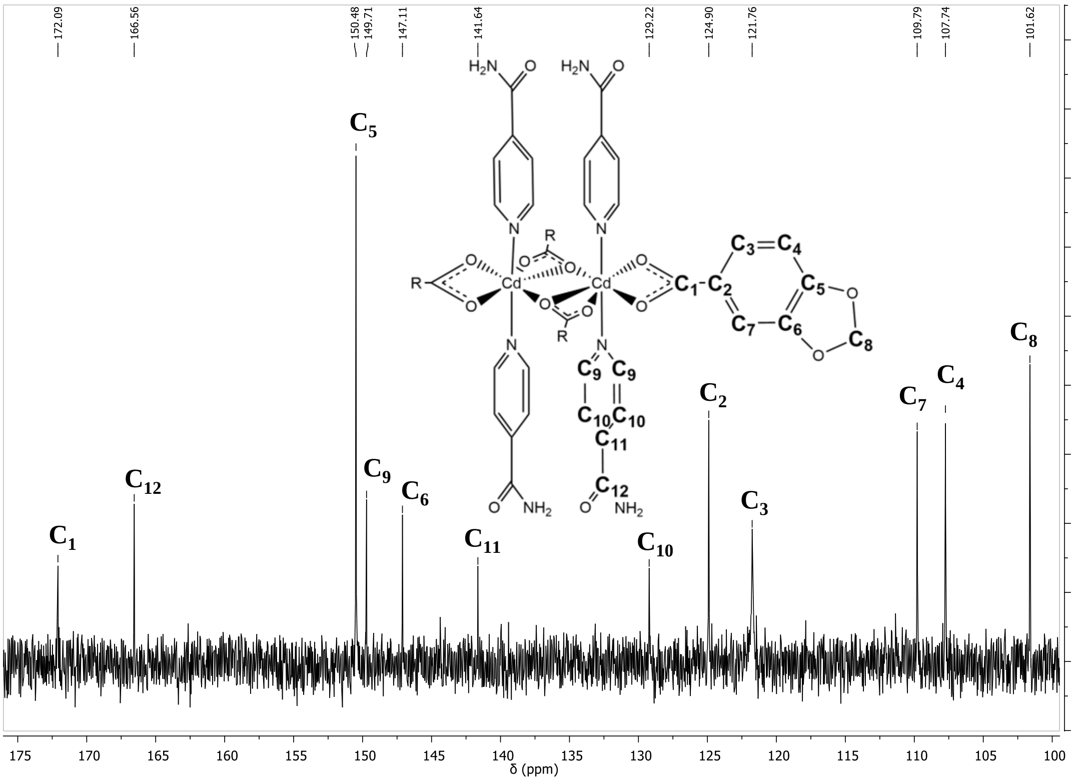

Figure S12. $^{13}C\{^{1}H\}$ NMR spectra of [Cd(μ-Pip)(Pip)(isn)$_2$]$_2$ (**4**) recorded in dmso-d_6 at 298K.

Solid state photoluminescence

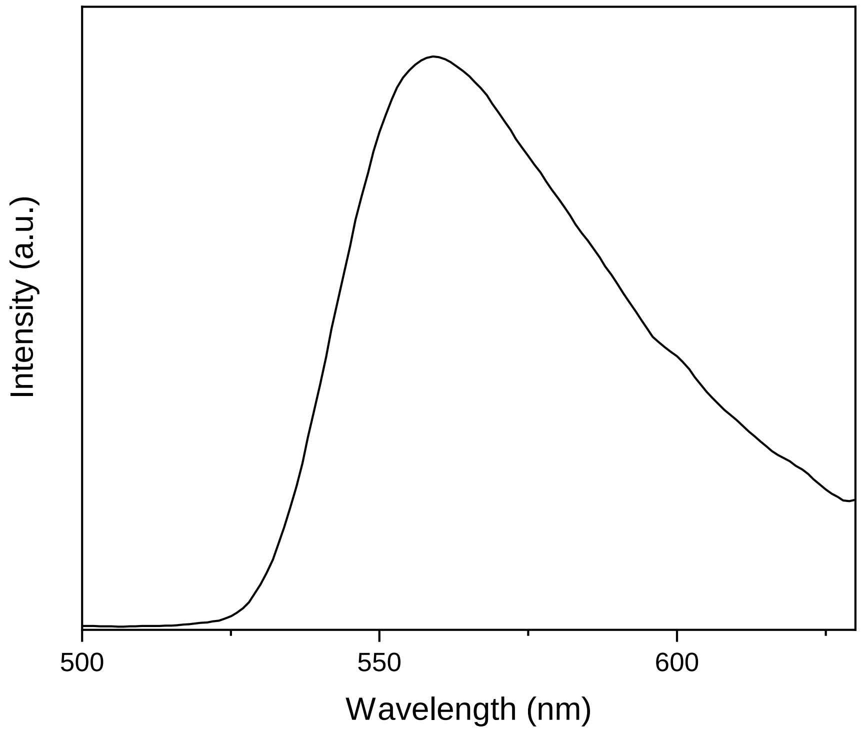

Figure S13. Solid state photoluminescence spectrum of complex **1** under excitation at 326 nm.

Electronic calculations

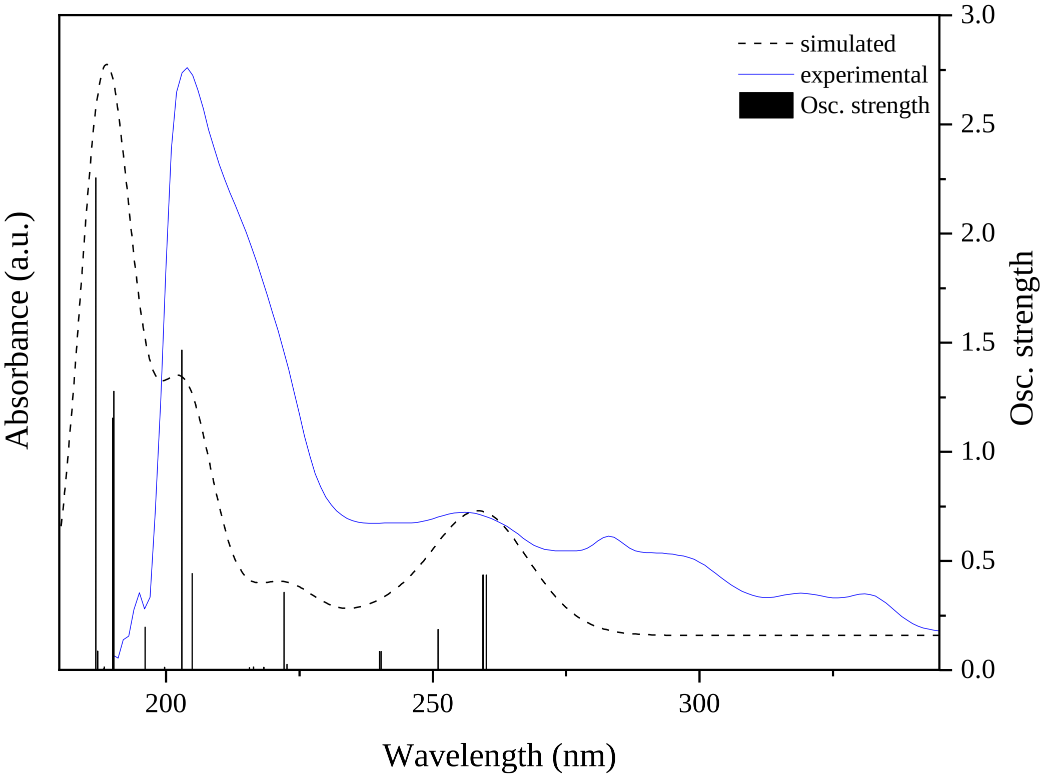

Figure S14. Experimental (blue line), calculated (dashed black line) UV-Vis spectra and oscillator strengths of [Zn(μ-Pip)$_2$(4-acpy)]$_2$ (**1**).

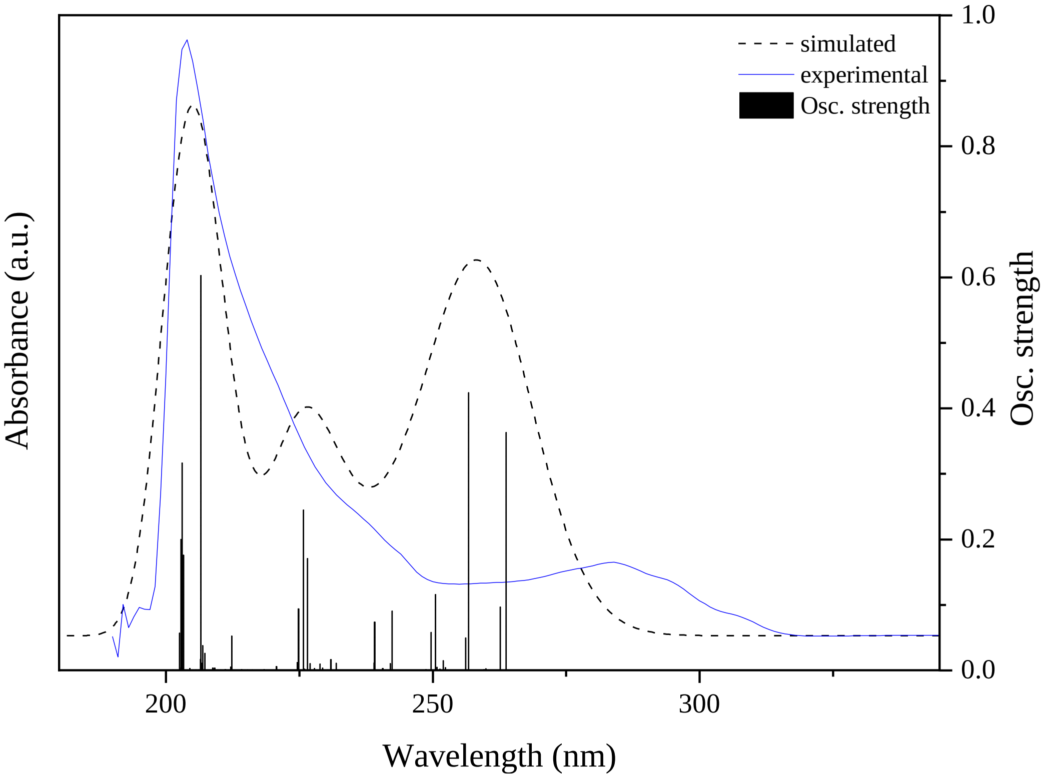

Figure S15. Experimental (blue line), calculated (dashed black line) UV-Vis spectra and oscillator strengths of [Cd(μ-Pip)(Pip)(4-acpy)$_2$]$_2$ (**3**).

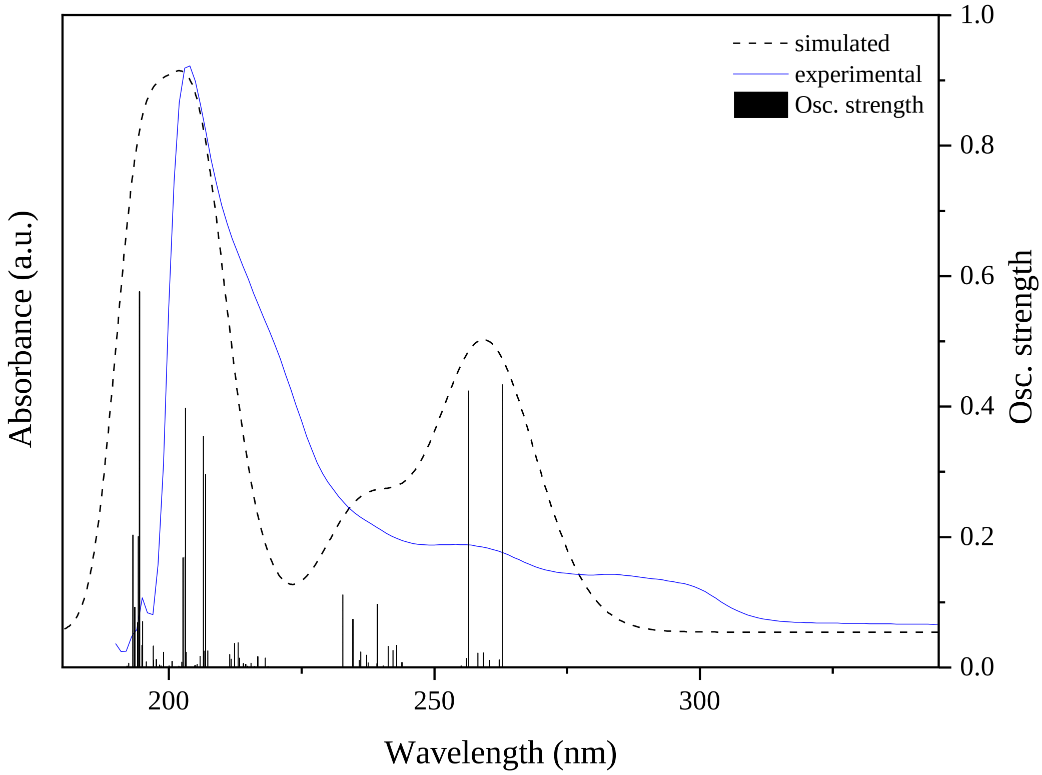

Figure S16. Experimental (blue line), calculated (dashed black line) UV-Vis spectra and oscillator strengths of [Cd(μ-Pip)(Pip)(isn)$_2$]$_2$ (**4**).

HOMO-LUMO gaps

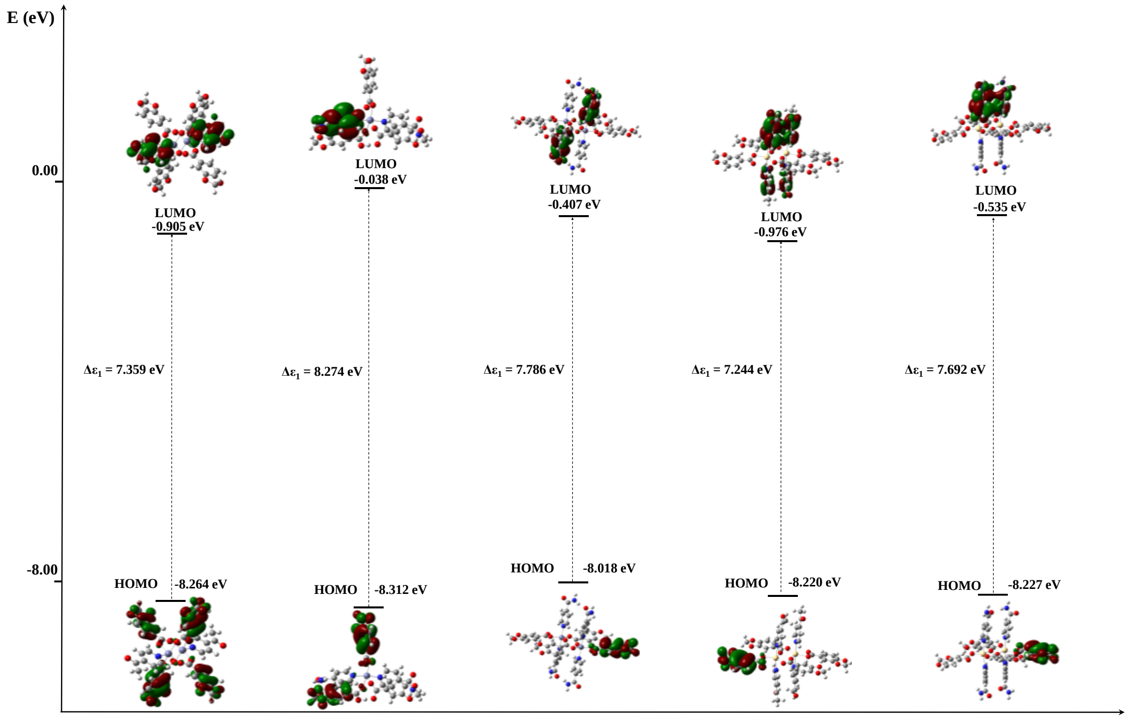

Figure S17. Energy diagram of HOMO-LUMO gaps of complexes **1**-**4**. From left to right, complexes **1**, monomer in **2**, dimer in **2**, **3** and **4**.

MOs representation

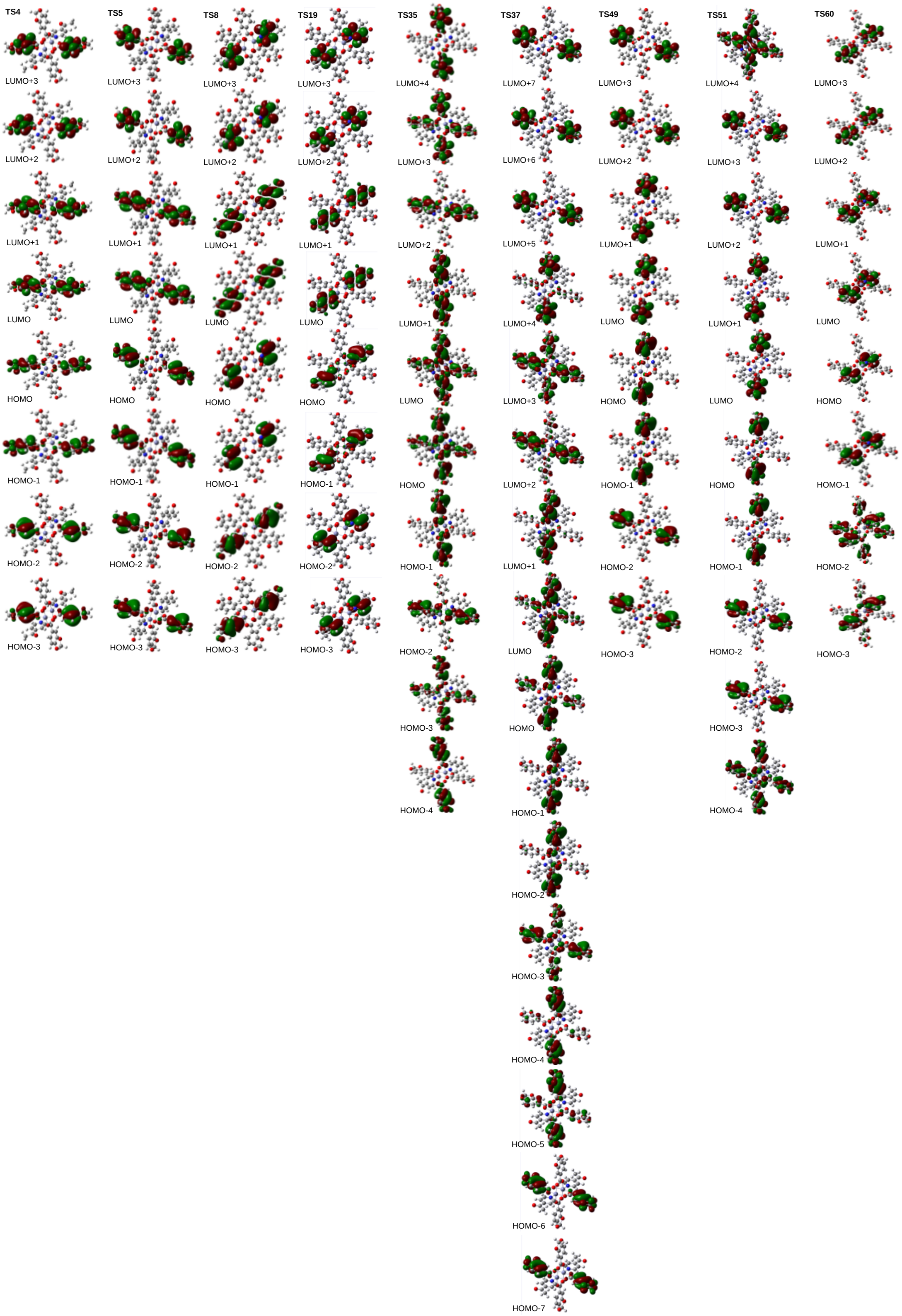

Figure S18. Molecular orbitals representation for the selected electronic transition states (TS) of complex **1**. Oscillator strength values (*f*): TS4, 0.4367; TS5, 0.4366; TS8, 0.1865; TS19, 0.3567; TS35, 0.4426; TS37, 1.4664; TS49, 1.2782; TS51, 1.1540; TS60, 2.2567.

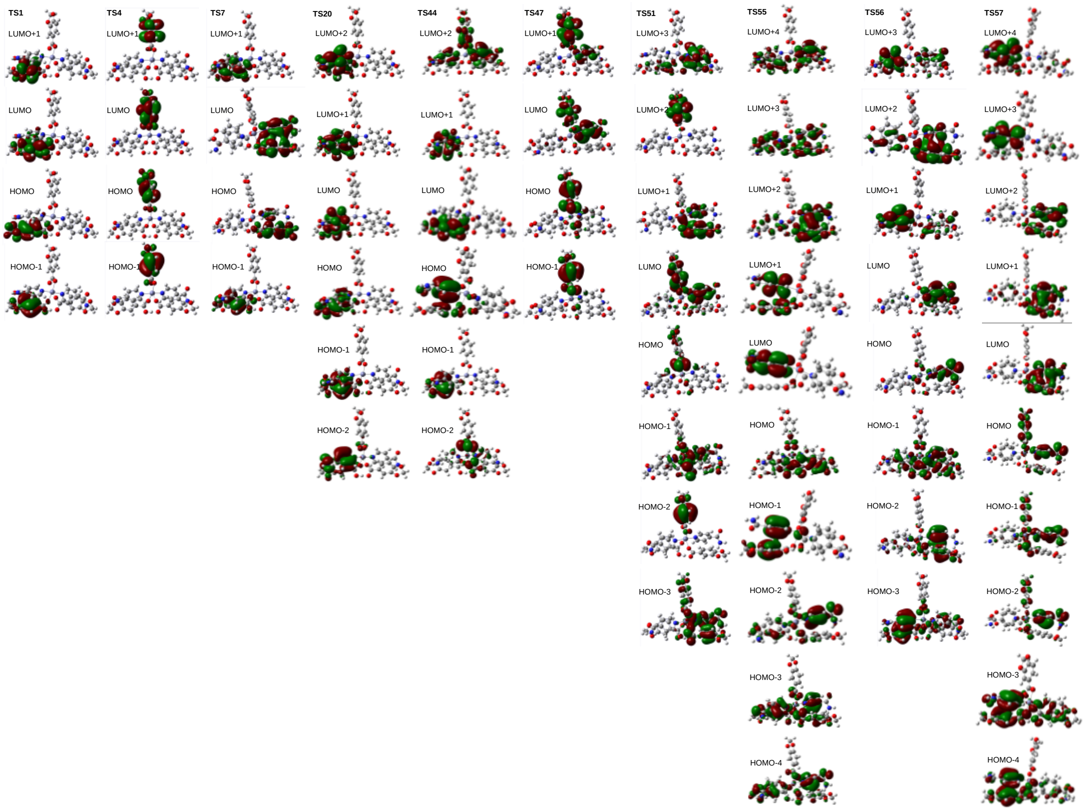

Figure S19. Molecular orbitals representation for the selected electronic transition states (TS) of the monomer in complex **2**. Oscillator strength values (*f*): TS1, 0.2001; TS4, 0.2458; TS7, 0.1148; TS20, 0.1680; TS44, 0.3544; TS47, 0.4349; TS51, 0.2862; TS55, 0.5419; TS56, 0.5041; TS57, 0.3833.

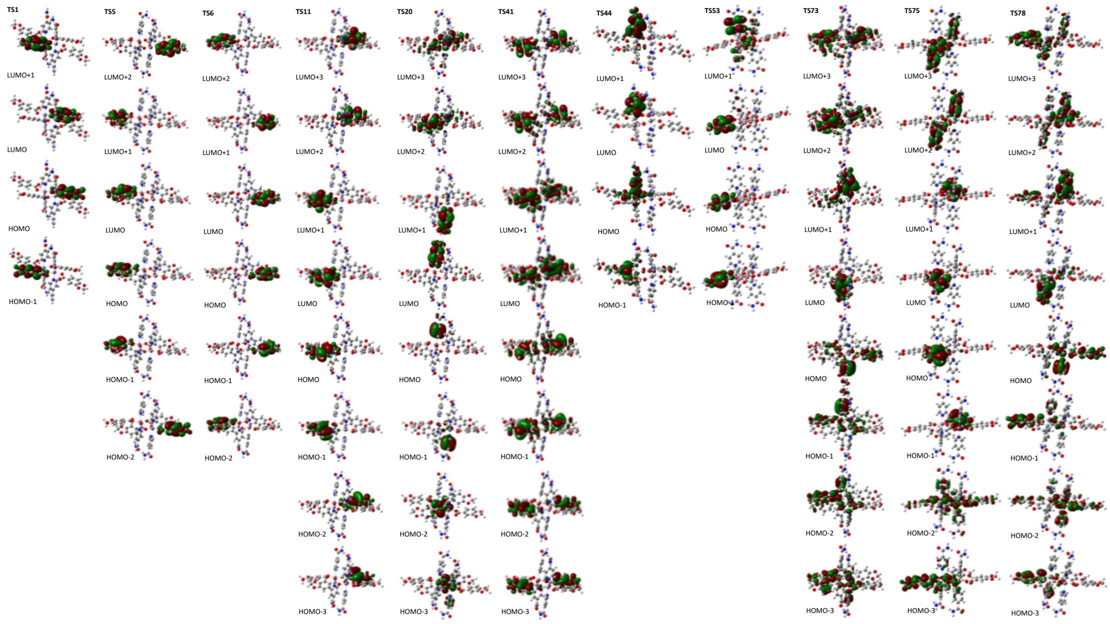

Figure S20. Molecular orbitals representation for the selected electronic transition states (TS) of the dimer in complex **2**. Oscillator strength values (*f*): TS1, 0.4391; TS5, 0.3018; TS6, 0.0894; TS11, 0.0886; TS20, 0.1015; TS41, 0.8745; TS44, 0.1579; TS53, 0.2004; TS73, 0.3385; TS75, 0.4664; TS78, 0.4614.

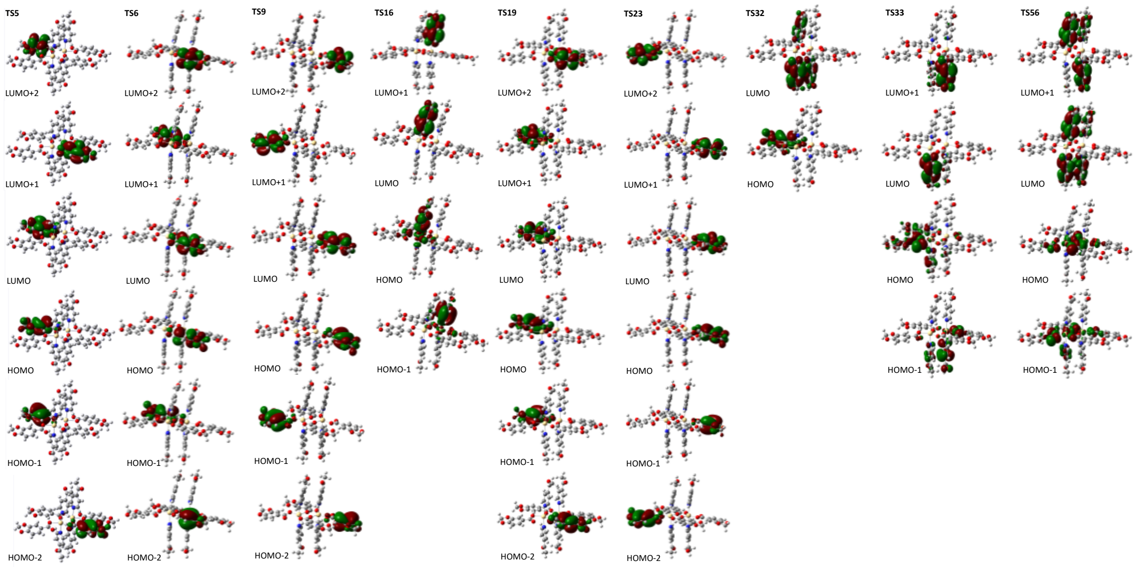

Figure S21. Molecular orbitals representation for the selected electronic transition states (TS) of complex **3**. Oscillator strength values (*f*): TS5, 0.3176; TS6, 0.1106; TS9, 0.4499; TS16, 0.1119; TS19, 0.0951; TS23, 0.0647; TS32, 0.3452; TS33, 0.0680; TS56, 0.0301.

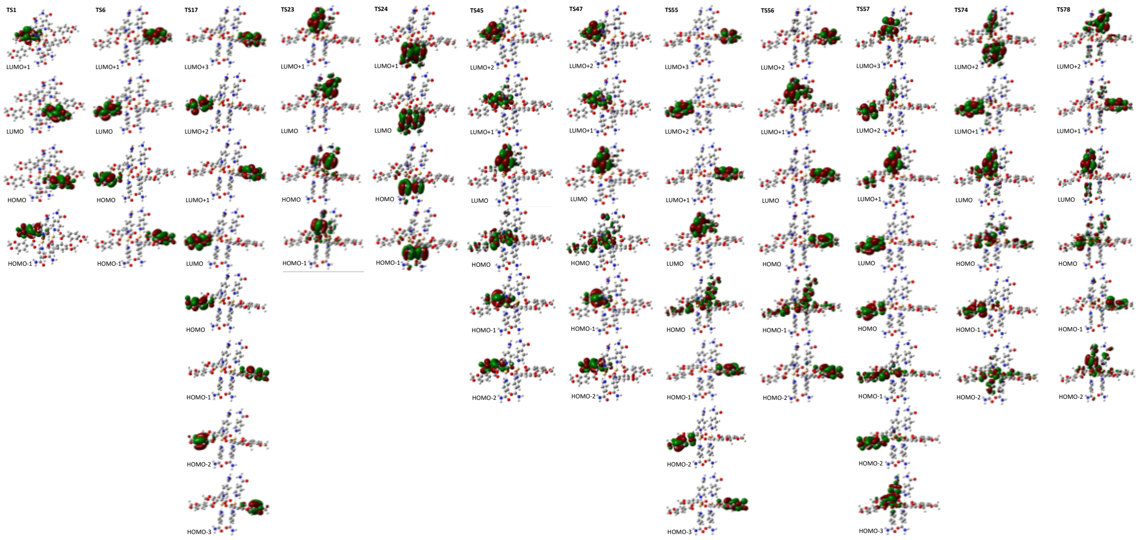

Figure S22. Molecular orbitals representation for the selected electronic transition states (TS) of complex **4**. Oscillator strength values (*f*): TS1, 0.4340; TS6, 0.4243; TS17, 0.0972; TS23, 0.0743; TS24, 0.1117; TS45, 0.2965; TS47, 0.3544; TS55, 0.3980; TS56, 0.1695; TS57, 0.1686; TS74, 0.5762; TS78, 0.2031.

NTOs representation

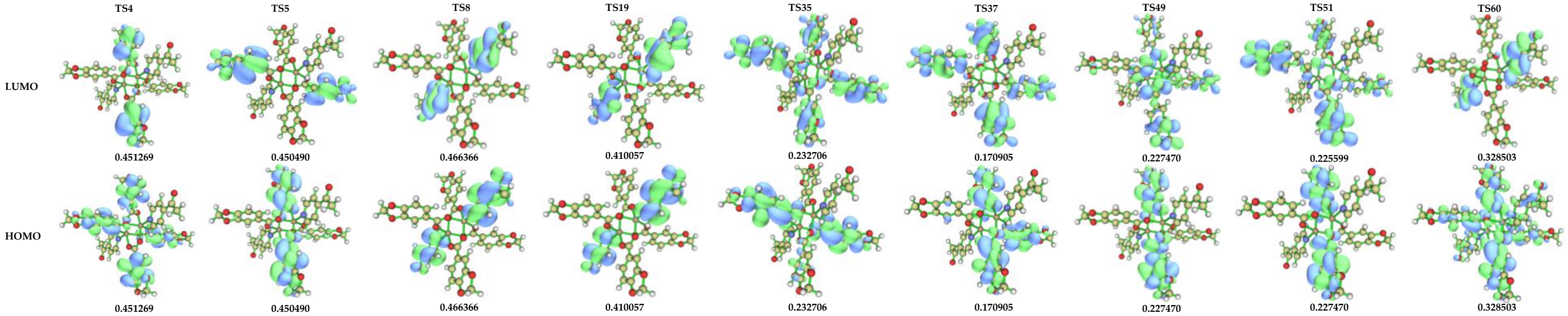

Figure S23. NTOs representation of selected electronic transition states in complex **1**.

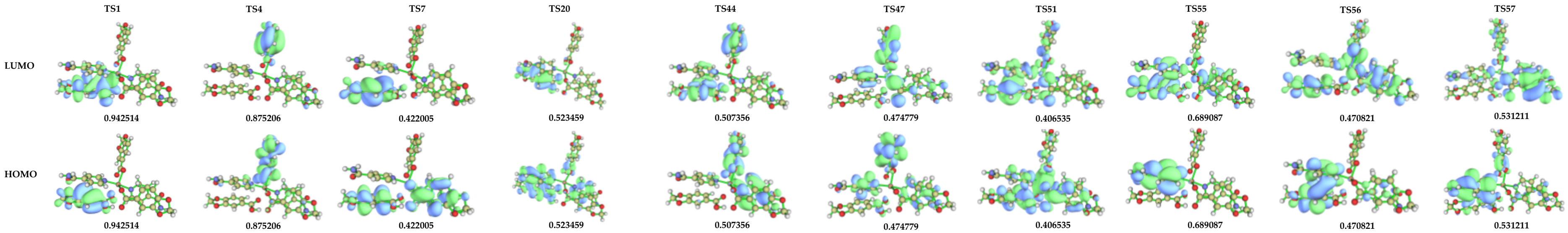

Figure S24. NTOs representation of selected electronic transition states in the monomer of complex **2**.

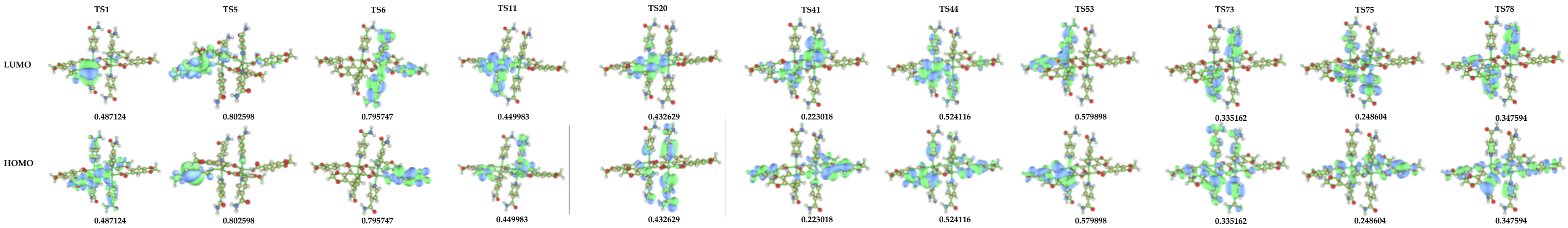

Figure S25. NTOs representation of selected electronic transition states in the dimer of complex **2**.

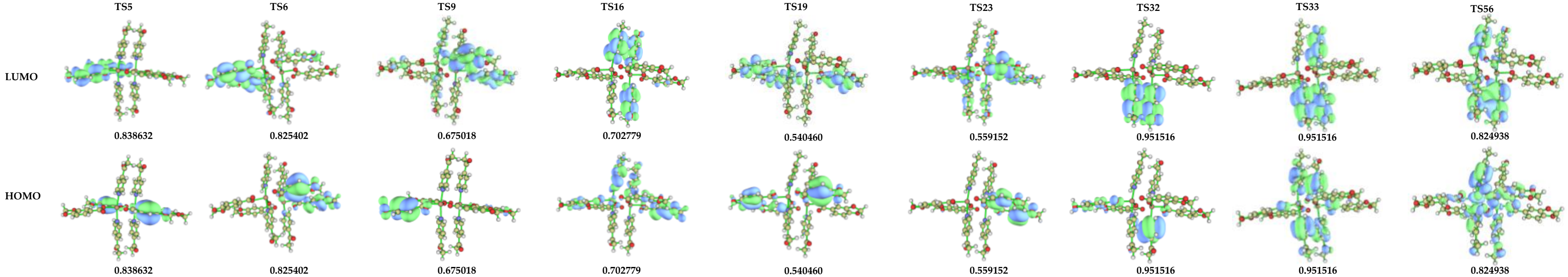

Figure S26. NTOs representation of selected electronic transition states in complex **3**.

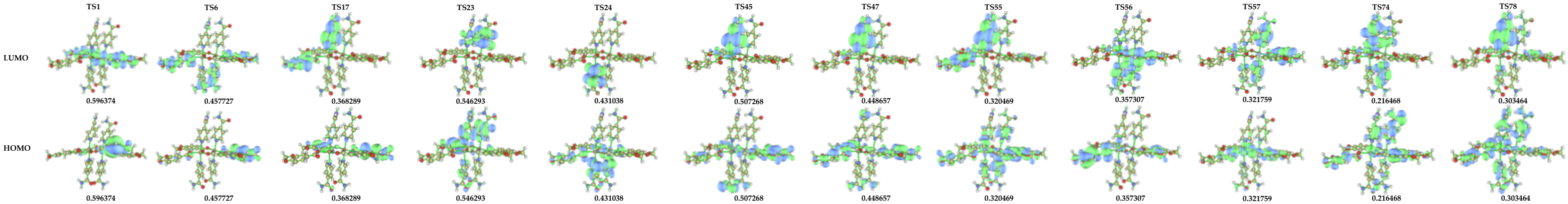

Figure S27. NTOs representation of selected electronic transition states in complex **4**.

Geometry optimization

Table S3. Cartesian coordinates from X-ray and optimized geometry of **1** in MeOH.

Symbol (label)	X-Ray structure			Optimized geometry		
	X	Y	Z	X	Y	Z
Zn (1)	-0.4421000	3.6019000	6.4043000	-1.1262760	-0.2528500	-0.9788810
O (2)	-1.3374000	6.1162000	4.6654000	-0.3957940	1.3764020	1.7965940
O (3)	0.4607000	5.2940000	5.7126000	-2.0914290	1.0044050	0.3390910
O (4)	3.8232000	9.1903000	5.1074000	-5.8944370	3.9940300	2.2266930
O (5)	2.7642000	10.8184000	3.8468000	-5.0951760	4.9758030	4.2010300
O (6)	0.1483000	2.6250000	4.7170000	-1.4437810	-1.7830510	0.3288820
O (7)	-1.6592000	3.4702000	3.7060000	0.2428180	-1.4100690	1.7946380
O (8)	2.6106000	0.4584000	0.7037000	-3.7913160	-5.9637010	2.3132660
O (9)	1.3403000	0.9325000	-1.1887000	-2.6424910	-6.5452040	4.2732810
O (10)	3.6415000	0.5586000	11.1787000	-5.2813320	-1.8228510	-6.3634550
N (11)	0.9437000	2.9806000	7.7569000	-2.5641560	-0.5363930	-2.3627590
C (12)	-0.1378000	6.1859000	5.0556000	-1.5996550	1.5570200	1.3891890
C (13)	0.6230000	7.4267000	4.6951000	-2.4885350	2.4682320	2.1798370
C (14)	1.9437000	7.5940000	5.1462000	-3.8054670	2.7164240	1.7175490
H (15)	2.3812000	6.9336000	5.6707000	-4.1659190	2.2603390	0.8015440
C (16)	2.5697000	8.7682000	4.7861000	-4.5844240	3.5556350	2.4818580
C (17)	3.9958000	10.4686000	4.4674000	-6.3175430	4.7190890	3.4275390
H (18)	4.2467000	11.1535000	5.1369000	-6.7677910	5.6669050	3.1399730
H (19)	4.7159000	10.4154000	3.7896000	-6.9890960	4.0889890	4.0169650
C (20)	1.9392000	9.7460000	4.0233000	-4.1103400	4.1471640	3.6556310
C (21)	0.6572000	9.5935000	3.5601000	-2.8279770	3.9261840	4.1238800
H (22)	0.2364000	10.2559000	3.0250000	-2.4669620	4.3947330	5.0345840
C (23)	0.0035000	8.4106000	3.9177000	-2.0161700	3.0644100	3.3592920
H (24)	-0.8891000	8.2709000	3.6237000	-1.0016490	2.8502910	3.6811510
C (25)	-0.5896000	2.8059000	3.7098000	-0.7793930	-2.0701260	1.3894480
C (26)	-0.1393000	2.2353000	2.3972000	-1.2330350	-3.2483580	2.1969140
C (27)	1.0888000	1.5516000	2.3166000	-2.3607490	-3.9877540	1.7595040
H (28)	1.6123000	1.3722000	3.0890000	-2.8835850	-3.7128540	0.8492750
C (29)	1.4931000	1.1568000	1.0626000	-2.7448110	-5.0541700	2.5410870
C (30)	2.6428000	0.4757000	-0.7349000	-3.8861020	-6.7787620	3.5268250
H (31)	2.8295000	-0.4315000	-1.0848000	-3.9508850	-7.8300810	3.2544480
H (32)	3.3520000	1.0882000	-1.0548000	-4.7341780	-6.4405730	4.1285620
C (33)	0.7330000	1.4102000	-0.0654000	-2.0598800	-5.4072710	3.7065830
C (34)	-0.4803000	2.0513000	-0.0052000	-0.9525930	-4.7068070	4.1493790
H (35)	-1.0012000	2.2065000	-0.7845000	-0.4239060	-4.9928220	5.0538420
C (36)	-0.9123000	2.4649000	1.2607000	-0.5473260	-3.6056910	3.3681640
H (37)	-1.7466000	2.9103000	1.3464000	0.3116660	-3.0136630	3.6698050
C (38)	0.7218000	1.8743000	8.4923000	-2.3465410	-1.3725380	-3.4076240
H (39)	-0.0991000	1.4075000	8.3859000	-1.3885840	-1.8820830	-3.4164980
C (40)	1.6503000	1.3980000	9.3960000	-3.2961840	-1.5520070	-4.4080520
H (41)	1.4635000	0.6233000	9.9132000	-3.1056160	-2.2233280	-5.2387240
C (42)	2.8637000	2.0672000	9.5408000	-4.5088430	-0.8494690	-4.3346470
C (43)	3.0875000	3.1979000	8.7706000	-4.7264900	0.0110750	-3.2484540

H (44)	3.9050000	3.6758000	8.8444000	-5.6450240	0.5782260	-3.1411170
C (45)	2.1074000	3.6212000	7.8948000	-3.7316020	0.1425110	-2.2803410
H (46)	2.2670000	4.3973000	7.3706000	-3.8453280	0.7941330	-1.4200050
C (47)	3.9055000	1.5157000	10.4695000	-5.5322930	-1.0437570	-5.4291050
C (48)	5.2462000	2.1630000	10.4681000	-6.8306590	-0.2870690	-5.3621220
H (49)	5.6255000	2.1271000	9.5658000	-7.3784650	-0.5301120	-4.4420230
H (50)	5.8373000	1.6911000	11.0909000	-7.4467840	-0.5498900	-6.2258930
H (51)	5.1570000	3.0985000	10.7482000	-6.6516130	0.7963200	-5.3649530
Zn (52)	-2.7458000	4.6599000	4.9716000	1.1277240	0.2573510	0.9766940
O (53)	-1.8505000	2.1457000	6.7105000	0.3972700	-1.3718050	-1.7988740
O (54)	-3.6487000	2.9679000	5.6633000	2.0927850	-1.0001570	-0.3411410
O (55)	-7.0112000	-0.9284000	6.2685000	5.8950550	-3.9914570	-2.2275650
O (56)	-5.9522000	-2.5565000	7.5291000	5.0957350	-4.9733970	-4.2018080
O (57)	-3.3363000	5.6369000	6.6589000	1.4453830	1.7874360	-0.3311070
O (58)	-1.5287000	4.7917000	7.6699000	-0.2413870	1.4145920	-1.7967050
O (59)	-5.7986000	7.8035000	10.6722000	3.7932500	5.9677050	-2.3158930
O (60)	-4.5283000	7.3293000	12.5646000	2.6444520	6.5491210	-4.2759610
O (61)	-6.8295000	7.7033000	0.1971000	5.2827350	1.8271820	6.3613830
N (62)	-4.1317000	5.2813000	3.6190000	2.5654970	0.5407280	2.3607260
C (63)	-3.0502000	2.0759000	6.3203000	1.6009970	-1.5527770	-1.3912280
C (64)	-3.8110000	0.8352000	6.6808000	2.4896890	-2.4644480	-2.1815640
C (65)	-5.1317000	0.6679000	6.2297000	3.8064630	-2.7130020	-1.7190230
H (66)	-5.5691000	1.3282000	5.7052000	4.1669400	-2.2568380	-0.8030670
C (67)	-5.7577000	-0.5064000	6.5898000	4.5852340	-3.5526660	-2.4830250
C (68)	-7.1838000	-2.2067000	6.9085000	6.3180530	-4.7171050	-3.4280930
H (69)	-7.4347000	-2.8917000	6.2390000	6.7677170	-5.6650810	-3.1401330
H (70)	-7.9038000	-2.1535000	7.5863000	6.9900900	-4.0875520	-4.0175450
C (71)	-5.1271000	-1.4841000	7.3526000	4.1111160	-4.1442900	-3.6567360
C (72)	-3.8452000	-1.3316000	7.8158000	2.8289050	-3.9229490	-4.1252320
H (73)	-3.4244000	-1.9940000	8.3509000	2.4678630	-4.3915710	-5.0358880
C (74)	-3.1915000	-0.1487000	7.4581000	2.0172900	-3.0607160	-3.3609590
H (75)	-2.2989000	-0.0090000	7.7522000	1.0028920	-2.8463090	-3.6830140
C (76)	-2.5983000	5.4560000	7.6661000	0.7809610	2.0745230	-1.3916510
C (77)	-3.0487000	6.0266000	8.9787000	1.2347080	3.2526290	-2.1992340
C (78)	-4.2767000	6.7103000	9.0593000	2.3625000	3.9919590	-1.7619150
H (79)	-4.8002000	6.8896000	8.2869000	2.8853390	3.7170890	-0.8516780
C (80)	-4.6811000	7.1051000	10.3133000	2.7466480	5.0582660	-2.5436050
C (81)	-5.8308000	7.7862000	12.1108000	3.8880330	6.7827420	-3.5294710
H (82)	-6.0175000	8.6933000	12.4607000	3.9527650	7.8340720	-3.2571150
H (83)	-6.5400000	7.1736000	12.4307000	4.7361410	6.4445800	-4.1311760
C (84)	-3.9210000	6.8517000	11.4413000	2.0617360	5.4113120	-3.7091290
C (85)	-2.7077000	6.2106000	11.3811000	0.9543760	4.7109090	-4.1518410
H (86)	-2.1868000	6.0554000	12.1604000	0.4257090	4.9968820	-5.0563280
C (87)	-2.2757000	5.7970000	10.1152000	0.5490110	3.6099130	-3.3705060
H (88)	-1.4413000	5.3516000	10.0295000	-0.3100550	3.0179460	-3.6720540
C (89)	-3.9097000	6.3876000	2.8836000	2.3482680	1.3775570	3.4051210
H (90)	-3.0889000	6.8543000	2.9900000	1.3906580	1.8877600	3.4135630
C (91)	-4.8382000	6.8638000	1.9799000	3.2978430	1.5568720	4.4056400
H (92)	-4.6515000	7.6386000	1.4627000	3.1075910	2.2287560	5.2359290

C (93)	-6.0517000	6.1947000	1.8350000	4.5100230	0.8534420	4.3328310
C (94)	-6.2755000	5.0640000	2.6053000	4.7272670	-0.0078230	3.2471310
H (95)	-7.0930000	4.5860000	2.5315000	5.6454170	-0.5756840	3.1402640
C (96)	-5.2953000	4.6407000	3.4811000	3.7324740	-0.1390510	2.2788890
H (97)	-5.4549000	3.8646000	4.0053000	3.8459240	-0.7911820	1.4189020
C (98)	-7.0935000	6.7462000	0.9064000	5.5334130	1.0475970	5.4273680
C (99)	-8.4342000	6.0988000	0.9078000	6.8313820	0.2901820	5.3608620
H (100)	-8.8135000	6.1347000	1.8101000	7.3794270	0.5325490	4.4407240
H (101)	-9.0253000	6.5708000	0.2850000	7.4475430	0.5530360	6.2245970
H (102)	-8.3450000	5.1634000	0.6277000	6.6517600	-0.7931100	5.3641290

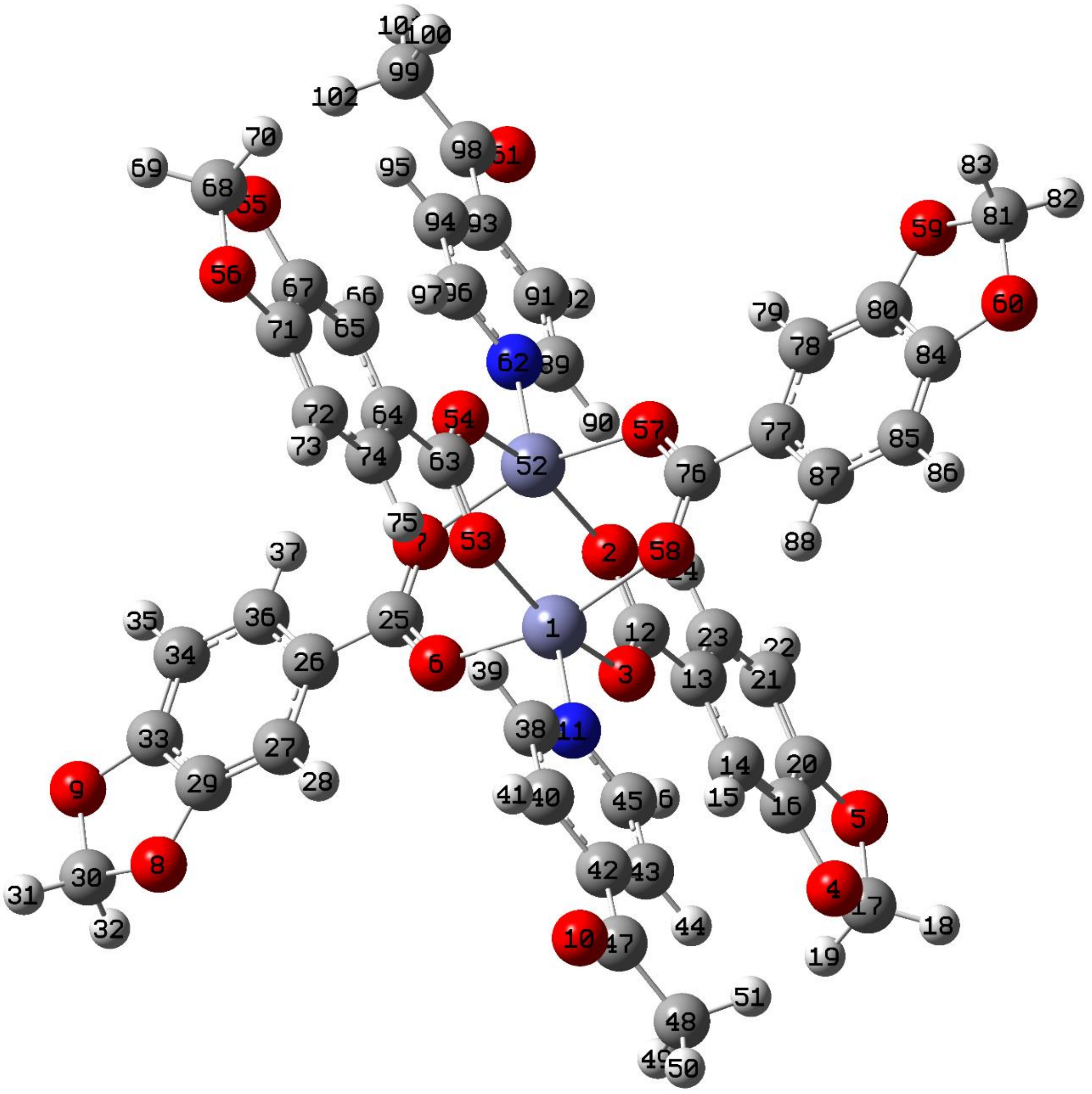

Figure S28. Optimized geometry of **1** in MeOH solution with labelling scheme.

Table S4. Cartesian coordinates from X-ray and optimized geometry of the monomer present in **2** in MeOH.

Symbol (label)	X-Ray structure			Optimized geometry		
	X	Y	Z	X	Y	Z

Zn (1)	7.7312000	14.6545000	6.9711000	0.0771250	0.0147330	0.2007710
O (2)	7.5770000	14.7442000	8.9646000	0.8089610	-1.4228870	1.5087800
O (3)	5.3506000	14.8097000	9.2492000	1.1755130	-3.4292830	0.5352630
O (4)	7.1084000	14.1041000	15.2042000	7.1627870	-1.7507440	1.5848470
O (5)	5.0107000	14.6628000	14.3928000	6.3631440	-3.3364500	0.0582120
O (6)	6.0632000	15.1296000	5.8745000	-0.6355060	-1.4244810	-1.2753070
O (7)	4.3840000	15.8267000	7.1760000	-1.1149780	-3.4299050	-0.3509050
H (8)	4.9170000	15.6168000	7.7903000	-0.1060100	-3.4244900	0.0473110
O (9)	1.8336000	16.9276000	1.6169000	-6.9212380	-1.8970890	-2.2036380
O (10)	0.8134000	17.0454000	3.6959000	-6.2844960	-3.5865830	-0.7056090
O (11)	9.8183000	14.0147000	7.0509000	0.4649020	1.8779360	1.1852070
O (12)	8.9059000	14.2925000	5.0783000	-0.5208410	2.0219500	-0.8086450
O (13)	14.6787000	12.6672000	3.1789000	-0.2522600	8.2172360	0.5271250
O (14)	12.8615000	13.2838000	1.8686000	-1.1200400	7.2000810	-1.3999940
O (15)	9.3681000	21.4222000	5.5431000	-6.8274910	-0.8325250	1.1038290
O (16)	7.2790000	7.6443000	7.4536000	6.5283210	0.8290500	-2.6310900
N (17)	8.3251000	16.7075000	6.8610000	-1.8620630	-0.1548320	0.9752720
N (18)	10.3983000	21.3079000	7.5475000	-6.2534030	-1.3802630	3.2478820
H (19)	10.6843000	22.1383000	7.4916000	-7.2100730	-1.6096190	3.4757670
H (20)	10.5924000	20.8224000	8.2553000	-5.5736720	-1.4053080	3.9918960
N (21)	7.1049000	12.5938000	6.9264000	1.9329960	0.0149890	-0.7722820
N (22)	5.9090000	7.7904000	5.6793000	6.5647350	-1.4337290	-2.2784740
H (23)	5.8611000	6.9151000	5.6007000	7.5009420	-1.4993360	-2.6529430
H (24)	5.4690000	8.3071000	5.1190000	6.1873060	-2.2519730	-1.8152580
C (25)	6.5380000	14.7009000	9.6786000	1.5924740	-2.3811240	1.1505520
C (26)	6.7285000	14.5169000	11.1465000	3.0585450	-2.2431960	1.3899030
C (27)	7.9877000	14.1798000	11.6341000	3.5421370	-1.2747120	2.2839870
H (28)	8.7070000	14.0701000	11.0229000	2.8273530	-0.6741290	2.8391390
C (29)	8.2245000	13.9999000	13.0066000	4.9229320	-1.0551860	2.4524840
H (30)	9.0787000	13.7569000	13.3435000	5.3040860	-0.3030440	3.1362810
C (31)	7.1419000	14.1946000	13.8300000	5.7704820	-1.8259470	1.6766250
C (32)	5.7614000	14.3513000	15.5639000	7.5752360	-2.8884750	0.7541730
H (33)	5.7161000	15.1059000	16.2019000	7.9414510	-3.6920500	1.3980110
H (34)	5.3793000	13.5499000	16.0031000	8.3087040	-2.5559050	0.0234570
C (35)	5.8921000	14.5274000	13.3527000	5.2887710	-2.7875230	0.7865650
C (36)	5.6415000	14.6881000	12.0160000	3.9467880	-3.0334760	0.6202880
H (37)	4.7736000	14.9030000	11.6949000	3.5718170	-3.7656310	-0.0873170
C (38)	4.9487000	15.6250000	6.0251000	-1.4596380	-2.3572700	-1.0284780
C (39)	4.1482000	16.0154000	4.8470000	-2.8798140	-2.2591860	-1.4281390
C (40)	4.7585000	15.9532000	3.5973000	-3.2690930	-1.2512930	-2.3256430
H (41)	5.6717000	15.6962000	3.5387000	-2.5060630	-0.6043110	-2.7490930
C (42)	4.0576000	16.2585000	2.4329000	-4.6208650	-1.0554420	-2.6627420
H (43)	4.4675000	16.2231000	1.5762000	-4.9309690	-0.2824580	-3.3590280
C (44)	2.7387000	16.6142000	2.5948000	-5.5400020	-1.8843300	-2.0439390
C (45)	0.6247000	17.2524000	2.2948000	-7.4471910	-2.8413690	-1.2031750
H (46)	-0.1110000	16.6782000	1.9654000	-8.1352930	-3.5296820	-1.6898660
H (47)	0.3842000	18.1968000	2.1229000	-7.8852930	-2.2748050	-0.3809150
C (48)	2.1254000	16.6937000	3.8292000	-5.1548020	-2.8892060	-1.1499190
C (49)	2.8017000	16.4050000	4.9936000	-3.8378710	-3.1175930	-0.8277840

H (50)	2.3847000	16.4652000	5.8449000	-3.5375700	-3.8945910	-0.1326300
C (51)	9.9162000	14.0168000	5.7864000	-0.0440520	2.6005230	0.2362960
C (52)	11.2122000	13.6671000	5.1505000	-0.0680730	4.0835990	0.3619010
C (53)	12.3155000	13.3094000	5.9211000	0.4451470	4.6999930	1.5141680
H (54)	12.2324000	13.3066000	6.8672000	0.8555650	4.0738570	2.3008580
C (55)	13.5349000	12.9549000	5.3499000	0.4354660	6.1011600	1.6629930
H (56)	14.2822000	12.7070000	5.8825000	0.8250780	6.5854310	2.5534180
C (57)	13.6030000	12.9809000	3.9758000	-0.0968470	6.8316410	0.6161940
C (58)	14.2492000	12.8267000	1.8276000	-0.7040340	8.4904140	-0.8441610
H (59)	14.3122000	11.9657000	1.3428000	0.1334560	8.8855720	-1.4252050
H (60)	14.8159000	13.4920000	1.3628000	-1.5569310	9.1652970	-0.8146970
C (61)	12.5280000	13.3534000	3.2052000	-0.6083060	6.2215050	-0.5325320
C (62)	11.3164000	13.7092000	3.7422000	-0.6155240	4.8545070	-0.6948530
H (63)	10.5857000	13.9706000	3.1945000	-1.0179790	4.3742830	-1.5811580
C (64)	8.8876000	17.3024000	7.9021000	-2.1452470	-1.0905670	1.9141720
H (65)	8.9767000	16.8085000	8.7097000	-1.2917480	-1.6240660	2.3177510
C (66)	9.3535000	18.6092000	7.8749000	-3.4534250	-1.3777640	2.3009420
H (67)	9.7562000	18.9934000	8.6451000	-3.6279620	-2.1671190	3.0263810
C (68)	9.2234000	19.3521000	6.6985000	-4.5081720	-0.6764020	1.6999700
C (69)	8.6094000	18.7380000	5.6210000	-4.2107790	0.3040980	0.7465050
H (70)	8.4806000	19.2133000	4.8085000	-5.0076700	0.8520270	0.2547040
C (71)	8.1872000	17.4287000	5.7404000	-2.8808090	0.5421650	0.4123730
H (72)	7.7772000	17.0174000	4.9879000	-2.5954700	1.2795820	-0.3299420
C (73)	9.6665000	20.7823000	6.5553000	-5.9557610	-0.9709740	1.9982300
C (74)	7.5115000	11.7603000	7.8919000	2.9011930	0.8397670	-0.2988520
H (75)	7.9382000	12.1290000	8.6558000	2.5924810	1.5421230	0.4678090
C (76)	7.3424000	10.3921000	7.8340000	4.2140790	0.7803280	-0.7557460
H (77)	7.6531000	9.8399000	8.5413000	4.9607000	1.4622660	-0.3612320
C (78)	6.7122000	9.8310000	6.7309000	4.5548610	-0.1787350	-1.7173750
C (79)	6.2280000	10.6958000	5.7608000	3.5555240	-1.0280600	-2.2109200
H (80)	5.7483000	10.3560000	5.0147000	3.7742410	-1.7772240	-2.9662730
C (81)	6.4466000	12.0552000	5.8887000	2.2549130	-0.8938010	-1.7247090
H (82)	6.1159000	12.6341000	5.2117000	1.4421970	-1.5229120	-2.0691890
C (83)	6.6384000	8.3265000	6.6354000	5.9673400	-0.2242990	-2.2414460

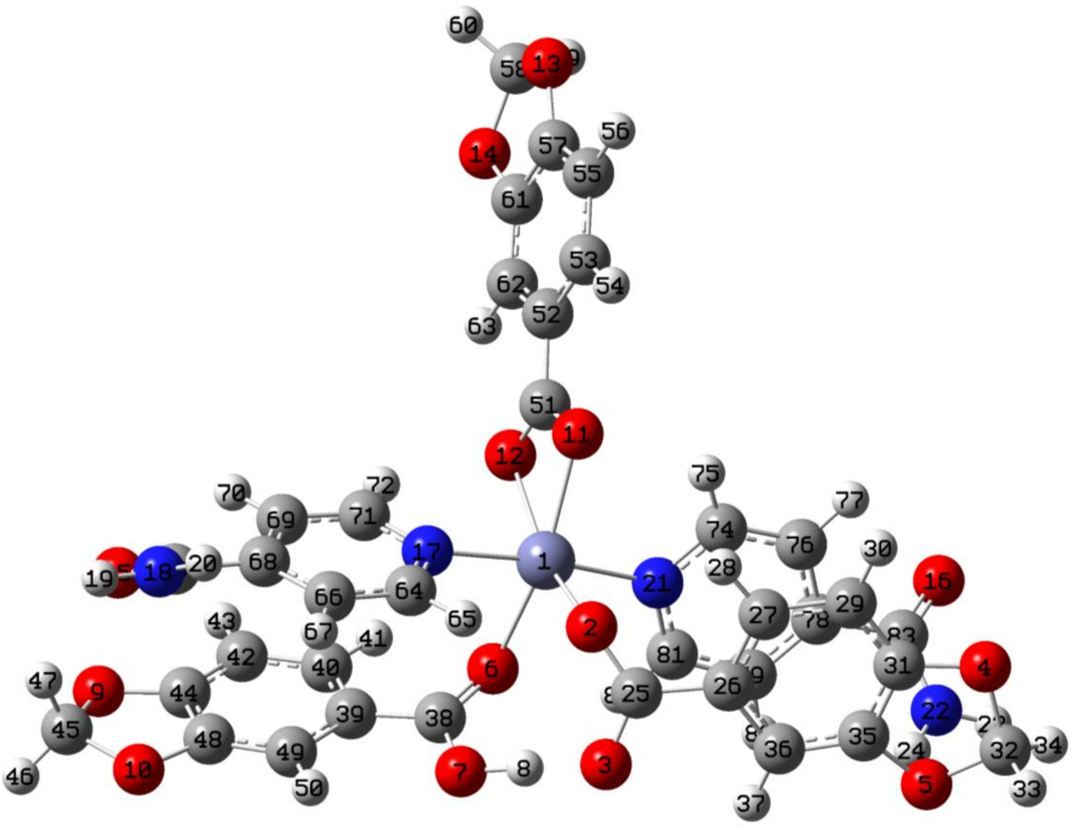

Figure S29. Optimized geometry of the monomer present in **2** in MeOH solution with labelling scheme.

Table S5. Cartesian coordinates from X-ray and optimized geometry of the dimer present in **2** in MeOH.

Symbol (label)	X-Ray structure			Optimized geometry		
	X	Y	Z	X	Y	Z
Zn (1)	5.8999000	8.7748000	1.3491000	-1.7428240	0.8767620	-0.6238630
O (2)	3.8696000	9.4500000	1.3978000	-3.7121540	1.0392820	-0.2826040
O (3)	4.7969000	8.9633000	3.3254000	-4.1652680	1.4683370	-2.4560800
O (4)	-0.9940000	10.4907000	5.2749000	-10.0811050	0.5966630	-0.3471640
O (5)	0.8089000	9.7952000	6.5519000	-9.4077170	1.3384270	-2.4673060
O (6)	7.5185000	8.2741000	2.4588000	-0.3212580	1.0714470	-2.1478250
O (7)	8.7071000	7.7003000	0.6833000	1.0622330	-0.3700760	-1.1169280
O (8)	12.7421000	6.0932000	5.1774000	5.2003000	1.8896340	-5.2816250
O (9)	13.1119000	6.1280000	2.8830000	5.5582740	0.2945640	-3.5983730
O (10)	4.9094000	1.8324000	2.2303000	-1.3383370	7.9393470	-0.6290500
O (11)	7.3329000	15.6388000	0.5121000	-2.5681080	-6.0695610	-1.9722010
N (12)	5.3287000	6.6997000	1.3068000	-1.3944150	2.9244160	-0.3339250
N (13)	3.7604000	1.9456000	0.3001000	0.5327560	7.5215760	0.6236170
H (14)	3.6123000	1.0783000	0.2840000	0.7157380	8.5124810	0.6962280
H (15)	3.4505000	2.4567000	-0.3459000	1.2190540	6.8853020	1.0327220
N (16)	6.5852000	10.8130000	1.3726000	-2.0929800	-1.1098800	-1.3035980

N (17)	8.2603000	15.4979000	2.5572000	-3.5559260	-5.2422890	-3.8545500
H (18)	8.4639000	16.3541000	2.5671000	-3.7304400	-6.1733610	-4.2058020
H (19)	8.4658000	14.9890000	3.2450000	-3.9171020	-4.4627960	-4.3832110
C (20)	3.7885000	9.3295000	2.6528000	-4.5392990	1.1955450	-1.2765050
C (21)	2.4998000	9.6262000	3.3331000	-6.0013970	1.0173200	-0.9691200
C (22)	1.4065000	10.0202000	2.5607000	-6.4136450	0.5787520	0.2988850
H (23)	1.5086000	10.0705000	1.6183000	-5.6640230	0.3767780	1.0568700
C (24)	0.1608000	10.3461000	3.1234000	-7.7768280	0.3940990	0.6078960
H (25)	-0.5810000	10.6197000	2.5982000	-8.0971540	0.0592030	1.5902650
C (26)	0.1003000	10.2430000	4.4549000	-8.6832750	0.6622610	-0.4012800
C (27)	-0.5509000	10.2917000	6.5826000	-10.5479270	0.7961630	-1.7248530
H (28)	-1.1366000	9.6396000	7.0420000	-11.3613120	1.5187910	-1.7290200
H (29)	-0.5853000	11.1462000	7.0834000	-10.8341460	-0.1699280	-2.1504200
C (30)	1.1505000	9.8370000	5.2459000	-8.2779770	1.0992480	-1.6646940
C (31)	2.4013000	9.5217000	4.7208000	-6.9517750	1.2909800	-1.9842680
H (32)	3.1327000	9.2569000	5.2653000	-6.6303720	1.6342190	-2.9628920
C (33)	8.5407000	7.7773000	1.9197000	0.8247340	0.5132540	-2.0176890
C (34)	9.6142000	7.2723000	2.8233000	1.9369020	0.8936390	-2.9405810
C (35)	9.3941000	7.2109000	4.1872000	1.7301200	1.8513430	-3.9452490
H (36)	8.5402000	7.4483000	4.5300000	0.7429590	2.2916830	-4.0524790
C (37)	10.4034000	6.8062000	5.0806000	2.7755790	2.2496820	-4.8022450
H (38)	10.2572000	6.7585000	6.0174000	2.6280060	2.9944620	-5.5784780
C (39)	11.5991000	6.4853000	4.5179000	4.0051790	1.6478890	-4.6016900
C (40)	13.7284000	5.8958000	4.1582000	6.1659490	0.8970410	-4.7882220
H (41)	14.0794000	4.9704000	4.2006000	7.0890940	1.4008610	-4.5084150
H (42)	14.4828000	6.5222000	4.2892000	6.3128440	0.1293670	-5.5518800
C (43)	11.8271000	6.5186000	3.1540000	4.2125700	0.6952890	-3.6002200
C (44)	10.8476000	6.9048000	2.2556000	3.2092560	0.2928430	-2.7496220
H (45)	10.9995000	6.9215000	1.3179000	3.3740330	-0.4252500	-1.9489700
C (46)	5.7329000	5.8794000	2.2863000	-1.7611200	3.7767970	-1.3218030
H (47)	6.2392000	6.2411000	3.0039000	-2.2780250	3.3349790	-2.1686610
C (48)	5.4423000	4.5270000	2.2965000	-1.4978730	5.1415080	-1.2493360
H (49)	5.7468000	3.9804000	3.0115000	-1.8064440	5.8084770	-2.0477370
C (50)	4.7069000	3.9718000	1.2616000	-0.8305730	5.6481440	-0.1249110
C (51)	4.2663000	4.8331000	0.2626000	-0.4741960	4.7659250	0.9035900
H (52)	3.7388000	4.5018000	-0.4556000	0.0164830	5.1164240	1.8065260
C (53)	4.6021000	6.1782000	0.3205000	-0.7730900	3.4100720	0.7627620
H (54)	4.3003000	6.7533000	-0.3726000	-0.5211180	2.6784220	1.5242650
C (55)	4.4539000	2.4857000	1.3042000	-0.5597200	7.1312740	-0.0576280
C (56)	7.3020000	11.2856000	2.3988000	-1.9119890	-1.4261220	-2.6055260
H (57)	7.5580000	10.6821000	3.0854000	-1.5302980	-0.6348700	-3.2401150
C (58)	7.6906000	12.6206000	2.5079000	-2.1897160	-2.6985110	-3.1056660
H (59)	8.2047000	12.9194000	3.2498000	-2.0180710	-2.9097690	-4.1570880
C (60)	7.3063000	13.5017000	1.5071000	-2.6547490	-3.6821440	-2.2233270
C (61)	6.5794000	13.0136000	0.4330000	-2.8314440	-3.3570910	-0.8721270
H (62)	6.3145000	13.5920000	-0.2725000	-3.1750100	-4.1018620	-0.1614140
C (63)	6.2445000	11.6734000	0.4006000	-2.5568080	-2.0572090	-0.4551740
H (64)	5.7500000	11.3456000	-0.3422000	-2.7122530	-1.7546900	0.5740960
C (65)	7.6441000	14.9737000	1.5037000	-2.9260350	-5.0895230	-2.6786570

Zn (66)	8.6601000	7.7669000	-1.3491000	1.7356130	-0.8343720	0.6971640
O (67)	10.6904000	7.0916000	-1.3978000	3.7003510	-1.0251190	0.3749840
O (68)	9.7631000	7.5784000	-3.3254000	4.2770010	-1.5086560	2.5121350
O (69)	15.5540000	6.0510000	-5.2749000	10.0689020	-0.7848200	0.0368740
O (70)	13.7511000	6.7465000	-6.5519000	9.5091250	-1.5730360	2.1731780
O (71)	7.0415000	8.2676000	-2.4588000	0.3275300	-1.0156890	2.2358190
O (72)	5.8530000	8.8413000	-0.6833000	-1.0536260	0.4112320	1.1842130
O (73)	1.8179000	10.4485000	-5.1774000	-5.2268290	-1.8850060	5.2976660
O (74)	1.4481000	10.4136000	-2.8830000	-5.5851940	-0.3170780	3.5888810
O (75)	9.6506000	14.7093000	-2.2303000	1.2887940	-7.8945910	0.7052680
O (76)	7.2271000	0.9028000	-0.5121000	2.5837500	6.1227290	1.9633860
N (77)	9.2313000	9.8420000	-1.3068000	1.3650340	-2.8801810	0.4073030
N (78)	10.7996000	14.5960000	-0.3001000	-0.5513580	-7.4734060	-0.5915070
H (79)	10.9477000	15.4634000	-0.2840000	-0.7352830	-8.4638830	-0.6675090
H (80)	11.1095000	14.0849000	0.3459000	-1.2262970	-6.8352990	-1.0165730
N (81)	7.9748000	5.7287000	-1.3726000	2.1019590	1.1566780	1.3609560
N (82)	6.2997000	1.0437000	-2.5572000	3.5645710	5.3216450	3.8610170
H (83)	6.0961000	0.1875000	-2.5671000	3.7382040	6.2574080	4.2000460
H (84)	6.0942000	1.5527000	-3.2450000	3.9223870	4.5492830	4.4023080
C (85)	10.7715000	7.2122000	-2.6528000	4.5788720	-1.2224160	1.3160120
C (86)	12.0602000	6.9154000	-3.3331000	6.0251790	-1.0814530	0.9185930
C (87)	13.1535000	6.5214000	-2.5607000	6.3701030	-0.6156680	-0.3595090
H (88)	13.0514000	6.4711000	-1.6183000	5.5806770	-0.3630500	-1.0589270
C (89)	14.3992000	6.1956000	-3.1234000	7.7154380	-0.4681580	-0.7541710
H (90)	15.1410000	5.9220000	-2.5982000	7.9798490	-0.1128530	-1.7459110
C (91)	14.4597000	6.2986000	-4.4549000	8.6759320	-0.8016150	0.1828590
C (92)	15.1110000	6.2500000	-6.5826000	10.6172950	-1.0462620	1.3734150
H (93)	15.6966000	6.9020000	-7.0420000	11.4018600	-1.7966010	1.3008290
H (94)	15.1453000	5.3955000	-7.0834000	10.9664560	-0.1054920	1.8087190
C (95)	13.4096000	6.7047000	-5.2459000	8.3385300	-1.2660460	1.4563430
C (96)	12.1587000	7.0200000	-4.7208000	7.0299710	-1.4220760	1.8582250
H (97)	11.4273000	7.2847000	-5.2653000	6.7620990	-1.7871950	2.8448890
C (98)	6.0193000	8.7644000	-1.9197000	-0.8220800	-0.4677800	2.0907020
C (99)	4.9458000	9.2693000	-2.8233000	-1.9429750	-0.8564950	2.9993280
C (100)	5.1659000	9.3307000	-4.1872000	-1.7359950	-1.7988940	4.0185270
H (101)	6.0198000	9.0933000	-4.5300000	-0.7431730	-2.2204550	4.1469880
C (102)	4.1566000	9.7354000	-5.0806000	-2.7880610	-2.2059730	4.8632110
H (103)	4.3028000	9.7831000	-6.0174000	-2.6398820	-2.9388490	5.6505890
C (104)	2.9610000	10.0563000	-4.5179000	-4.0248680	-1.6290350	4.6353210
C (105)	0.8317000	10.6459000	-4.1582000	-6.2023520	-0.9171800	4.7750130
H (106)	0.4806000	11.5713000	-4.2006000	-7.1104390	-1.4422710	4.4852560
H (107)	0.0772000	10.0195000	-4.2892000	-6.3776060	-0.1435940	5.5266350
C (108)	2.7330000	10.0231000	-3.1540000	-4.2323720	-0.6924340	3.6190480
C (109)	3.7124000	9.6369000	-2.2556000	-3.2228070	-0.2811610	2.7803370
H (110)	3.5605000	9.6201000	-1.3179000	-3.3911850	0.4233250	1.9681480
C (111)	8.8271000	10.6622000	-2.2863000	1.7015560	-3.7325360	1.4060970
H (112)	8.3208000	10.3005000	-3.0039000	2.1958160	-3.2925620	2.2670160
C (113)	9.1177000	12.0147000	-2.2965000	1.4365220	-5.0964500	1.3286180
H (114)	8.8132000	12.5613000	-3.0115000	1.7215040	-5.7630190	2.1360580

C (115)	9.8531000	12.5699000	-1.2616000	0.7983810	-5.6027870	0.1872370
C (116)	10.2938000	11.7085000	-0.2626000	0.4719540	-4.7210000	-0.8514190
H (117)	10.8212000	12.0399000	0.4556000	0.0039020	-5.0713350	-1.7663800
C (118)	9.9580000	10.3634000	-0.3205000	0.7714500	-3.3657400	-0.7047040
H (119)	10.2597000	9.7884000	0.3726000	0.5416470	-2.6345230	-1.4736200
C (120)	10.1062000	14.0560000	-1.3042000	0.5257130	-7.0853100	0.1148630
C (121)	7.2581000	5.2561000	-2.3988000	1.9125230	1.4922410	2.6569090
H (122)	7.0020000	5.8595000	-3.0854000	1.5200050	0.7126180	3.2994360
C (123)	6.8695000	3.9210000	-2.5079000	2.1930980	2.7701470	3.1413460
H (124)	6.3553000	3.6223000	-3.2498000	2.0146300	2.9971690	4.1883590
C (125)	7.2537000	3.0399000	-1.5071000	2.6682980	3.7393390	2.2485260
C (126)	7.9806000	3.5281000	-0.4330000	2.8550900	3.3939940	0.9037250
H (127)	8.2455000	2.9497000	0.2725000	3.2077110	4.1269610	0.1852410
C (128)	8.3156000	4.8683000	-0.4006000	2.5787630	2.0892550	0.5033220
H (129)	8.8100000	5.1960000	0.3422000	2.7422630	1.7708980	-0.5199560
C (130)	6.9159000	1.5680000	-1.5037000	2.9389790	5.1527870	2.6851170

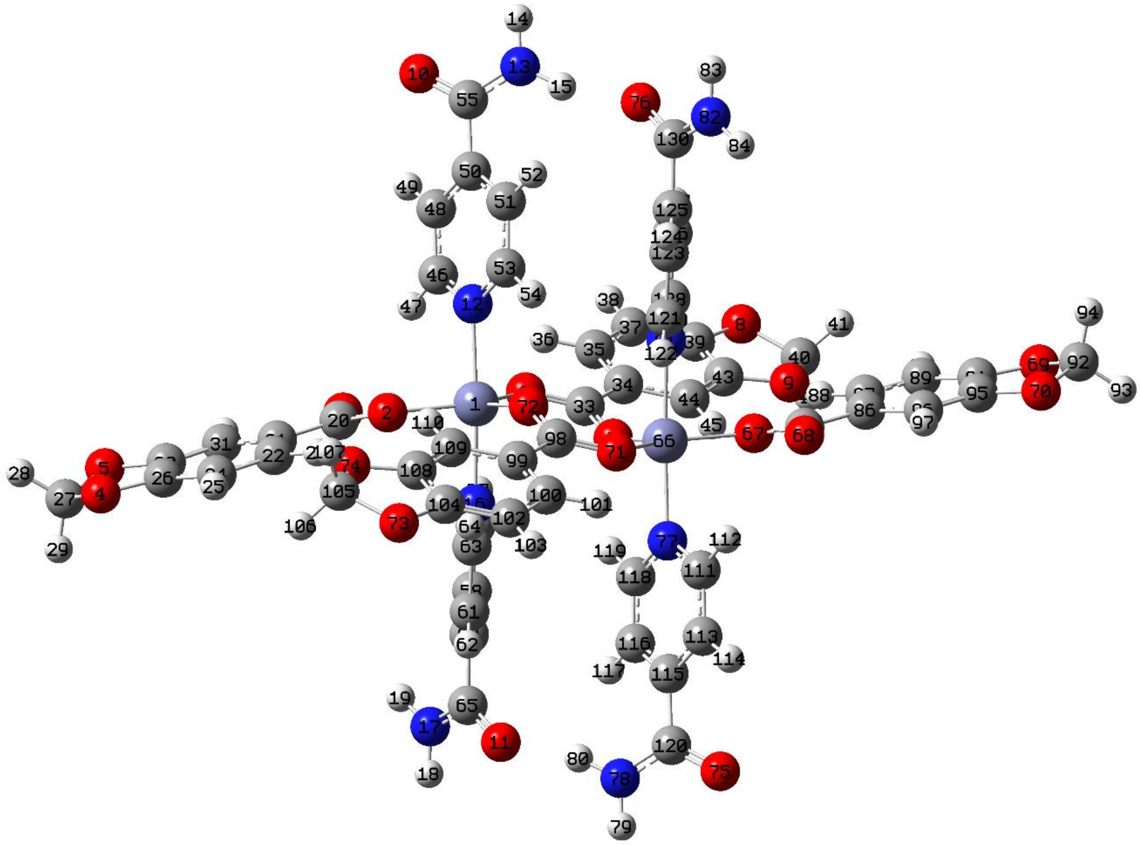

Figure S30. Optimized geometry of the dimer present in **2** in MeOH solution with labelling scheme.

Table S6. Cartesian coordinates from X-ray and optimized geometry of **3** in MeOH.

**Symbol (label)**	**X-Ray structure**			**Optimized geometry**		
	**X**	**Y**	**Z**	**X**	**Y**	**Z**
Cd (1)	6.4062000	10.0801000	-1.0198000	1.4193230	0.1921700	-1.1088940

O (2)	7.0042000	7.7323000	-1.5676000	3.6801720	0.1358730	-0.4577730
O (3)	8.1108000	9.4414000	-2.3992000	3.3662250	-0.1561580	-2.6532500
O (4)	11.0189000	4.6992000	-5.3538000	9.6925210	-1.0519590	-2.0126830
O (5)	9.5918000	3.7591000	-3.7881000	8.7907630	-0.7473090	0.1315270
O (6)	4.5677000	9.5055000	0.2911000	0.9297070	0.1151030	1.2121040
O (7)	2.8282000	9.5355000	1.6258000	-0.1785130	-0.1141560	3.1370850
O (8)	5.2138000	4.3895000	-0.4128000	5.9155840	-0.3026120	2.7740520
O (9)	3.8126000	3.3102000	1.0986000	5.8950310	-0.6019360	5.1002020
O (10)	1.0983000	10.0660000	-6.0091000	1.4429420	-6.9662600	0.0512420
O (11)	10.3533000	7.9527000	4.6143000	2.8380310	7.1770190	0.1594890
N (12)	4.9409000	10.0527000	-2.8338000	1.3193830	-2.1198530	-1.1114640
N (13)	7.6931000	9.8688000	0.9015000	1.5873630	2.4927520	-1.0284810
C (14)	7.9006000	8.1954000	-2.3059000	4.1467350	-0.1095870	-1.6432160
C (15)	8.7705000	7.2827000	-3.1038000	5.6137470	-0.3565880	-1.7915120
C (16)	9.6309000	7.8318000	-4.0569000	6.1598550	-0.5403200	-3.0711680
H (17)	9.6714000	8.7769000	-4.1528000	5.4892150	-0.4906450	-3.9249180
C (18)	10.4300000	7.0370000	-4.8683000	7.5351760	-0.7864500	-3.2526390
H (19)	11.0160000	7.4124000	-5.5150000	7.9729110	-0.9218890	-4.2374730
C (20)	10.3304000	5.6926000	-4.6908000	8.3086920	-0.8487210	-2.1076040
C (21)	10.4501000	3.4748000	-4.8801000	9.9734060	-1.2101110	-0.5858590
H (22)	9.9392000	3.0380000	-5.6058000	10.1414160	-2.2715610	-0.3693900
H (23)	11.1701000	2.8586000	-4.5922000	10.8278070	-0.5893710	-0.3171900
C (24)	9.4761000	5.1285000	-3.7670000	7.7656880	-0.6697830	-0.8343420
C (25)	8.6809000	5.8956000	-2.9433000	6.4273400	-0.4147330	-0.6326170
H (26)	8.1027000	5.5034000	-2.2998000	6.0131110	-0.2660830	0.3600170
C (27)	3.7026000	8.8948000	0.9930000	0.9312480	-0.0642610	2.4916300
C (28)	3.7271000	7.4092000	1.0701000	2.2210590	-0.2145540	3.2268080
C (29)	4.5663000	6.6816000	0.2248000	3.4391630	-0.1853310	2.5039230
H (30)	5.1517000	7.1107000	-0.3878000	3.4758670	-0.0552580	1.4225310
C (31)	4.5034000	5.3111000	0.3232000	4.5901310	-0.3381090	3.2415590
C (32)	4.7571000	3.1080000	0.0263000	6.7528220	-0.6709700	3.9097200
H (33)	5.5218000	2.5659000	0.3461000	7.5660600	0.0465790	4.0096550
H (34)	4.3233000	2.6268000	-0.7216000	7.1102650	-1.6982430	3.7854540
C (35)	3.6715000	4.6716000	1.2122000	4.5836460	-0.5050140	4.6262080
C (36)	2.8666000	5.3539000	2.0769000	3.4046670	-0.5304980	5.3532650
H (37)	2.3159000	4.9043000	2.7072000	3.4091110	-0.6564210	6.4320460
C (38)	2.8940000	6.7525000	1.9849000	2.2090850	-0.3835520	4.6232480
H (39)	2.3346000	7.2660000	2.5550000	1.2486370	-0.3998710	5.1315440
C (40)	3.7400000	9.4531000	-2.8392000	1.5998290	-2.8002260	0.0231200
H (41)	3.4933000	8.9169000	-2.0936000	1.7904790	-2.1958990	0.9032350
C (42)	2.8486000	9.5867000	-3.8843000	1.6312210	-4.1902880	0.0535980
H (43)	2.0081000	9.1449000	-3.8583000	1.8478750	-4.7277830	0.9713850
C (44)	3.1938000	10.3710000	-4.9733000	1.3667240	-4.9053780	-1.1242840
C (45)	4.4590000	10.9272000	-5.0065000	1.0977410	-4.1912540	-2.3015080
H (46)	4.7515000	11.4241000	-5.7605000	0.8880820	-4.6976610	-3.2389980
C (47)	5.2952000	10.7433000	-3.9067000	1.0851410	-2.7945350	-2.2576430
H (48)	6.1619000	11.1314000	-3.9270000	0.8863500	-2.1848780	-3.1344260
C (49)	2.1761000	10.6185000	-6.0614000	1.3529290	-6.4127130	-1.0566860
C (50)	2.5156000	11.5920000	-7.1524000	1.1943550	-7.2090670	-2.3226640

H (51)	3.3748000	11.3467000	-7.5522000	1.9030340	-6.8891150	-3.0968840
H (52)	2.5750000	12.4949000	-6.7766000	0.1731160	-7.0743970	-2.7068100
H (53)	1.8162000	11.5688000	-7.8393000	1.3442960	-8.2686080	-2.0994600
C (54)	8.7657000	10.6415000	1.1057000	1.1688920	3.2782280	-2.0451710
H (55)	8.9282000	11.3547000	0.5007000	0.6415510	2.7702970	-2.8481600
C (56)	9.6493000	10.4538000	2.1537000	1.4127910	4.6527360	-2.0590620
H (57)	10.4051000	11.0190000	2.2580000	1.0495710	5.2521460	-2.8881700
C (58)	9.4084000	9.4246000	3.0442000	2.1120530	5.2284360	-0.9877670
C (59)	8.2807000	8.6339000	2.8518000	2.5462190	4.4007450	0.0586600
H (60)	8.0817000	7.9257000	3.4533000	3.0945050	4.8300700	0.8917100
C (61)	7.4527000	8.8917000	1.7745000	2.2691790	3.0370920	0.0051890
H (62)	6.6817000	8.3511000	1.6518000	2.5950130	2.3458460	0.7759590
C (63)	10.3110000	9.1025000	4.2157000	2.3944690	6.7085690	-0.9009830
C (64)	11.4708000	9.9379000	4.4962000	2.1066990	7.5846490	-2.0897210
H (65)	12.0141000	10.0250000	3.6840000	2.5583610	7.1850020	-3.0065470
H (66)	12.0063000	9.5253000	5.2058000	2.4937500	8.5884390	-1.8952840
H (67)	11.1719000	10.8247000	4.7861000	1.0192610	7.6476980	-2.2363190
Cd (68)	3.6625000	11.6975000	1.0198000	-1.6791840	0.1354920	1.3078080
O (69)	3.0645000	14.0454000	1.5676000	-3.9096440	0.4291400	0.6421200
O (70)	1.9579000	12.3363000	2.3992000	-3.6448990	-0.2402160	2.7586420
O (71)	-0.9502000	17.0784000	5.3538000	-10.0241710	-0.3670720	2.0526820
O (72)	0.4769000	18.0186000	3.7881000	-9.0793680	0.0951840	-0.0429290
O (73)	5.5009000	12.2722000	-0.2911000	-1.1263850	0.2921510	-1.0487280
O (74)	7.2405000	12.2421000	-1.6258000	-0.0438350	0.3440450	-3.0000210
O (75)	4.8549000	17.3882000	0.4128000	-6.1379440	0.3508280	-2.6003930
O (76)	6.2560000	18.4675000	-1.0986000	-6.1399260	0.3618470	-4.9481940
O (77)	8.9704000	11.7117000	6.0091000	-0.6206260	7.0806630	-0.2964160
O (78)	-0.2846000	13.8250000	-4.6143000	-1.7722690	-6.7536840	-0.9432750
N (79)	5.1278000	11.7250000	2.8338000	-1.3733490	2.4204970	1.3101550
N (80)	2.3756000	11.9089000	-0.9015000	-1.6738530	-2.1481660	0.9571080
C (81)	2.1681000	13.5823000	2.3059000	-4.4110480	0.0496700	1.7767150
C (82)	1.2982000	14.4950000	3.1038000	-5.8954460	-0.0584730	1.9019530
C (83)	0.4378000	13.9459000	4.0569000	-6.4669780	-0.3450000	3.1514670
H (84)	0.3973000	13.0008000	4.1528000	-5.8014230	-0.4796170	3.9999350
C (85)	-0.3613000	14.7406000	4.8683000	-7.8621430	-0.4595860	3.3097160
H (86)	-0.9474000	14.3652000	5.5150000	-8.3180420	-0.6852700	4.2694050
C (87)	-0.2617000	16.0851000	4.6908000	-8.6308020	-0.2720680	2.1750650
C (88)	-0.3814000	18.3029000	4.8801000	-10.3336170	0.0989010	0.7011280
H (89)	0.1295000	18.7397000	5.6058000	-10.7245910	1.1217340	0.7555990
H(90)	-1.1014000	18.9190000	4.5922000	-11.0351410	-0.5921840	0.2349660
C (91)	0.5926000	16.6491000	3.7670000	-8.0627300	0.0098680	0.9316250
C (92)	1.3877000	15.8821000	2.9433000	-6.7020240	0.1202810	0.7506960
H (93)	1.9660000	16.2743000	2.2998000	-6.2657390	0.3239230	-0.2223490
C (94)	6.3661000	12.8829000	-0.9930000	-1.1459940	0.3325800	-2.3427420
C (95)	6.3416000	14.3685000	-1.0701000	-2.4457800	0.3510420	-3.0745690
C (96)	5.5023000	15.0960000	-0.2248000	-3.6585440	0.3541430	-2.3422060
H (97)	4.9170000	14.6669000	0.3878000	-3.6865020	0.3512770	-1.2528840
C (98)	5.5653000	16.4665000	-0.3232000	-4.8183580	0.3731680	-3.0822100
C (99)	5.3116000	18.6697000	-0.0263000	-7.0037630	0.4888200	-3.7665890

H (100)	4.5469000	19.2117000	-0.3461000	-7.7398270	-0.3149200	-3.7698290
H (101)	5.7454000	19.1509000	0.7216000	-7.4651420	1.4808340	-3.7658890
C (102)	6.3972000	17.1061000	-1.2122000	-4.8248300	0.3742540	-4.4765650
C (103)	7.2021000	16.4238000	-2.0769000	-3.6509760	0.3667410	-5.2123810
H (104)	7.7528000	16.8733000	-2.7072000	-3.6655400	0.3656800	-6.2983800
C (105)	7.1747000	15.0252000	-1.9849000	-2.4473650	0.3575700	-4.4814100
H (106)	7.7340000	14.5117000	-2.5550000	-1.4906300	0.3549190	-4.9968200
C (107)	6.3287000	12.3246000	2.8392000	-1.8072140	3.1037080	0.2274620
H (108)	6.5753000	12.8607000	2.0936000	-2.3750320	2.5319690	-0.4988860
C (109)	7.2200000	12.1909000	3.8843000	-1.5254940	4.4568220	0.0579170
H (110)	8.0606000	12.6327000	3.8583000	-1.8571790	4.9933940	-0.8254300
C (111)	6.8748000	11.4067000	4.9733000	-0.7907900	5.1296570	1.0454250
C (112)	5.6097000	10.8505000	5.0065000	-0.3725350	4.4183100	2.1807960
H (113)	5.3172000	10.3536000	5.7605000	0.1981840	4.8945760	2.9727140
C (114)	4.7735000	11.0343000	3.9067000	-0.6754420	3.0591620	2.2733560
H (115)	3.9068000	10.6462000	3.9270000	-0.3608660	2.4500530	3.1162370
C (116)	7.8926000	11.1591000	6.0614000	-0.4525180	6.5822950	0.8291810
C (117)	7.5531000	10.1857000	7.1524000	0.1123560	7.3894040	1.9661990
H (118)	6.6939000	10.4309000	7.5522000	-0.4519960	7.2388560	2.8945530
H (119)	7.4936000	9.2828000	6.7766000	1.1579040	7.0945490	2.1403220
H (120)	8.2525000	10.2089000	7.8393000	0.1048840	8.4475250	1.6916540
C (121)	1.3030000	11.1362000	-1.1057000	-1.4401680	-2.9921630	1.9855680
H (122)	1.1405000	10.4229000	-0.5007000	-1.2307970	-2.5253930	2.9437480
C (123)	0.4193000	11.3239000	-2.1537000	-1.4581080	-4.3787860	1.8143450
H (124)	-0.3364000	10.7586000	-2.2580000	-1.2482100	-5.0237530	2.6625390
C (125)	0.6602000	12.3530000	-3.0442000	-1.7276740	-4.9023530	0.5408480
C (126)	1.7880000	13.1437000	-2.8518000	-1.9938640	-4.0140370	-0.5118550
H (127)	1.9870000	13.8520000	-3.4533000	-2.2099720	-4.4028770	-1.5018460
C (128)	2.6160000	12.8860000	-1.7745000	-1.9606480	-2.6453490	-0.2675080
H (129)	3.3870000	13.4265000	-1.6518000	-2.1467440	-1.9120890	-1.0440120
C (130)	-0.2423000	12.6752000	-4.2157000	-1.7057180	-6.3804090	0.2393740
C (131)	-1.4021000	11.8398000	-4.4962000	-1.5630990	-7.3657230	1.3666070
H (132)	-1.9454000	11.7527000	-3.6840000	-2.2662220	-7.1586030	2.1828590

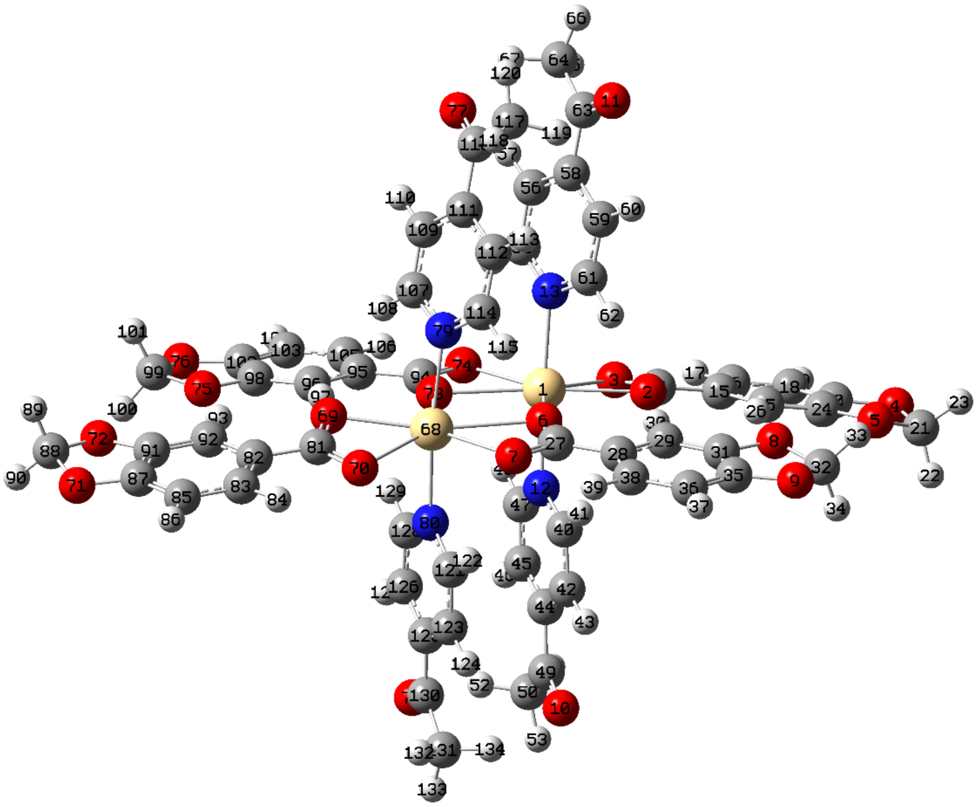

Figure S31. Optimized geometry of **3** in MeOH solution with labelling scheme.

Table S7. Cartesian coordinates from X-ray and optimized geometry of **4** in MeOH.

Symbol (label)	X-Ray structure			Optimized geometry		
	X	Y	Z	X	Y	Z
Cd (1)	4.5049000	13.5467000	6.4296000	1.4262150	0.1265300	-1.0268780
O (2)	3.9192000	13.7625000	8.7744000	3.6021480	-0.6554700	-0.6077800
O (3)	2.3180000	13.6043000	7.2990000	3.4212540	-0.0864560	-2.7698980
O (4)	-0.9570000	14.1009000	12.7000000	9.5246710	-2.0470580	-2.2943980
O (5)	1.2992000	14.0321000	13.2350000	8.5724680	-2.1228350	-0.1533970
O (6)	6.7941000	13.5967000	6.8979000	0.9656140	-0.2766990	1.2354450
O (7)	8.9795000	13.4903000	6.6660000	-0.0922990	-0.2686000	3.1988350
O (8)	6.3176000	13.5727000	12.0848000	5.8630790	-1.5243990	2.5972340
O (9)	8.4643000	13.5782000	12.9590000	5.9042830	-1.7244550	4.9335870
O (10)	3.7934000	20.7274000	5.8231000	-1.6177220	-6.2346150	-2.7622280
O (11)	5.3151000	6.3846000	7.4302000	5.4922970	5.9696030	0.1074480
N (12)	4.6368000	15.8739000	6.4571000	0.8000570	-2.0430870	-1.4723440
N (13)	5.5076000	20.8236000	7.3003000	0.2388230	-7.0816140	-1.7261850
H (14)	5.5063000	21.7034000	7.3140000	-0.0953350	-8.0285900	-1.8306790
H (15)	6.0858000	20.3766000	7.7904000	1.1477960	-6.9446560	-1.3124640
N (16)	4.6282000	11.2441000	6.4792000	2.1970580	2.2553730	-0.5997650
N (17)	4.1116000	6.2932000	5.5460000	3.6139570	7.1322600	-0.5039300
H (18)	4.1536000	5.4148000	5.5138000	4.1157050	8.0073400	-0.4526680
H (19)	3.6786000	6.7362000	4.9211000	2.6026820	7.1619760	-0.6293230
C (20)	2.7012000	13.7433000	8.4891000	4.1141150	-0.5267220	-1.7907500

C (21)	1.6869000	13.8594000	9.5791000	5.5508600	-0.9094450	-1.9786620
C (22)	0.3336000	13.9381000	9.2681000	6.1270380	-0.8688660	-3.2579780
H (23)	0.0703000	13.9282000	8.3552000	5.5118940	-0.5505620	-4.0951220
C (24)	-0.6476000	14.0308000	10.2504000	7.4735560	-1.2287430	-3.4709190
H (25)	-1.5696000	14.1016000	10.0345000	7.9232990	-1.2071580	-4.4593070
C (26)	-0.2067000	14.0153000	11.5456000	8.1952240	-1.6129170	-2.3551980
C (27)	-0.0543000	13.8111000	13.7601000	9.8517590	-2.1429730	-0.8657430
H (28)	-0.1601000	12.8726000	14.0564000	10.4457500	-1.2717690	-0.5750470
H (29)	-0.2260000	14.4073000	14.5318000	10.3595750	-3.0859310	-0.6746710
C (30)	1.1249000	13.9564000	11.8622000	7.6253220	-1.6508770	-1.0805740
C (31)	2.1192000	13.8734000	10.9105000	6.3100600	-1.3131510	-0.8509390
H (32)	3.0392000	13.8271000	11.1436000	5.8715400	-1.3500850	0.1420250
C (33)	7.9627000	13.5661000	7.3826000	0.9864880	-0.4305040	2.5134860
C (34)	8.1312000	13.6234000	8.8805000	2.2608050	-0.7838490	3.2028260
C (35)	7.0036000	13.6031000	9.7133000	3.4296320	-0.9915950	2.4274080
H (36)	6.1202000	13.5806000	9.3634000	3.4277450	-0.9003580	1.3423880
C (37)	7.2502000	13.6173000	11.0640000	4.5741870	-1.3142140	3.1193220
C (38)	7.0519000	13.6156000	13.2922000	6.6773220	-2.0069690	3.7146180
H (39)	6.8151000	12.8422000	13.8624000	7.6145820	-1.4553940	3.7467770
H (40)	6.8409000	14.4466000	13.7887000	6.8200220	-3.0870560	3.6242550
C (41)	8.5273000	13.6329000	11.5873000	4.6065440	-1.4257480	4.5103370
C (42)	9.6300000	13.6813000	10.7945000	3.4793340	-1.2219660	5.2878790
H (43)	10.5028000	13.7179000	11.1679000	3.5119180	-1.3064710	6.3700410
C (44)	9.4354000	13.6744000	9.4066000	2.2911200	-0.8984200	4.6033880
H (45)	10.1824000	13.7058000	8.8215000	1.3740830	-0.7318740	5.1611300
C (46)	3.8042000	16.5918000	5.7135000	0.1814450	-2.3793310	-2.6285820
H (47)	3.1928000	16.1386000	5.1450000	0.0258720	-1.5760540	-3.3422070
C (48)	3.7937000	17.9755000	5.7349000	-0.2429070	-3.6806090	-2.8829700
H (49)	3.1776000	18.4572000	5.1946000	-0.7571880	-3.9245070	-3.8069040
C (50)	4.6748000	18.6452000	6.5399000	-0.0105340	-4.6683250	-1.9165390
C (51)	5.5595000	17.9087000	7.3098000	0.6334850	-4.3170060	-0.7223520
H (52)	6.1956000	18.3396000	7.8678000	0.8030560	-5.0419430	0.0685090
C (53)	5.4898000	16.5224000	7.2445000	1.0219230	-2.9906060	-0.5349170
H (54)	6.0797000	16.0139000	7.7880000	1.5041600	-2.6583780	0.3783830
C (55)	4.6375000	20.1713000	6.5407000	-0.5243450	-6.0618400	-2.1683640
C (56)	5.5227000	10.5561000	7.2204000	3.1264380	2.4401690	0.3698160
H (57)	6.1524000	11.0411000	7.7399000	3.3237630	1.5905940	1.0153690
C (58)	5.5578000	9.1712000	7.2548000	3.8035260	3.6477820	0.5171900
H (59)	6.1975000	8.7181000	7.7913000	4.5588610	3.7665160	1.2872890
C (60)	4.6432000	8.4559000	6.4926000	3.5052000	4.7050350	-0.3545530
C (61)	3.7081000	9.1611000	5.7322000	2.5355290	4.5130930	-1.3476200
H (62)	3.0643000	8.7015000	5.2057000	2.2777090	5.3001760	-2.0482900
C (63)	3.7395000	10.5466000	5.7633000	1.9043190	3.2726510	-1.4420650
H (64)	3.0972000	11.0244000	5.2510000	1.1610180	3.0600730	-2.2041690
C (65)	4.6906000	6.9377000	6.5191000	4.2810360	5.9934500	-0.2296790
Cd (66)	7.8389000	13.5467000	4.6007000	-1.5801250	0.2619940	1.4000260
O (67)	8.4247000	13.7625000	2.2559000	-3.8102140	0.6584960	0.9524600
O (68)	10.0259000	13.6043000	3.7313000	-3.7863260	-0.2262660	3.0203690
O (69)	13.3009000	14.1009000	-1.6698000	-10.0544410	-0.0910160	1.6761900

O (70)	11.0446000	14.0321000	-2.2047000	-8.9071620	0.7201250	-0.1997390
O (71)	5.5498000	13.5967000	4.1324000	-1.1444890	0.4661380	-0.9431390
O (72)	3.3644000	13.4903000	4.3642000	0.0149590	1.0193760	-2.7685840
O (73)	6.0263000	13.5727000	-1.0545000	-6.0341890	1.7598270	-2.2947740
O (74)	3.8795000	13.5782000	-1.9288000	-5.9737000	2.4549170	-4.5318950
O (75)	8.5504000	20.7274000	5.2072000	-2.3696650	-6.9763050	0.7432150
O (76)	7.0288000	6.3846000	3.6001000	0.7528730	6.9106250	-0.2213710
N (77)	7.7071000	15.8739000	4.5732000	-1.9228270	-1.9738390	0.9394340
N (78)	6.8363000	20.8236000	3.7300000	-4.2566920	-6.2788660	-0.3481200
H (79)	6.8375000	21.7034000	3.7162000	-4.5396210	-7.2294710	-0.5366530
H (80)	6.2580000	20.3766000	3.2399000	-4.8722480	-5.5357180	-0.6394140
N (81)	7.7157000	11.2441000	4.5511000	-0.9222740	2.4696640	1.4068920
N (82)	8.2323000	6.2932000	5.4843000	0.9822610	7.1256790	2.0409810
H (83)	8.1903000	5.4148000	5.5165000	1.3886330	8.0452700	1.9405440
H (84)	8.6653000	6.7362000	6.1092000	0.8094680	6.7885020	2.9757930
C (85)	9.6427000	13.7433000	2.5412000	-4.4236710	0.1829630	1.9969380
C (86)	10.6570000	13.8594000	1.4511000	-5.9216930	0.1032140	1.9564110
C (87)	12.0102000	13.9381000	1.7622000	-6.6177480	-0.3866880	3.0734820
H (88)	12.2736000	13.9282000	2.6751000	-6.0471130	-0.6947550	3.9449080
C (89)	12.9915000	14.0308000	0.7798000	-8.0236340	-0.4844550	3.0828680
H (90)	13.9135000	14.1016000	0.9957000	-8.5661300	-0.8682190	3.9419850
C (91)	12.5506000	14.0153000	-0.5153000	-8.6798820	-0.0667100	1.9392960
C (92)	12.3981000	13.8111000	-2.7298000	-10.2342980	0.6354890	0.4128500
H (93)	12.5040000	12.8726000	-3.0262000	-10.6026200	1.6427580	0.6279040
H (94)	12.5698000	14.4073000	-3.5015000	-10.8993190	0.0702850	-0.2366030
C (95)	11.2189000	13.9564000	-0.8319000	-7.9915310	0.4218670	0.8267320
C (96)	10.2246000	13.8734000	0.1198000	-6.6172510	0.5181360	0.7928820
H (97)	9.3047000	13.8271000	-0.1133000	-6.0935550	0.8938410	-0.0804570
C (98)	4.3812000	13.5661000	3.6477000	-1.1073130	0.9244020	-2.1466110
C (99)	4.2126000	13.6234000	2.1498000	-2.3713800	1.3323960	-2.8250940
C (100)	5.3403000	13.6031000	1.3170000	-3.5914860	1.2814270	-2.1035750
H (101)	6.2237000	13.5806000	1.6669000	-3.6336580	0.9664020	-1.0610560
C (102)	5.0937000	13.6173000	-0.0338000	-4.7210270	1.6690460	-2.7875670
C (103)	5.2919000	13.6156000	-2.2619000	-6.8831360	2.0362970	-3.4549750
H (104)	5.5288000	12.8422000	-2.8321000	-7.5643540	2.8516350	-3.2212400
H (105)	5.5029000	14.4466000	-2.7584000	-7.3995900	1.1213390	-3.7573020
C (106)	3.8165000	13.6329000	-0.5570000	-4.6888420	2.0944630	-4.1168570
C (107)	2.7139000	13.6813000	0.2358000	-3.5089800	2.1577380	-4.8375320
H (108)	1.8410000	13.7179000	-0.1377000	-3.4896180	2.4951680	-5.8694970
C (109)	2.9085000	13.6744000	1.6237000	-2.3371580	1.7640580	-4.1620710
H (110)	2.1615000	13.7058000	2.2088000	-1.3811380	1.7903770	-4.6768730
C (111)	8.5396000	16.5918000	5.3168000	-1.7095680	-2.9148740	1.8892540
H (112)	9.1510000	16.1386000	5.8853000	-1.2375060	-2.5725350	2.8048610
C (113)	8.5502000	17.9755000	5.2954000	-2.0788690	-4.2445840	1.7027870
H (114)	9.1662000	18.4572000	5.8357000	-1.8865620	-4.9849220	2.4726630
C (115)	7.6691000	18.6452000	4.4904000	-2.7070080	-4.6108540	0.5048610
C (116)	6.7844000	17.9087000	3.7205000	-2.9145070	-3.6365100	-0.4812420
H (117)	6.1483000	18.3396000	3.1625000	-3.3560610	-3.8853160	-1.4418930
C (118)	6.8540000	16.5224000	3.7858000	-2.5039130	-2.3273020	-0.2289570

H (119)	6.2641000	16.0139000	3.2422000	-2.6213890	-1.5356330	-0.9614580
C (120)	7.7063000	20.1713000	4.4895000	-3.0982340	-6.0522250	0.3045400
C (121)	6.8211000	10.5561000	3.8099000	-1.4157770	3.2973590	0.4543920
H (122)	6.1914000	11.0411000	3.2904000	-2.1983510	2.8931300	-0.1786070
C (123)	6.7860000	9.1712000	3.7754000	-0.9404850	4.5948030	0.2880410
H (124)	6.1464000	8.7181000	3.2390000	-1.3359260	5.2317660	-0.4963280
C (125)	7.7006000	8.4559000	4.5376000	0.0661850	5.0591460	1.1454710
C (126)	8.6358000	9.1611000	5.2981000	0.5695660	4.2041950	2.1347400
H (127)	9.2796000	8.7015000	5.8246000	1.3707550	4.5087330	2.8018530
C (128)	8.6044000	10.5466000	5.2670000	0.0496000	2.9137500	2.2330890
H (129)	9.2467000	11.0244000	5.7793000	0.4086940	2.2132500	2.9796000
C (130)	7.6533000	6.9377000	4.5112000	0.6179490	6.4427110	0.9432300

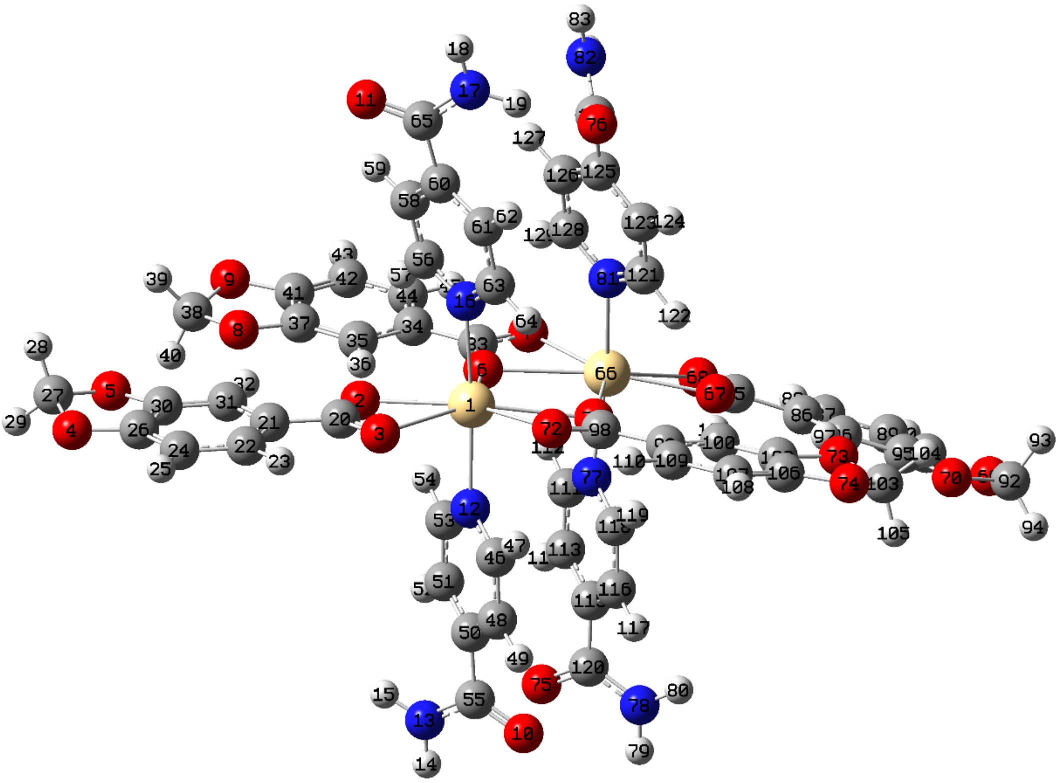

Figure S32. Optimized geometry of **4** in MeOH solution with labelling scheme.

References

(1) Pinsky, M.; Avnir, D. Continuous Symmetry Measures. 5. The Classical Polyhedra. *Inorg. Chem.* **1998**, *37*, 5575–5582.

(2) Llunell, M.; Casanova, D.; Cirera, J.; Bofill, J. M.; Alemany, P.; Alvarez, S.; Pinsky, M.; Avnir, D. SHAPE Version 2.1. Universitat de Barcelona and The Hebrew University of Jerusalem 2013.
